# Potassium levels and the risk of all-cause and cardiovascular mortality among patients with cardiovascular diseases: a meta-analysis of cohort studies

**DOI:** 10.1186/s12937-023-00888-z

**Published:** 2024-01-10

**Authors:** Yahui Fan, Min Wu, Xiaohui Li, Jinping Zhao, Jia Shi, Lu Ding, Hong Jiang, Zhaofang Li, Wei Zhang, Tianyou Ma, Duolao Wang, Le Ma

**Affiliations:** 1 School of Public Health, Xi’an Jiaotong University Health Science Center, Xi’an, 710061 China; 2 The First Affiliated Hospital, Xi’an Jiaotong University Health Science Center, Xi’an, 710061 China; 3 Department of Maternal and Child Health Management, Sichuan Provincial Maternity and Child Health Care Hospital, Chengdu, 610045 China; 4 Key Laboratory for Disease Prevention and Control and Health Promotion of Shaanxi Province, Xi’an, 710061 China; 5 Key Laboratory of Environment and Genes Related to Diseases (Xi’an Jiaotong University), Ministry of Education of China, Xi’an, 710061 China; 6 Guangdong Key Laboratory of Age-Related Cardiac and Cerebral Diseases, Affiliated Hospital of Guangdong Medical University, Zhanjiang, 524013 China; 7 Department of Clinical Sciences, Liverpool School of Tropical Medicine, Liverpool, L3 5QA UK

**Keywords:** Potassium, Cardiovascular Disease, Mortality, Meta-analysis

## Abstract

**Background:**

Abnormal blood potassium levels are associated with an increased risk of cardiometabolic diseases and mortality in the general population; however, evidence regarding the association between dyskalemia and mortality among patients with cardiovascular disease (CVD) remains inconclusive. This study aimed to evaluate the association of potassium levels with all-cause and cardiovascular mortality among patients with CVD.

**Methods:**

PubMed, Embase, Web of Science, and Cochrane Library databases were searched up to August 2023 to identify relevant cohort studies among patients with CVD, such as myocardial infarction, stroke, and heart failure. Abnormal potassium levels were considered as hypokalemia or hyperkalemia. The primary outcomes were all-cause mortality based on follow-up length (including in-hospital, short-term and long-term mortality) and cardiovascular mortality. The methodological quality of included studies was assessed by using the Newcastle-Ottawa Scale. The pooled relative risks (RRs) and 95% confidence intervals (CIs) were calculated using random-effects models. Restricted cubic splines were applied to explore the dose-response relationship.

**Results:**

Thirty-one cohort studies involving 227,645 participants with an average age of 68.3 years were included in the meta-analysis, all of which achieved moderate to high quality. Hyperkalemia was significantly associated with an approximately 3.0-fold increased risk of all-cause in-hospital mortality (RR:2.78,95CI%:1.92,4.03), 1.8-fold of all-cause short-term mortality (RR:1.80, 95CI%:1.44,2.27), 1.3-fold of all-cause long-term mortality (RR:1.33, 95CI%:1.19,1.48) and 1.2-fold of cardiovascular mortality (RR:1.19, 95CI%:1.04,1.36). Similar positive associations were also observed between hypokalemia and risk of all-cause mortality and cardiovascular mortality. The RRs of all-cause in-hospital, short-term, long-term mortality and cardiovascular mortality with hyperkalemia were attenuated to 2.21 (95CI%:1.60,3.06), 1.46(95CI%:1.25,1.71), 1.23 (95CI%:1.09,1.39) and 1.13 (95CI%:1.00,1.27) when treating hypokalemia together with normokalemia as the reference group. A U-shaped association was observed between potassium levels and mortality, with the lowest risk at around 4.2 mmol/L.

**Conclusions:**

Both hypokalemia and hyperkalemia were positively associated with the risk of mortality in patients with CVD. Our results support the importance of potassium homeostasis for improving the CVD management.

**Registration:**

PROSPERO, CRD42022324337.

**Supplementary Information:**

The online version contains supplementary material available at 10.1186/s12937-023-00888-z.

## Introduction

Potassium, as a predominant intracellular cation, has been considered to be pivotal in multiple physiological processes related to human health [1]. Abnormalities in potassium levels could impair the excitability of cells, particularly cardiac myocytes via perturbation of the electric potential across cell membranes [2]. Increasing evidence has emphasized the harmful effects of hypokalemia and hyperkalemia on cardiac electrophysiology, which subsequently results in malignant arrhythmias and sudden cardiac death [3, 4]. Correction of hypokalemia and hyperkalemia might delay the onset and progression of cardiovascular disease (CVD) by modulating oxidative stress, inflammation and vascular remodeling [5, 6]. Patients with CVD may be predisposed to dyskalemia attributable to comorbid conditions and treatment side effects compared to the general population [7, 8]. Consequently, the maintenance of potassium homeostasis is essential for patients with CVD to confer a substantial health benefit.

The health hazards associated with dyskalemia have been extensively investigated in the general population, suggesting that abnormal potassium levels increase the risk of cardiometabolic diseases and mortality [9–11]. Patients with chronic kidney disease (CKD) and CVD are at risk for developing morbidities and premature death, and the disturbance of potassium homeostasis may induce poor prognosis and unfavorable survival among patients with preexisting these diseases that shared some pathophysiological mechanisms and risk factors. A previous meta-analysis of cohort studies revealed that hypokalemia and hyperkalemia were associated with a higher risk of progression of CKD [12]. Several studies have examined the association between hypokalemia and hyperkalemia and adverse outcomes among patients with CVD, which yielded inconsistent findings [13–15]. Two previous meta-analyses evaluating the association between potassium level and mortality in patients with CVD focused on myocardial infarction (MI) survivors [16, 17]. However, such association among patients with total and other individual CVD events (heart failure [HF] and stroke) remains unclear. Furthermore, hypokalemia was regarded as safe in some studies, which treated hypokalemia together with normokalemia as the reference category [18–20]. The potential harmful impacts of hypokalemia on health still needs to be validated. In addition, it is still unknown whether any dose-response relation exists between potassium level and mortality.

Therefore, we aimed to perform a meta-analysis to examine the association of dyskalemia with the risk of all-cause and cardiovascular mortality in patients with CVD, and to further quantify the dose-response relationship between potassium level and mortality.

## Methods

The reporting of the meta-analysis followed the Preferred Reporting Items of Systematic Reviews and Meta-Analysis (PRISMA) statement guideline [21] (Additional file 1). The priori protocol was registered in PROSPERO (CRD42022324337).

### Eligibility criteria

Studies were considered appropriate for inclusion if they met the following eligibility criteria guided by the PECOs: (1) studies enrolled adult patients with CVD, including MI and HF (Population). Patients with stroke were also considered in the present study, but no available studies were retrieved; (2) the exposures of interest were hypokalemia or hyperkalemia (Exposure). Blood (serum/plasma) potassium levels were reported as categorical variables with cut-off values ≤ 4.0 mmol/L or lower for hypokalemia and ≥ 4.5 mmol/L or higher for hyperkalemia; (3) the comparator of interest was normokalemia (Comparator), which was set as 4.0-4.5 mmol/L or closely approximated range; (4) the endpoints of interest were all-cause mortality and cardiovascular mortality (Outcome). The all-cause mortality included all-cause in-hospital, short-term (< 6 months) and long-term (≥ 1 year) mortality based on duration of follow-up; (5) studies published as original articles used a cohort study design or a post hoc analysis of clinical trial (Study).

### Search strategy

PubMed, Embase, Web of Science and Cochrane Library databases were searched systematically from inception up to August 2023 to retrieve relevant studies measuring the association of blood potassium levels with mortality among individuals with CVD. The combinations of Medical Subject Heading terms and text word were as follows: “potassium”, AND “cardiovascular disease”, “coronary heart disease”, “myocardial infarction”, “stroke”, “heart failure”, AND “mortality” (Table 1 in Additional file 2). Searches were not restricted by publication language or date. To capture potentially unidentified citations, a manual review of bibliographies from included studies, relevant reviews and meta-analyses was conducted. The authors and experts in the field were contacted and consulted for extra information by e-mail.

### Study selection

Firstly, all literature search results were imported into EndNote X9, and duplicate records will be removed prior to screening. Secondly, two reviewers independently examined the titles and abstracts of the records to ascertain relevance (YHF & MW). Thirdly, the full-text review of remaining articles were performed based on the eligibility criteria after verification of the titles and abstracts. The excluded studies were organized together, with reasons for exclusions, and a comprehensive list of citations excluded after assessment of the full-text was provided in Table 2 in Additional file 3. In case of any disagreements in the study selection process, resolve it through discussion and consensus with a third reviewer (LM).

### Data extraction and quality assessment

The following data were extracted using a standardized data collection form: name of the first author, publication year, geographic region, study design, follow-up period, characteristics of participants (mean age, sex, and CVD subtype), categories of blood potassium levels, mean/median potassium levels in each category, type of blood sample, total number of participants, number of deaths, outcome ascertainment method, relative risks (RRs) with corresponding 95% confidence intervals (CIs), and confounding variables adjusted in the analysis. The potassium levels were considered as hypokalemia, normokalemia (reference) and hyperkalemia in most of the included studies. In cases where multiple RRs were reported, the RR with the greatest degree of adjustment was retained.

The Newcastle-Ottawa Scale was applied to evaluate the methodological quality of the selected studies based on three domains: participant selection (4 stars), group comparability (2 stars), and outcome assessment (3 stars) [22]. Studies scoring 7–9 points were judged as high quality, 4–6 points as medium quality and 0–3 low quality. Three investigators (YHF & MW & XHL) independently extracted the data and conducted the study quality assessment, with a consensus reached by discussion or third-party arbitration (DLW).

### Statistical analysis

The study-specific RRs of the association between hypokalemia, hyperkalemia and all-cause and cardiovascular mortality among patients with CVD were pooled. The RRs with corresponding 95% CIs were provided or could be calculated. When multiple publications performed on the same sample were available, the article with the largest number of participants or longest follow-up was chosen. For the primary analysis, we reported the pooled analyses separately for all-cause in-hospital, short-term and long-term mortality. Random-effect models were used to combine the data considering clinical and statistical variance across studies. When studies had multiple subcategories of hypokalemia or hyperkalemia, the combined RRs were generated using fixed-effect models. Cochran’s *Q* test with significant level of P < 0.10 was calculated to test heterogeneity, and the *I*^*2*^ statistic was used to quantify the degree of heterogeneity, where *I*^*2*^ more than 50% indicated substantial heterogeneity [23]. In order to convert hypokalemia together with normokalemia to be the reference group, the RRs and 95% CIs were recalculated using the Hamling method and pooled in secondary analysis [24]. Moreover, the associations among patients with different types of CVD were also explored. To explore potential sources of heterogeneity across studies, subgroup analyses and meta-regression were performed based on several predefined factors, including the mean age of participants (< 65 or ≥ 65 years), country of origin (Asia, North America, Europe or Multi-continents), study design (prospective or retrospective), times of potassium measurement (once or multiple times or not reported), type of blood sample (serum, plasma or serum and plasma), adjustment for gender (yes or no), renal function (yes or no), hypertension (yes or no) and cardiovascular medications (yes or no). The dose-response meta-analysis was performed based on a generalized least squares regression model, as described by Greenland and Longnecker [25], and studies were included if the RRs were reported for at least three categories of potassium levels. Accordingly, the corresponding dose in each category was the mean or median of potassium level. The midpoint of the lower and upper bounds was estimated for studies reporting potassium ranges. When the extreme potassium level was open-ended, the width of the adjacent interval was used to obtain the assigned dose. In addition, if the number of person-years or events by each category was not available, the methods proposed by Bekkering were applied to impute these required data [26]. The potential non-linear association was explored by restricted cubic splines method. Additionally, sensitivity analyses were conducted to test the robustness of our results. First, the sensitivity analyses by sequential removal of each study were performed to assess the influence of any single study on the summary results. Second, to examine whether the difference in the potassium levels for hypokalemia and hyperkalemia affected the associations, we restricted the analyses to studies that used potassium levels < 3.5 mmol/L to define hypokalemia and > 5.0 mmol/L to define hyperkalemia, respectively. Publication bias was assessed by visual appreciation of funnel plots if ≥ 10 studies were available [27], and further tested using Begg’s and Egger’s tests [28, 29]. The trim-and-fill method was used to correct publication bias by recomputing the combined effect. Analyses were undertaken using Stata version 12.0 (Stata Corp, College Station, TX, USA). A 2-tailed P < 0.05 represent statistical significance unless otherwise specified.

## Results

### Literature search

**Fig. 1:**
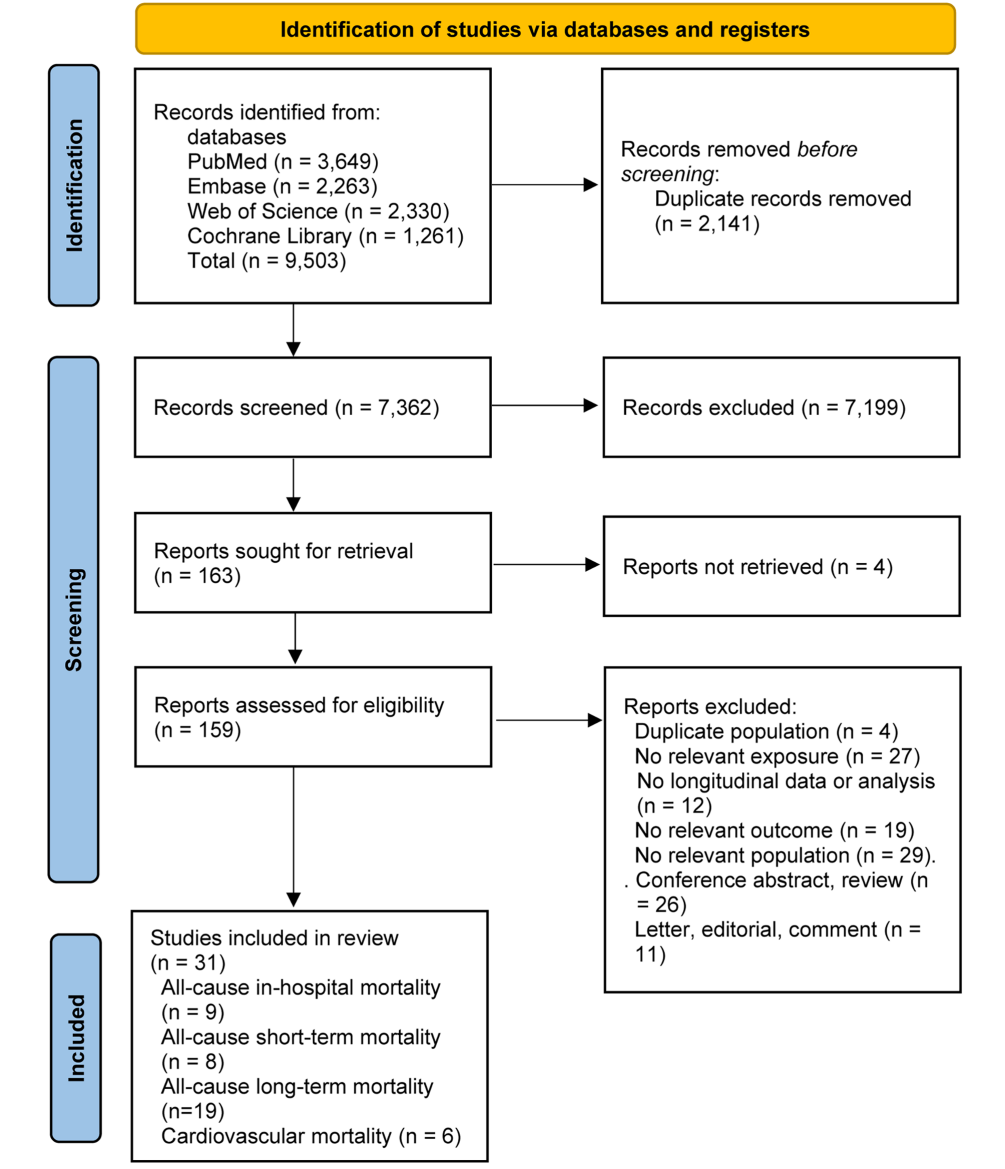
Flow diagram of study selection

The initial systematic search of the electronic databases yielded 9,503 records. After removing duplicates, 7,362 articles were screened for eligibility based on titles and abstracts, of which 159 articles qualified for further detailed full-text review. Finally, 31 studies were identified and included in the meta-analysis [4, 13–15, 30–56], and one study [51] could only be included in the dose-response meta-analysis. The flow diagram of study selection process is shown in Fig. 1.

### Study characteristics

The characteristics of the included studies are summarized in Additional file 4. The sample size of each selected study ranged from 298 to 38,689, comprising 227,645 participants, 19,357 total deaths and 4,965 cardiovascular deaths (three studies did not report number of deaths [38, 42, 43]). 16 studies were retrospective cohort studies, and 15 had a prospective study design including 13 cohort studies and two post hoc analysis of clinical trials. The targeted study population in 18 studies involved patients with HF, 12 enrolled MI survivors, and one included patients with unspecified CVD. 11 studies were conducted in Asia, ten studies in Europe, six in North America and the remaining four were multinational studies. The mean age of participants of the included studies varied from 58.0 to 83.0 years. All studies comprised both men and women. The majority of included studies defined hypokalemia and hyperkalemia as blood potassium levels < 3.5 mmol/L (n = 25) and blood potassium levels > 5.0 mmol/L (n = 24), respectively, whereas blood potassium levels < 4.0 mmol/L and > 4.5 mmol/L or > 5.5 mmol/L were set as hypokalemia and hyperkalemia, respectively in four studies. 29 studies measured potassium concentration in serum, whereas the plasma potassium concentration was assessed in one studies and one study reported potassium concentration from serum or plasma. Potassium levels were assessed once in two studies and multiple times in 11 studies, and the other studies did not report the number of potassium measurement times (n = 18). Except for studies focusing on deaths during hospitalization (n = 5), follow-up durations were variable from 1.0 month to 8.1 years. Deaths were confirmed by reviewing medical records (n = 20), death registries (n = 12), or telephone interviewing (n = 9). Potential confounders for adjustment included age (n = 30), gender (n = 26), cardiovascular medication (n = 21) and renal function (n = 19). Most studies (n = 29) were judged to be high quality with the others rated as medium quality (Table 4 in Additional file 5).

### Association of hypokalemia with all-cause and cardiovascular mortality

**Fig. 2:**
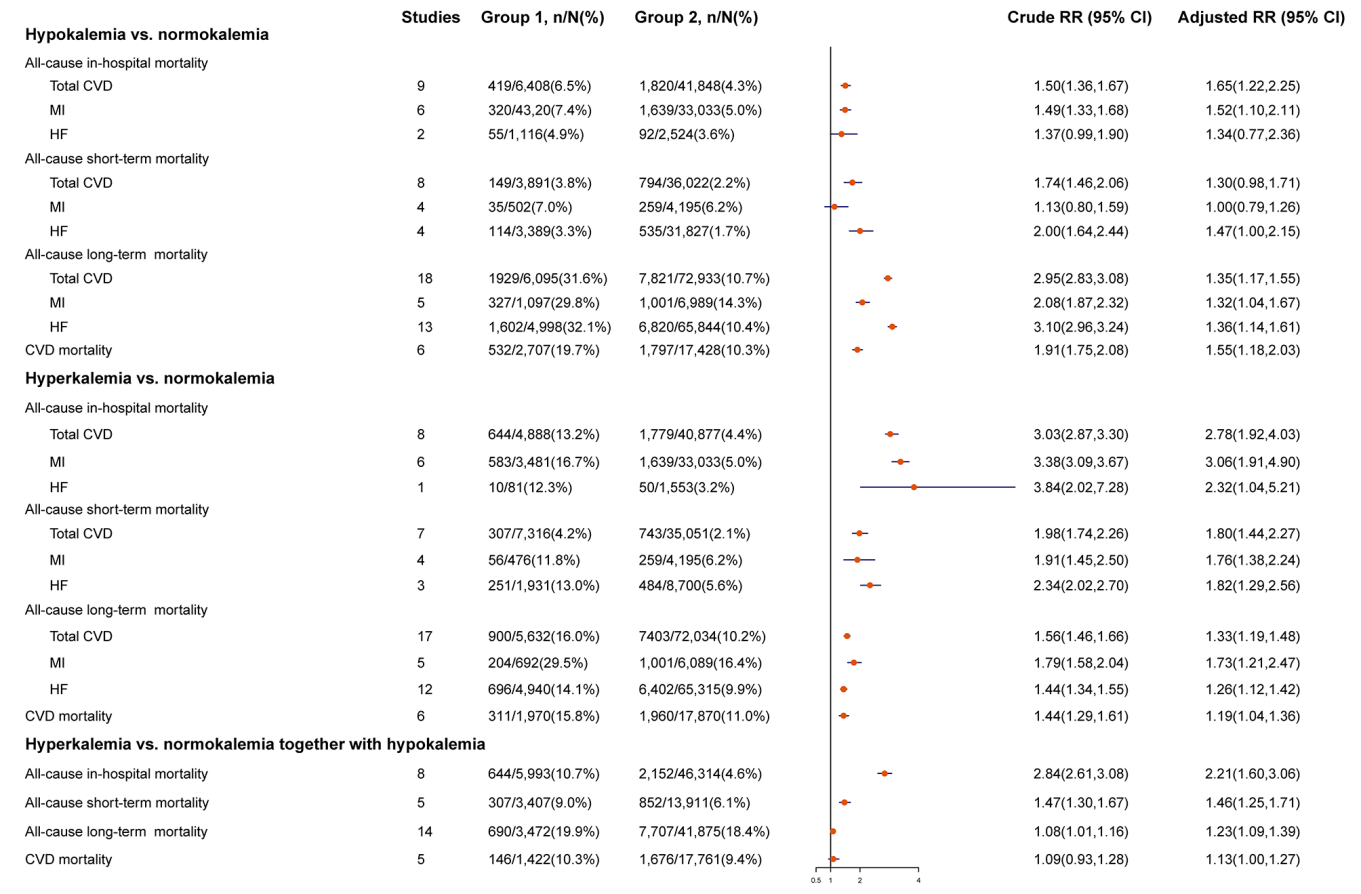
Forest plot for association between blood potassium levels and risk of mortality in patients with total and individual cardiovascular diseases expressed as comparison between hypokalemia, hyperkalemia and normokalemia; hyperkalemia and normokalemia together with hypokalemia. The diamond indicates the pooled RR estimates from random effects analysis, and horizontal lines indicate corresponding 95% CIs. CI, confidence interval; CVD, cardiovascular disease; HF, heart failure; MI, myocardial infarction; RR, relative risk

**Fig. 3:**
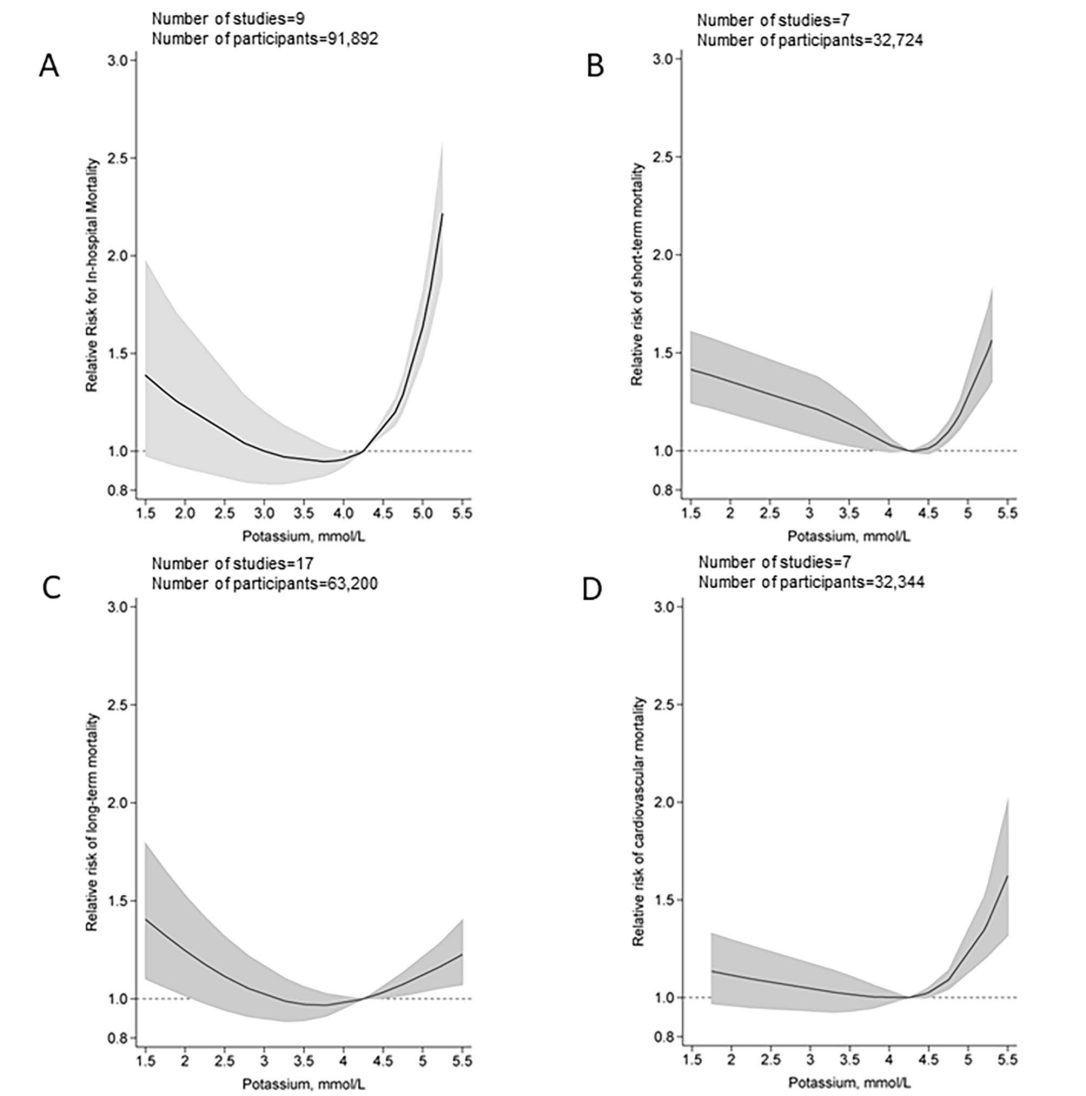
Dose-response analysis for association of blood potassium levels with all-cause in-hospital mortality risk (**A**); all-cause short-term mortality risk (**B**); all-cause long-term mortality risk (**C**) and cardiovascular mortality risk (**D**). Solid lines represent summary relative risks; shaded areas are the corresponding 95% confidence intervals

28 studies examined the association of hypokalemia with all-cause mortality among patients with CVD [13–15, 30–50, 52–54, 56], including nine studies of all-cause in-hospital mortality [13, 14, 33–35, 40, 48–50], eight of all-cause short-term mortality [38, 40, 43, 45, 49, 50, 52, 54] and 18 of all-cause long-term mortality [30–32, 35, 36, 38–42, 44, 46–48, 53, 54, 56]. Participants with hypokalemia had a significantly 65% higher risk of all-cause in-hospital mortality (RR:1.65,95CI%:1.22,2.25; *I*^2^ = 81.6, *P*_*heterogeneity*_<0.001) compared with those with normokalemia (Fig. 2; Figure 1 in Additional file 10). Similar positive associations with hypokalemia were also observed for all-cause long-term mortality (RR:1.35,95CI%:1.17,1.55; *I*^2^ = 83.2, *P*_*heterogeneity*_<0.001; Figure 7 in Additional file 16). Results for CVD subtypes presented consistent direction of the main associations (Figures 2–3, 5–6, 8–9 in Additional files 11–12, 14–15, 17–18).

Six studies were included in the analysis of hypokalemia with cardiovascular mortality [4, 32, 35, 39, 44, 56]. The pooled results found a significant risk increase of cardiovascular mortality by 55% in the participants with hypokalemia than those with normokalemia (RR:1.55,95CI%:1.18,2.03; *I*^2^ = 74.4, *P*_*heterogeneity*_=0.002; Figure 10 in Additional file 19). Subgroup analyses based on the study and participant characteristics did not appreciably alter the association of hypokalemia with mortality (all *P*_*meta−regression*_>0.10) (Tables 5–6 in Additional files 6–7).

### Association of hyperkalemia with all-cause and cardiovascular mortality

The association between hyperkalemia and all-cause mortality was reported in 27 studies [13–15, 30–39, 41–50, 52–55], including eight studies of all-cause in-hospital mortality [13, 14, 33–35, 48–50], seven of all-cause short-term mortality [38, 43, 45, 49, 50, 52, 54] and 17 of all-cause long-term mortality [30–32, 35, 36, 38, 39, 41, 42, 44, 46–48, 53–55]. The pooled results showed that hyperkalemia was significantly associated with 3.0-fold elevated risk of all-cause in-hospital mortality (RR:2.78,95CI%:1.92,4.03; *I*^2^ = 87.3, *P*_*heterogeneity*_<0.001; Fig. 2; and Figure 11 in Additional file 20). The positive associations with hyperkalemia were also statistically significant for all-cause short-term mortality (RR:1.80, 95CI%:1.44,2.27; *I*^2^ = 78.9, *P*_*heterogeneity*_<0.001) and all-cause long-term mortality (RR:1.33, 95CI%: 1.19,1.48; *I*^2^ = 68.8, *P*_*heterogeneity*_<0.001; Figures 13, 16 in Additional files 22, 25). Such associations persisted among participants with individual CVD over different time periods (Figures 12, 14–15, 17–18 in Additional files 21, 23–24, 26–27).

The analysis of six studies on cardiovascular mortality showed a significantly increased risk of mortality when comparing hyperkalemia with normokalemia (RR:1.19, 95CI%:1.04,1.36; *I*^2^ = 0.0, *P*_*heterogeneity*_=0.47; Figure 19 in Additional file 28) [4, 32, 35, 39, 44, 55]. Results of subgroup analyses and meta-regression showed that most predefined strata may not explain the heterogeneity (*P*_*meta−regression*_>0.10) with the exception of study design for all-cause short-term mortality (*P* = 0.004) and age for all-cause long-term mortality (*P* = 0.004) (Tables 7–8 in Additional files 8–9).

In a secondary analysis based on hypokalemia together with normokalemia as a reference category from 25 studies [4, 13–15, 30–37, 39, 41, 42, 44–50, 52–54], the associations of hyperkalemia with mortality were attenuated, with a pooled RR of 2.21 (95CI%:1.60,3.06) for all-cause in-hospital mortality, 1.46 (95CI%:1.25,1.71) for all-cause short-term mortality, 1.23 (95CI%:1.09,1.39) for all-cause long-term mortality and 1.13 (95CI%:1.00,1.27) for cardiovascular mortality (Figures 20–23 in Additional files 29–32).

### Dose-response association between blood potassium levels and risk of mortality

In the dose-response analysis, the association between blood potassium levels and all-cause in-hospital mortality appeared to be nonlinear (*P*_*non−linearity*_<0.001; Fig. 3; Figure 25 in Additional file 34). The risk of all-cause in-hospital mortality decreased with increasing potassium levels up to 3.9–4.2 mmol/L. When the potassium level exceeded 4.2 mmol/L, an increased risk of mortality tended to be evident with the magnitude of risk increase being stronger for potassium levels beyond 4.5 mmol. Even for the participants with a potassium level of 5.0 (equivalent to upper limit of normal range), a 65% higher risk of all-cause in-hospital mortality (RR:1.65, 95CI%:1.48,1.86) was observed compared with those with a potassium level in 4.2 mmol/L. Similarly, a U-shaped association was observed for all-cause short-term mortality across the potassium levels (*P*_*non−linearity*_<0.001), with the lowest risk associated with potassium level of around 4.2 (4.2–4.5) mmol/L. The risk of mortality tended to increase linearly for blood potassium levels ≥ 4.5 mmol/L (RR per mmol/L increment: 1.05, 95%CI:1.04,1.05). For all-cause long-term and cardiovascular mortality, the dose-response analysis also suggested a U-shaped association between potassium levels and mortality with a nadir of 4.2 mmol/L (*P*_*non−linearity*_<0.001).

### Sensitivity analysis and publication bias

The sensitivity analyses indicated that the exclusion of any single study from the analyses did not materially change the results except for the association of hypokalemia with short-term mortality (Figures 26–27 in Additional files 35–36). More studies with a larger sample size are needed to confirm our findings for short-term mortality. Additionally, the results remained almost unchanged when we restricted the analyses to studies that used potassium levels < 3.5 mmol/L to define hypokalemia and > 5.0 mmol/L to define hyperkalemia, respectively. (Figure 24 in Additional file 33). Visual examination of the funnel plot exhibited symmetry (Figure 28 in Additional file 37) and no indication of significant publication bias was found after performing Begg’s and Egger’s test(*P* > 0.05).

## Discussion

The meta-analysis showed that both hypokalemia and hyperkalemia were significantly associated with higher risk of all-cause and cardiovascular mortality, irrespective of the length of follow-up and CVD subtypes. The positive association of hyperkalemia with mortality might be underestimated when some studies treated hypokalemia together with normokalemia as the reference group. The dose-response analysis showed a U-shaped relationship between the potassium level and mortality, with the minimal risk at potassium of around 4.2 mmol/L. These findings suggest the detrimental impacts of abnormal potassium level on the progression of CVD, which raises the possibility of developing clinical practice guidelines for the CVD management.

Accumulating evidence suggests that dyskalemia could strongly lead to changes of the electrophysiological actions of cardiovascular system, which in turn subsequently contributes to the occurrence of cardiac arrhythmias and death [3, 4]. Extensive epidemiological studies regarding patients with established CVD have been conducted to investigate the association of hypokalemia and hyperkalemia with risk of mortality with inconsistent results. Among 44,799 hypertensive patients from the Danish National Registries, as compared with participants with normal potassium levels, those with hypokalemia and hyperkalemia had an approximately 2.8-fold and 1.7-fold increased risk of all-cause mortality within three months, respectively [16]. Results of the United Kingdom Heart Failure Evaluation and Assessment of Risk Trial showed that every 1-SD decrease in levels of serum potassium was significantly associated with a 64% higher risk of CVD mortality in hypokalemic patients with HF [57]. Similar to these findings, the present meta-analysis suggested that both hypokalemia and hyperkalemia were positively associated with risk of mortality among patients with total and individual CVD. Moreover, our results also showed that these positive associations persisted in different follow-up periods. These findings were in agreement with those of the study by Shiyovich et al. [54], in which a significantly elevated risk of mortality was observed from 6 months to 5 years among MI patients with hyperkalemia than those with normokalemia during 8.1 years of follow-up. Cooper et al. found that hypokalemia was significantly associated with higher risk of long-term mortality at all time points, and hyperkalemia also tended to be associated with mortality of different periods [38]. Therefore, the maintenance of potassium homeostasis might be essential for preventing CVD progression and premature death.

Although incompletely understood, the biological mechanisms underlying the association between potassium abnormalities and unfavorable outcomes following CVD may involve in the impairment of myocardial contractile and relaxation [58]. Potassium disturbance has generally been thought to perturb resting membrane potential in cardiomyocytes, resulting in abnormal myocardial impulse generation and conduction, which has been implicated in the development of the cardiac arrhythmias and death [59, 60]. Using pig moderator band perfused by low-potassium solutions, Gettes et al. reported that hypokalemia shortened the effective refractory period of ventricular fibers and prolonged the effective refractory period of Purkinje fibers, which increased heterogeneity of myocardial excitability that leaded to ventricular arrhythmias [61]. In *vitro*, generation of Ca^2+^-dependent spontaneous activity and delayed afterdepolarizations increased via close functional pairing of the Na^+^-K^+^ ATPase and Na^+^-Ca^2+^ exchanger proteins in rat ventricular and atrial myocytes exposed to hypokalemia conditions [62]. Also, hyperkalemia could markedly lengthen the atrium-to-His bundle interval and impair the atrioventricular nodal conduction by increasing inward rectifier potassium current in the isolated heart of guinea pigs [63]. Other potential mechanisms through which dyskalemia may lead to the progression of CVD involve in promoting inflammation and oxidative stress [5, 6]. A low potassium concentration has been shown to increase macrophage adherence to the vascular wall and accelerate atherosclerotic plaque formation by inducing of the inflammatory response [64]. In monosodium urate crystals induced human THP1 cells models with low potassium levels, Pétrilli et al. reported increasing concentration of potassium could block NLRP3 inflammasome assembly, which further inhibited the maturation and release of proinflammatory cytokines interleukin-1β [65]. In rabbits fed either a control diet or a low-potassium diet for one or three weeks, the low-potassium diet resulted in reduction of the sensitivity to endothelium-dependent stimuli via increasing free radical generation and degrading nitric oxide [66]. More studies are needed to elucidate the underling mechanistic implication of potassium abnormalities in relation to cardiovascular outcomes.

The potassium metabolism has been substantially altered for most patients with CVD given a complex interplay of poor nutritional status, routinely prescribed medications and renal insufficiency. Compared to general population, patients with CVD may be particularly vulnerable to the adverse outcomes associated with abnormal potassium levels [7, 8, 67, 68]. Although normokalemia has traditionally been considered as 3.5–5.5 mmol/L for the general population, no consensus recommendations have been issued regarding potassium levels to optimize CVD progression. American Council on Potassium in Clinical Practice recommended that maintaining potassium level ≥ 4.0 mmol/L is critical in patients with asymptomatic HTN or cardiac arrhythmias [69], whereas other studies argued that potassium levels over 4.5 mmol/L could minimize the risk of mortality in patients with MI and HF [70]. In addition, evidence from patients with CVD indicate that a potassium concentration, even in the range of 3.5–3.9 mmol/L, or above 4.9 mmol/L might be also associated with increased risk of mortality [15, 16, 40, 45]. Concern has been raised about generalizability of the reference range of general population to higher cardiovascular risk populations. Our results showed that potassium level around 4.2 mmol/L (3.9–4.5 mmol/L) was significantly associated with the lowest risk of mortality, regardless of CVD subtypes. This result was further supported by our secondary analysis that the inclusion of hypokalemic participants in the normal reference group would underestimate the true association of hyperkalemia with mortality, which may be explainable by the harmful impacts of hypokalemia ignored. Compared with hyperkalemia, hypokalemia may be often asymptomatic and tend to be potentially under-recognized in most patients [71, 72]. However, numerous studies revealed that hypokalemia had been found to be common in patients with CVD similar to hyperkalemia and even a mild reduction of potassium level (3.5–3.9 mmol/L) was associated with poor cardiovascular health [72, 73], indicating the importance of avoiding potential detrimental impacts of hypokalemia for patients with cardiovascular risk. Therefore, our findings have far-reaching implications for guiding patients with CVD towards maintaining optimal potassium level.

Several explanations were present for the heterogeneity observed in our study although the results of a series of sensitivity analyses suggested that our findings were robust. First, in case many studies with different characteristics were combined, the heterogeneity was inevitable and even small differences in RRs from included studies may lead to heterogeneity in the data [74]. Second, we have managed the included studies according to the PECOs (Population, Exposure, Comparator, Outcome, and Study) statement. The heterogeneity found in our study may be statistical heterogeneity [75]. Third, performing meta-analysis was appropriate in case that individual studies were in the same direction [76]. Of 29 studies included in the present analyses of hypokalemia, seven studies reported positive associations. Of 28 studies included in the present analyses of hyperkalemia, most studies reported positive associations except one study. The heterogeneity in the meta-analysis may be attributed to the difference in the magnitude (weak, moderate, or strong) of the effect estimates instead of the direction (positive or inverse) of the associations. Therefore, it was essential to differentiate the situations where the heterogeneity was driven by the magnitude rather than direction of the associations.

Several limitations of the current study deserve attention. First, although most included studies accounted for varying confounding factors for CVD, unmeasured or residual confounders (different methodologies for potassium assessment, lack of specific drug data that would have an impact on potassium and the outcome, e.g. potassium binders or diuretics or both, and CKD severity) may potentially impact the observed association in the study. Second, blood potassium levels in most included studies were measured only at baseline, and changes of potassium levels over time could lead to potential misclassification errors ascribed to within-person variation. However, potassium levels were found to have a low intra-individual variability coefficient (5%) based on repeated measurements in the previous cohort studies, indicating that potassium levels appeared to be relatively stable over time [77, 78]. In addition, not all studies report multiple measurements of potassium at baseline. Although the results of subgroup analyses showed no significant differences in RR stratified by potassium measurement times, the potential impact of variation in potassium assessment times should not be ignored. Third, measurement of potassium level performed from different blood sample sources (i.e., plasma and serum) could affect our results. However, previous studies suggested that the difference in the serum and plasma values may generally be of minimal clinical significance [79]. In addition, our subgroup analysis by the type of blood sample did not appreciably alter the strengths of associations. Fourth, the blood potassium levels for hypokalemia and hyperkalemia varied across different studies. Although we managed the blood potassium levels for hypokalemia and hyperkalemia for the included studies, and several sensitivity analyses only including studies that defined hypokalemia and hyperkalemia as blood potassium levels < 3.5 mmol/L and > 5.0 mmol/L, respectively, yielded the similar results, we could not exclude the possibility that the associations may be potentially affected by the heterogeneity in the potassium levels for hypokalemia and hyperkalemia among included studies. Further studies based on recommended clinical hypokalemia and hyperkalemia are needed to confirm our findings. Fifth, cardiovascular medications may have a potential effect on the association between potassium levels and mortality. Although our subgroup analysis suggested that the observed associations did not differ significantly by whether adjustment for the use of medications, the possibility that these drugs might partly mediate the potassium level-mortality association could not be fully excluded. Sixth, although the consistencies between these findings indicated that the associations were robust and combining studies with different participant characteristics did not bias the present results, the heterogeneity was still an apparent issue across most outcomes in our study. More research exploring the association between dyskalemia and mortality warrants further study. Finally, although the absence of publication bias was detected in our study, the possibility of publication bias by exclusion of gray literature and complicated unpublished data could be completely ruled out.

## Conclusion

For patients with CVD, both hypokalemia and hyperkalemia were associated with the increased risk of mortality, with the lowest risk observed with a blood potassium level of around 4.2 mmol/L. Furthermore, our results suggest that the possibility of harmful impacts of hypokalemia on cardiovascular health should also attract much attention. Further studies are warranted to ascertain the potential role of abnormal potassium levels in CVD progression and to elucidate the underlying biological mechanisms.

### Electronic supplementary material

Below is the link to the electronic supplementary material.

Supplementary Material 1

Supplementary Material 2

## Data Availability

The data that support the findings of this study are available from the corresponding author upon reasonable request.

## Abbreviations

CIs: confidence intervals
CKD: chronic kidney disease
CVD: cardiovascular disease
HF: heart failure
MI: myocardial infarction
RRs: relative risks
