# Supplementary material for: Potassium levels and the risk of all-cause and cardiovascular mortality among patients with cardiovascular diseases: a meta-analysis of cohort studies
Source: Nutr J. 2024 Jan 10;23:8. doi: 10.1186/s12937-023-00888-z (PMC10777575; doi:10.1186/s12937-023-00888-z)
Supplement: Supplementary file 2 — Supplementary Material 2 [file 12937_2023_888_MOESM2_ESM.docx]

Supplementary Tables

**Additional file 2: Table 1.** Search strategy

| 1. “potassium” |
| --- |
| 1. “hypokalaemia” |
| 1. “hypokalemia” |
| 1. “hyperkalaemia” |
| 1. “hyperkalemia” |
| 1. (1 OR 2 OR 3 OR 4 OR 5) |
| 1. “blood” |
| 1. “serum” |
| 1. “plasma” |
| 1. “circulating” |
| 1. (7 OR 8 OR 9 OR 10) |
| 1. “cardiovascular disease” |
| 1. “cardiovascular event” |
| 1. “cardiocerebrovascular disease” |
| 1. “coronary artery disease” |
| 1. “coronary heart disease” |
| 1. “ischemic heart disease” |
| 1. “myocardial infarction” |
| 1. “stroke” |
| 1. “angina” |
| 1. “heart failure” |
| 1. “cardiac failure” |
| 1. “revascularization” |
| 1. “angioplasty” |
| 1. “coronary artery bypass” |
| 1. “PCI” |
| 1. “PTCA” |
| 1. “CABG” |
| 1. (11 OR 12 OR 13 OR 14 OR 15 OR 16 OR 17 OR 18 OR 19 OR 20 OR 21 OR 22 OR 23 OR 24 OR 25 OR 26 OR 27 OR 28) |
| 1. “mortality” |
| 1. “death” |
| 1. “fatal” |
| 1. “survival” |
| 1. (30 OR 31 OR 32 OR 33) |
| 1. 6 AND 11 AND 29 AND 34 |

**Additional file 3: Table 2.** List of excluded articles after full-text review (n=128)

| No. | First author's name | Year | Title | Reasons for exclusion |
| --- | --- | --- | --- | --- |
| 1 | Mette Aldahl | 2021 | Short-term prognosis of normalising serum potassium following an episode of hypokalaemia in patients with chronic heart failure | Duplicate population |
| 2 | Gianluigi Savarese | 2019 | Incidence, Predictors, and Outcome Associations of Dyskalemia in Heart Failure With Preserved, Mid-Range, and Reduced Ejection Fraction | Duplicate population |
| 3 | Maria Lukács Krogager | 2015 | Short-term mortality risk of serum potassium levels in acute heart failure following myocardial infarction | Duplicate population |
| 4 | A Brent Alper | 2009 | A propensity-matched study of low serum potassium and mortality in older adults with chronic heart failure | Duplicate population |
| 5 | Andrea Lopez-López | 2023 | Impact of Hyperkalemia in Heart Failure and Reduced Ejection Fraction: A Retrospective Study | Not relevant exposure |
| 6 | Francesca Musella | 2023 | Patient profiles in heart failure with reduced ejection fraction: Prevalence, characteristics,treatments and outcomes in a real-world heartfailure population | Not relevant exposure |
| 7 | Jingmin Zhou | 2023 | Clinical outcomes by serum potassium levels for patients hospitalized for heart failure: Secondary analysis of data from the China National Heart Failure Registry | Not relevant exposure |
| 8 | Mu'taz Dreidi | 2023 | Electrolyte Imbalance Among Patients With and With No ST-Elevation Myocardial Infarction: A Cohort Study | Not relevant exposure |
| 9 | Sofie Solhøj Jønsson | 2023 | Short-term prognosis of changes in plasma potassium following an episode of hyperkalaemia in patients with chronic heart failure | Not relevant exposure |
| 10 | Yang Zhang | 2023 | Development of a prediction model for the risk of 30-day unplanned readmission in older patients with heart failure: A multicenter retrospective study | Not relevant exposure |
| 11 | Zehao Lin | 2023 | Assessing potassium levels in critically ill patients with heart failure: application of a group-based trajectory model | Not relevant exposure |
| 12 | Zubaid Rafique | 2023 | Hyperkalemia in acute heart failure: Short term outcomes from the EAHFE registry | Not relevant exposure |
| 13 | José Luis Gorriz | 2022 | Long-term mortality and trajectory of potassium measurements following an episode of acute severe hyperkalaemia | Not relevant exposure |
| 14 | Xi-Ling Zhang | 2021 | Potassium variability during hospitalization and outcomes after discharge in patients with acute myocardial infarction | Not relevant exposure |
| 15 | Yaping Ren | 2021 | Relationship between Admission Electrolyte Level and Short-term Prognosis of Patients with Acute ST-segment Elevation Myocardial Infarction after Percutaneous Coronary Intervention | Not relevant exposure |
| 16 | Maurizio Volterrani | 2020 | Effects of hyperkalaemia and non-adherence to renin-angiotensin-aldosterone system inhibitor therapy in patients with heart failure in Italy: a propensity-matched study | Not relevant exposure |
| 17 | Adnan Kaya | 2019 | Effect of Dynamic Potassium Change on In-Hospital Mortality, Ventricular Arrhythmias, and Long-Term Mortality in STEMI | Not relevant exposure |
| 18 | Cecilia Linde | 2019 | Real-World Associations of Renin-Angiotensin-Aldosterone System Inhibitor Dose, Hyperkalemia, and Adverse Clinical Outcomes in a Cohort of Patients With New-Onset Chronic Kidney Disease or Heart Failure in the United Kingdom | Not relevant exposure |
| 19 | Joost C Beusekamp | 2019 | Hyperkalemia and Treatment With RAAS Inhibitors During Acute Heart Failure Hospitalizations and Their Association With Mortality | Not relevant exposure |
| 20 | Kunihiro Matsushita | 2019 | Dyskalemia, its patterns, and prognosis among patients with incident heart failure: A nationwide study of US veterans | Not relevant exposure |
| 21 | Ygal Plakht | 2019 | The association of concomitant serum potassium and glucose levels and in-hospital mortality in patients with acute myocardial infarction (AMI). Soroka acute myocardial infarction II (SAMI-II) project | Not relevant exposure |
| 22 | Christoffer Polcwiartek | 2018 | Prognostic role of serum sodium levels across different serum potassium levels in heart failure patients: A Danish register-based cohort study | Not relevant exposure |
| 23 | Garrett S Bowen | 2018 | A Multivariable Prediction Model for Mortality in Individuals Admitted for Heart Failure | Not relevant exposure |
| 24 | Fardous Charles Abeya | 2018 | Incidence and predictors of 6 months mortality after an acute heart failure event in rural Uganda: The Mbarara Heart Failure Registry (MAHFER) | Not relevant exposure |
| 25 | Reimar Wernich Thomsen | 2018 | Elevated Potassium Levels in Patients With Congestive Heart Failure: Occurrence, Risk Factors, and Clinical Outcomes: A Danish Population-Based Cohort Study | Not relevant exposure |
| 26 | Anan Younis | 2017 | Elevated Admission Potassium Levels and 1-Year and 10-Year Mortality Among Patients With Heart Failure | Not relevant exposure |
| 27 | Jasper Tromp | 2017 | Serum Potassium Levels and Outcome in Acute Heart Failure (Data from the PROTECT and COACH Trials) | Not relevant exposure |
| 28 | Anna Grodzinsky | 2016 | Prevalence and Prognosis of Hyperkalemia in Patients with Acute Myocardial Infarction | Not relevant exposure |
| 29 | Yuan-Lan Huang | 2016 | Lower mean corpuscular hemoglobin concentration is associated with poorer outcomes in intensive care unit admitted patients with acute myocardial infarction | Not relevant exposure |
| 30 | John E Madias | 2000 | Admission serum potassium in patients with acute myocardial infarction: its correlates and value as a determinant of in-hospital outcome | Not relevant exposure |
| 31 | Kai Zhao | 2023 | Associations between serum electrolyte and short-term outcomes in patients with acute decompensated heart failure | Not relevant exposure |
| 32 | Pascal R D Clephas | 2023 | Serum potassium level and mineralocorticoid receptor antagonist dose in a large cohort of chronic heart failure patients | Not relevant outcome |
| 33 | Anxin Wang | 2022 | Lower Serum Potassium Levels at Admission are Associated with the Risk of Recurrent Stroke in Patients with Acute Ischemic Stroke or Transient Ischemic Attack | Not relevant outcome |
| 34 | Eskinder Tafesse | 2022 | Serum potassium as a predictor of adverse clinical outcomes in patients with increasing comorbidity burden | Not relevant outcome |
| 35 | Yuzhao Lu | 2022 | The association between serum glucose to potassium ratio on admission and short-term mortality in ischemic stroke patients | Not relevant outcome |
| 36 | Miroslava Valentova | 2020 | Hypokalaemia and outcomes in older patients hospitalized for heart failure | Not relevant outcome |
| 37 | Akshay S Desai | 2018 | Incident Hyperkalemia, Hypokalemia, and Clinical Outcomes During Spironolactone Treatment of Heart Failure With Preserved Ejection Fraction: Analysis of the TOPCAT Trial | Not relevant outcome |
| 38 | Joost C Beusekamp | 2018 | Potassium and the use of renin-angiotensin-aldosterone system inhibitors in heart failure with reduced ejection fraction: data from BIOSTAT-CHF | Not relevant outcome |
| 39 | Taiki Nishihara | 2018 | Serum Potassium and Cardiovascular Events in Heart Failure With Preserved Left Ventricular Ejection Fraction Patients. | Not relevant outcome |
| 40 | Arash Nayeri | 2017 | Temporal Pattern and Prognostic Significance of Hypokalemia in Patients Undergoing Targeted Temperature Management Following Cardiac Arrest | Not relevant outcome |
| 41 | Sadiya S Khan | 2015 | Changes in serum potassium levels during hospitalization in patients with worsening heart failure and reduced ejection fraction (from the EVEREST trial) | Not relevant outcome |
| 42 | Vishal Chauhan | 2015 | Facility variation and predictors of serum potassium monitoring after initiation of a mineralocorticoid receptor antagonist in patients with heart failure | Not relevant outcome |
| 43 | Hiddo J Lambers Heerspink | 2014 | The effect of ramipril and telmisartan on serum potassium and its association with cardiovascular and renal events: results from the ONTARGET trial | Not relevant outcome |
| 44 | Jianling Su | 2012 | Additional predictive value of serum potassium to Thrombolysis In Myocardial Infarction risk score for early malignant ventricular arrhythmias in patients with acute myocardial infarction | Not relevant outcome |
| 45 | Stefano Muzzarelli | 2012 | Frequency and predictors of hyperkalemia in patients ≥60 years of age with heart failure undergoing intense medical therapy | Not relevant outcome |
| 46 | Akshay S Desai | 2007 | Incidence and predictors of hyperkalemia in patients with heart failure: an analysis of the CHARM Program | Not relevant outcome |
| 47 | J F E Mann | 2005 | Serum potassium, cardiovascular risk, and effects of an ACE inhibitor: results of the HOPE study | Not relevant outcome |
| 48 | Constança S Cruz | 2003 | Hyperkalaemia in congestive heart failure patients using ACE inhibitors and spironolactone | Not relevant outcome |
| 49 | S E Gariballa | 1997 | Hypokalemia and potassium excretion in stroke patients | Not relevant outcome |
| 50 | J E Nordrehaug | 1985 | Hypokalemia, arrhythmias and early prognosis in acute myocardial infarction | Not relevant outcome |
| 51 | Ali AlSahow | 2023 | Prevalence and management of hyperkalemia in chronic kidney disease and heart failure patients in the Gulf Cooperation Council (GCC) | Not relevant population |
| 52 | Matthew Johnson | 2023 | Outcomes in patients with cardiometabolic disease who develop hyperkalemia while treated with a renin-angiotensin-aldosterone system inhibitor | Not relevant population |
| 53 | Xinwei Peng | 2023 | Serum Nutritional Biomarkers and All-Cause and Cause-Specific Mortality in U.S. Adults with Metabolic Syndrome: The Results from National Health and Nutrition Examination Survey 2001–2006 | Not relevant population |
| 54 | Eskinder Tafesse | 2022 | Serum potassium as a predictor of adverse clinical outcomes in patients with increasing comorbidity burden | Not relevant population |
| 55 | Ryuichirou Makinouchi | 2022 | Severe hypokalemia in the emergency department: A retrospective, single-center study | Not relevant population |
| 56 | Tiantian Chu | 2022 | Association between preoperative hypokalemia and postoperative complications in elderly patients: a retrospective study | Not relevant population |
| 57 | Toshiaki Ohkuma | 2022 | Short-Term Changes in Serum Potassium and the Risk of Subsequent Vascular Events and Mortality: Results from a Randomized Controlled Trial of ACE Inhibitors | Not relevant population |
| 58 | Yuan Ma | 2022 | 24-Hour Urinary Sodium and Potassium Excretion and Cardiovascular Risk | Not relevant population |
| 59 | Shuai Liu | 2021 | Association of Serum Potassium Levels with Mortality and Cardiovascular Events: Findings from the Chinese Multi-provincial Cohort Study | Not relevant population |
| 60 | Ygal Plakht | 2021 | Potassium levels as a marker of imminent acute kidney injury among patients admitted with acute myocardial infarction. Soroka Acute Myocardial Infarction II (SAMI-II) Project | Not relevant population |
| 61 | Afrasyab Altaf | 2020 | Prognostic significance of serum potassium level for major adverse cardiac events and death in patients with coronary atherosclerotic disease | Not relevant population |
| 62 | Patrick Rossignol | 2020 | Cardiovascular risk associated with serum potassium in the context of mineralocorticoid receptor antagonist use in patients with heart failure and left ventricular dysfunction | Not relevant population |
| 63 | Lila Bouadma | 2019 | Influence of dyskalemia at admission and early dyskalemia correction on survival and cardiac events of critically ill patients | Not relevant population |
| 64 | Marco Trevisan | 2018 | Incidence, predictors and clinical management of hyperkalaemia in new users of  mineralocorticoid receptor antagonists | Not relevant population |
| 65 | Yan Chen | 2018 | Race, Serum Potassium, and Associations With ESRD and Mortality | Not relevant population |
| 66 | Allan J Collins | 2017 | Association of Serum Potassium with All-Cause Mortality in Patients with and without Heart Failure, Chronic Kidney Disease, and/or Diabetes | Not relevant population |
| 67 | Patrick Rossignol | 2017 | Impact of eplerenone on cardiovascular outcomes in heart failure patients with hypokalaemia | Not relevant population |
| 68 | Patrick H Pun | 2017 | Serum Potassium Levels and Risk of Sudden Cardiac Death Among Patients With Chronic Kidney Disease and Significant Coronary Artery Disease | Not relevant population |
| 69 | Yan Chen | 2016 | Serum Potassium, Mortality, and Kidney Outcomes in the Atherosclerosis Risk in Communities Study | Not relevant population |
| 70 | Helene Kildegaard Jensen | 2014 | Hypokalemia in acute medical patients: risk factors and prognosis | Not relevant population |
| 71 | Orly Vardeny | 2014 | Incidence, predictors, and outcomes related to hypo-and hyperkalemia in patients with severe heart failure treated with a mineralocorticoid receptor antagonist | Not relevant population |
| 72 | Patrick Rossignol | 2014 | Incidence, determinants, and prognostic significance of hyperkalemia and worsening renal function in patients with heart failure receiving the mineralocorticoid receptor antagonist eplerenone or placebo in addition to optimal medical therapy: results from the Eplerenone in Mild Patients Hospitalization and Survival Study in Heart Failure (EMPHASIS-HF) | Not relevant population |
| 73 | DR. P.KIRANMAI M.D | 2013 | Serial estimation of serum magnesium, calcium, sodium and potassium levels in myocardial infarction | Not relevant population |
| 74 | Jung Nam An | 2012 | Severe hyperkalemia requiring hospitalization: predictors of mortality | Not relevant population |
| 75 | Nishank Jain | 2012 | Predictors of hyperkalemia and death in patients with cardiac and renal disease. | Not relevant population |
| 76 | C. Barrett Bowling | 2010 | Hypokalemia and Outcomes in Patients With Chronic Heart Failure and Chronic Kidney Disease Findings From Propensity-Matched Studies | Not relevant population |
| 77 | Tung-Wai Auyeung | 2007 | Prognostic significance of admission electrolyte disturbances in older people: A retrospective cohort study | Not relevant population |
| 78 | D.M. Green | 2002 | Serum potassium level and dietary potassium intake as risk factors for stroke | Not relevant population |
| 79 | Susan R. Reuben | 1982 | The relationship between serum potassium and cardiac arrhythmias following cardiac infarction in patients aged over 65 years | Not relevant population |
| 80 | B Khan | 2023 | Serum Electrolyte Status of Patients with Acute Stroke Admitted in a Tertiary Care Hospital | Not relevant study design |
| 81 | Rocío Del Pilar Laymito-Quispe | 2022 | Prognostic implications of hypo and hyperkalaemia in acute heart failure with reduced ejection fraction. Analysis of cardiovascular mortality and hospital readmissions | Not relevant study design |
| 82 | Toshiaki Ohkuma | 2022 | Short-Term Changes in Serum Potassium and the Risk of Subsequent Vascular Events and Mortality: Results from a Randomized Controlled Trial of ACE Inhibitors | Not relevant study design |
| 83 | Christina Byrne | 2021 | Serum Potassium and Mortality in High-Risk Patients: SPRINT | Not relevant study design |
| 84 | Joseph B Muhlestein | 2021 | Frequency and clinical impact of hyperkalaemia within a large, modern, real-world heart failure population | Not relevant study design |
| 85 | Sijan Basnet | 2019 | Influence of abnormal potassium levels on mortality among hospitalized heart failure patients in the US: data from National Inpatient Sample | Not relevant study design |
| 86 | Khibar Salah | 2015 | Serum potassium decline during hospitalization for acute decompensated heart failure is a predictor of 6-month mortality, independent of N-terminal pro-B-type natriuretic peptide levels: An individual patient data analysis | Not relevant study design |
| 87 | Orly Vardeny | 2014 | Incidence, predictors, and outcomes related to hypo- and hyperkalemia in patients with severe heart failure treated with a mineralocorticoid receptor antagonist. | Not relevant study design |
| 88 | C Barrett Bowling | 2010 | Hypokalemia and outcomes in patients with chronic heart failure and chronic kidney disease: findings from propensity-matched studies | Not relevant study design |
| 89 | Bertram Pitt | 2008 | Serum potassium and clinical outcomes in the Eplerenone Post-Acute Myocardial Infarction Heart Failure Efficacy and Survival Study (EPHESUS) | Not relevant study design |
| 90 | J Fang | 2000 | Serum potassium and cardiovascular mortality | Not relevant study design |
| 91 | R J Solomon | 1981 | Importance of potassium in patients with acute myocardial infarction | Not relevant study design |
| 92 | Daniel Murphy | 2022 | Hyperkalaemia in Heart Failure: Consequences for Outcome and Sequencing of Therapy | Review |
| 93 | Camila Cristiane Toledo | 2021 | Serum potassium levels provide prognostic information in symptomatic heart failure beyond traditional clinical variables | Review |
| 94 | Dimitrios Sfairopoulos | 2021 | Serum potassium and heart failure: association, causation, and clinical implications. | Review |
| 95 | José Silva-Cardoso | 2021 | Management of RAASi-associated hyperkalemia in patients with cardiovascular disease | Review |
| 96 | Faiez Zannad | 2019 | Potassium binders for the prevention of hyperkalaemia in heart failure patients: implementation issues and future developments | Review |
| 97 | Jay Ian Lakkis | 2018 | Hyperkalemia in the Hypertensive Patient | Review |
| 98 | Khibar Salah | 2015 | Serum potassium decline during hospitalization for acute decompensated heart failure is a predictor of 6-month mortality, independent of N-terminal pro-B-type natriuretic peptide levels: An individual patient data analysis | Review |
| 99 | Jerry M Buysse | 2012 | PEARL-HF: prevention of hyperkalemia in patients with heart failure using a novel polymeric potassium binder, RLY5016 | Review |
| 100 | Bill D Gogas | 2011 | Instantaneous electrocardiographic changes and transient sinus rhythm restoration in severe hyperkalaemia | Review |
| 101 | Akshay S Desai | 2009 | Hyperkalemia in patients with heart failure: incidence, prevalence, and management | Review |
| 102 | G Quick | 1994 | Prolonged asystolic hyperkalemic cardiac arrest with no neurologic sequelae | Review |
| 103 | M Packer | 1990 | Potential role of potassium as a determinant of morbidity and mortality in patients with systemic hypertension and congestive heart failure | Review |
| 104 | Henry J Dargie | 1987 | Relation of arrhythmias and electrolyte abnormalities to survival in patients with severe chronic heart failure | Review |
| 105 | T Dyckner | 1987 | Potassium/magnesium depletion in patients with cardiovascular disease | Review |
| 106 | McEwan P | 2020 | The relationship between uration of heart failure, serum potassium concentration and  adverse clinical outcomes | Abstract |
| 107 | Steffiany | 2019 | Association of serum sodium, potassium and chloride value on admission with st elevation myocardial infarcts patient's global registry of acute coronary event at Tarakan Regional Public Hospital, North Kalimantan | Abstract |
| 108 | Ozturk B Begum | 2018 | Potassium in heart failure with preserved ejection fraction-association with outcome and clinical parameters | Abstract |
| 109 | Oztürk, B | 2018 | Serum potassium levels and outcome in patients with heart failure and preserved ejection fraction | Abstract |
| 110 | Qin L | 2017 | Association between serum potassium and clinical outcomes in UK patients with heart failure | Abstract |
| 111 | Cooper, Lauren Beth | 2017 | Association between serum potassium level and outcomes in heart failure with reduced ejection fraction: a cohort study from the swedish heart failure registry | Abstract |
| 112 | Charles A Herzog | 2016 | Association of potassium values in hospitalized patients with mortality: Data from a metropolitan safetynet hospital | Abstract |
| 113 | Rivera Juarez, A. R | 2015 | Severe potassium disorders in patients with chronic heart failure admitted to a tertiary hospital: Clinical characterization, common causes and prognostic value | Abstract |
| 114 | Yuki Kakefuda | 2013 | Short-and long-term prognostic value of serum potassium level after percutaneous coronary intervention for ST-segment elevation myocardial infarction | Abstract |
| 115 | Goran Koracevic | 2012 | Serum potassium shows U-shapped curve for in-hospital mortality in 1063 heart failure patients | Abstract |
| 116 | Paul D. Loprinzi | 2009 | Serum potassium on mortality risk among a national sample of cardiovascular disease patients: Considerations by physical activity | Abstract |
| 117 | Rajesh G. Shenava | 2008 | Low serum potassium levels and increased mortality in older adults with chronic heart failure | Abstract |
| 118 | Joost C. Beusekamp | 2020 | Potassium abnormalities in patients with heart failure from 11 Asian regions: Insights from the ASIAN-HF registry | Letter/Editorial/Comment |
| 119 | Lauren B Cooper | 2019 | Potassium abnormalities across the spectrum of heart failure. JACC: Heart Failure | Letter/Editorial/Comment |
| 120 | Tadao Aikawa | 2019 | Serum potassium and glucose levels, and mortality in acute myocardial infarction; fact or myth? | Letter/Editorial/Comment |
| 121 | Bertram Pitt | 2018 | Relation of Serum Potassium to Cardiovascular Events in Patients With Heart Failure and Preserved Ejection Fraction: “Mind the Gap” | Letter/Editorial/Comment |
| 122 | Fotios Barkas | 2018 | Serum Potassium Levels and Mortality in Acute Myocardial Infarction: Myth or Fact? | Letter/Editorial/Comment |
| 123 | Paul D Loprinzi | 2018 | Effect of Serum Potassium on Residual-specific Mortality: Interaction Evaluation by Physical Activity | Letter/Editorial/Comment |
| 124 | Bertram Pitt | 2017 | Serum potassium in patients with chronic heart failure: once we make a U-turn where should we go? | Letter/Editorial/Comment |
| 125 | Levent Cerit | 2016 | Admission serum potassium level is associated with in-hospital and long-term mortality in ST-elevation myocardial infarction | Letter/Editorial/Comment |
| 126 | Andreas Perren | 2012 | Potassium levels after acute myocardial infarction | Letter/Editorial/Comment |
| 127 | Benjamin M Scirica | 2012 | Potassium concentration and repletion in patients with acute myocardial infarction | Letter/Editorial/Comment |
| 128 | R Brooks Robey | 2012 | Potassium levels after acute myocardial infarction | Letter/Editorial/Comment |

**Additional file 4: Table 3.** General characteristics of included cohort studies (n=31)

| **Author,**  **publication year,**  **country** | **Design** | **Baseline survey year** | **Follow**  **up period**  **(year/**  **month)** | **Patients** | **Men (%)** | **Type of blood sample** | | **Exposure category** | **Measurement times** | **Outcome**  **(cases)** | **Outcome ascertainment**  **method^¶^** | **Adjustment for confounding factors** | **Study quality*** |
| --- | --- | --- | --- | --- | --- | --- | --- | --- | --- | --- | --- | --- | --- |
| Huang et al, 2023, China | RCS | 2008-2017 | 2.63 y | 2,621 HF aged 58.0 y | 71.6 | Serum | **<3.5**  **3.5-5.5**  **>5.5** | | **NR** | Long-term mortality  (1,076) | Medical records | Age, gender, NYHA class, HTN, renal dysfunction, ACEI/ARBs | High |
| Lombardi et al, 2022, Italy | RCS | 2003-2019 | 1.0 y | 926 HF aged 70.8 y | 70.4 | Serum | **<3.5**  **3.5-5.0**  **≥5.0** | | **Once** | Long-term mortality  (135) | Medical records | Age, gender, SBP, eGFR LVEF, hemoglobin, HF, serum potassium at baseline, ACEI/ARBs, MRA | High |
| Miura et al, 2022, Japan | RCS | 2006-2017 | 2.0 y | 3,398 HF aged 74.0 y | 60.0 | Serum | **<3.5**  **3.5-5.0**  **≥5.0** | | **Once** | Long-term mortality  (543) | Medical records | Age, eGFR, NYHA class III/IV, COPD, IHD, cachexia, combination medical therapy, loop diuretics, and thiazide diuretics | HIgh |
| Perez et al, 2022, Spain | PCS | 2013-2014 | 1.0 y | 1,779  HF aged 72.0 y | 59.0 | Serum | **<3.5**  **3.5-5.0**  **>5.0** | | **NR** | In-hospital mortality  (69) | Death registries | Age, BP, eGFR, hemoglobin, natremia, NT-ProBNP, NYHA class, Charlson and Barthel index, HF and PAD | High |
| Zhang et al, 2022, USA | RCS | 2014-2015 | NA | 8,731  MI aged 66.7 y | 63.2 | Serum | **<3.5**  **3.5-4.5**  **4.5-5.5**  **≥5.5** | | **Multiple** | In-hospital mortality  (836) | Death registries | Age, gender, AHF, acute kidney failure, AMI type, CHF, CKD, ACEI/ARBs, diuretics, insulin treatment, and potassium supplementation | High |
| Ferreira et al, 2021, multi-countries | RCT | 2014-2016 | 2.92 y  (median) | 4,796  HF aged 73.0 y | 48.0 | Serum | **<4.0**  **4.0-5.0**  **≥5.0** | | **Multiple** | Long-term mortality (691),  CVD mortality  (416) | Medical records | Age, gender, race, region, BMI, blood urea nitrogen, HR, eGFR, NT-ProBNP, SBP, LVEF, NYHA class, AF, DM, HTN, ischemic cardiomyopathy, prior HF hospitalization, prior stroke, diuretics, β-blockers, MRA, and treatment group allocation | High |
| Maggioni et al, 2021, Italy | PCS | 2014 | 1.0 y | 7,589  HF aged 70.2 y | 73.2 | Serum | **<3.5**  **3.5-4.9**  **5.0-5.4**  **≥5.5** | | **NR** | Long-term mortality  (558) | Medical records, death certificates, autopsy records, follow-up interviews | Age, gender, BMI, creatinine at entry>1.5, HR, SBP, clinical presentation at entry, worsening vs de novo HF, IHD, mitral regurgitation, PAD, peripheral congestion, renal dysfunction, stroke/TIA, prior hospitalization, ACEI/ARBs, MRA and β-blockers | High |
| Maggioni et al, 2021, Italy | PCS | 2014 | 1.0 y | 1,726  HF aged 74.7 y | 58.8 | Serum | **<3.5**  **3.5-4.9**  **5.0-5.4**  **≥5.5** | | **NR** | Long-term mortality  (429) | Medical records, death certificates, autopsy records, follow-up interviews | Age, gender, BMI, creatinine at entry>1.5, HR, SBP, clinical presentation at entry, worsening vs de novo HF, IHD, mitral regurgitation, PAD, peripheral congestion, renal dysfunction, stroke/TIA, prior hospitalization, ACEI/ARBs, MRA and β-blockers | High |
| Shiyovich et al, 2021, Israel | RCS | 2002-2012 | NA | 14,364  MI aged 68.1 y | 65.5 | Serum | **<3.8**  **3.8-4.0**  **4.0-4.4**  **4.4-4.9**  **≥4.9** | | **NR** | In-hospital mortality  (681) | Death registries | Age, AMI type, IHD, malignancy, PVD, renal diseases, supraventricular  arrhythmias and the results of other investigated blood tests | Medium |
| Cooper et al, 2020, Sweden | RCS | 2006-2012 | 2.0 y  (median) | 13,015  HF aged 71.0 y | 73.0 | Serum or plasma | **<3.5**  **3.5-5.0**  **>5.0** | | **NR** | Short-term mortality(1-m), long-term mortality(5-y) | Death registries | Age, gender, hemoglobin, HR, eGFR, SBP, EF, NYHA class, presence of defibrillator, pacemaker, AF, alcoholism, cardiac resynchronization therapy, DM, HF duration, HTN, liver disease, lung disease, malignant cancer, mental health problems, MI, musculoskeletal  problems, PAD, revascularization, severe bleeding, stroke, ACEI/ARBs, diuretics, digoxin, MRA, nitrates, β-blockers, oral anticoagulant, platelet  inhibitor and statin | High |
| Ferreira et al, 2020 multi-countries | RCT | 2009-2012 | 2.25 y  (median) | 8,223  HF aged 63.8 y | 78.0 | Serum | **≤3.5**  **3.6-4.0**  **4.1-4.9**  **5.0-5.4**  **≥5.5** | | **Multiple** | Long-term mortality (1,517), CVD mortality  (1,230) | Medical records | Age, gender, race, region, BMI, eGFR, HR, NT-ProBNP, SBP, LVEF, NYHA class, AF, DM, HTN, ischemic cardiomyopathy, prior HF hospitalization, prior MI, prior stroke, diuretics, digoxin, MRA, β-blockers, cardiac devices, and treatment group allocation | High |
| Valentova et al, 2020, USA | PCS | 1998-2001 | 3.3 y  (median) | 1,942  HF aged 75.0 y | 40.0 | Serum | **<4.0**  **4.4-5.0** | | **NR** | In-hospital mortality  (87), short-term mortality  (1-m,91), long-term mortality  (8.8-y, 1,223) | Medical records | Age, gender, race, admission from nursing home, LVEF, past medical history, clinical and laboratory findings, in-hospital events, discharge medications, hospital length of stay and hospital characteristics | High |
| Formiga et al, 2019, Spain | PCS | 2008-2017 | 1.0 y | 2,865  HF aged 83.0 y | 43.0 | Serum | **<3.5**  **3.5-5.5**  **>5.5** | | **NR** | Short-term mortality  (3-m,271), long-term mortality  (1-y,646) | Death registries | Age, BMI, creatinine, DBP, eGFR, hemoglobin, SBP, urea, sodium, LVEF, NYHA III-IV, etiology, Charlson and Barthel index, Pfeiffer index, dyslipidemia, ACEI/ARBs, ACEI/ARA, MRA and β-blockers | High |
| Linde et al, 2019, UK | RCS | 2006-2015 | 3.0 y  (mean) | 21,334  HF aged 73.0 y | 56.7 | Serum | **<3.5**  **3.5-4.0**  **4.0-4.5**  **4.5-5.0**  **5.0-5.5**  **5.5-6.0**  **≥6.0** | | **Multiple** | Long-term mortality | Medical records | Age, gender, smoking, BMI, eGFR, SBP, serum phosphorus, arrhythmia, chronic pulmonary disease, dementia, DM, malignancy, metastatic tumor, MI, stroke, PVD, peptic ulcer, rheumatic disease, ACEI/ARBs, bronchodilators, CCBs, diuretics, MRA, NSAIDs, Renin inhibitors, RAASi therapy, β-blockers and statins | High |
| Legrand et al, 2018, multi-countries | PCS | NA | 3.0 m | 15,954  HF aged 72.0 y | 56.5 | Serum | **<3.5**  **3.5-4.5**  **>4.5** | | **NR** | Short-term mortality | Medical records | Age, gender, region, eGFR, DBP, SBP, AF, CAD, DM, HF, hyperglycemia and hyponatremia | Medium |
| Núñez et al, 2018, Spain | PCS | 2008-2016 | 2.79 y  (median) | 2,164  HF aged 73.0 y | 50.4 | Serum | **<3.5**  **3.5-5.0**  **>5.0** | | **Multiple** | Long-term mortality (1,090) | Medical records | Gender, eGFR-time-varying <60 mL/min/1.73 m^2^, DM, and the use of potassium-modifying treatments | High |
| Xu et al, 2018, Sweden | PCS | 2006-2011 | 1.0 y | 4,861  MI aged 71.4 y | 65.0 | Plasma | **<3.5**  **3.5-4.0**  **4.0-4.5**  **4.5-5.0**  **>5.0** | | **NR** | Long-term mortality (718), CVD mortality  (295) | Death registries | Age, gender, AF, CABG, cancer, COPD, DM, HF, HTN, MI, non-STEMI, PCI, PVD, STEMI, stroke, and medication at discharge | High |
| Aldahl et al, 2017, Denmark | RCS | 1994-2012 | 3.0 m | 19,549  HF aged 75.8 y | 57.4 | Serum | **2.8-3.4**  **3.5-3.8**  **3.9-4.1**  **4.2-4.4**  **4.5-4.7**  **4.8-5.0**  **5.1-5.5**  **5.6-7.4** | | **NR** | Short-term mortality (1,384) | Death registries | Age, gender, creatinine, AMI, COPD, DM, ICD and relevant concomitant pharmacotherapy | High |
| Cheungpasitporn et al, 2017, USA | RCS | 2011-2013 | NA | 15,716  CVD aged 61.0 y | 53.0 | Serum | **<3.0**  **3.0-3.5**  **3.5-4.0**  **4.0-4.5**  **4.5-5.0**  **5.0-5.5**  **≥5.5** | | **NR** | In-hospital mortality  (317) | Medical records | Age, gender, race, eGFR, Charlson index, CAD, CHF, cirrhosis, COPD, DM, PVD, stroke, ACEI/ARBs, diuretics and potassium supplementation | High |
| Colombo et al, 2017, Germany | PCS | 2000-2008 | 6.1 y  (median) | 3,347  MI aged 59.9 y | 75.6 | Serum | **<3.5**  **3.5-4.0**  **4.0-4.5**  **4.5-5.0**  **>5.0** | | **NR** | Long-term mortality (481) | Death registries | Age, gender, smoking, CK-MB, angina pectoris, HTN, hyperlipidemia, stroke, status, revascularization treatment, ACEI/ARBs, antiplatelet agents, CCBs at discharge, diuretics at discharge, insulin at discharge, β-blockers and statins | High |
| Patel et al, 2017, multi-countries | PCS | 2004-2006 | 1.0 y | 6,515  MI aged 63.7 y | 65.0 | Serum | **<3.5**  **3.5-4.0**  **4.0-4.5**  **4.5-5.0**  **>5.0** | | **Multiple** | CVD mortality (1,746) | Medical records | Age, smoking, positive cardiac biomarkers, ST-segment deviation >0.5 mm, angina, CAD, DM, family history of CAD, HTN, hypercholesterolemia, history of congestive HF,  prior treatment with aspirin, creatinine clearance, and coronary angiography at the index hospitalization | High |
| Hoss et al,  2016, Israel | PCS | 2008 | 1.58 y | 6,073  HF aged 80.0 y | 50.0 | Serum | **≤3.5**  **3.5-4.0**  **4.0-5.0**  **5.0-5.5**  **5.5-6.0**  **≥6.0** | | **NR** | Long-term mortality (850) | Death registries | Age, gender, BMI, eGFR, hemoglobin, pulse, serum urea, serum sodium, hyperlipidemia,  HTN, ACEI/ARBs, furosemide, β-blockers, spironolactone, thiazide and aspirin | High |
| Keskin et al, 2016, Turkey | RCS | 2010-2012 | 1.98 y  (mean) | 3,760  MI aged 58.0 y | 81.0 | Serum | **<3.0**  **3.0-3.5**  **3.5-4.0**  **4.0-4.5**  **4.5-5.0**  **5.0-5.5**  **≥5.5** | | **Multiple** | In-hospital mortality (154),  long-term mortality (4-y, 191) | Medical records, follow-up interviews | Age, gender, first measurement during hospitalization of the following laboratory values, peak troponin level, presence of cardiogenic shock, acute respiratory failure on admission, procedures during, AKD during  hospitalization, comorbidities and medications during hospitalization | High |
| Ma et al, 2016, China | RCS | 2001-2004 | 1.0 m | 6,613  MI aged 62.7 y | 71.6 | Serum | **<3.5**  **3.5-4.0**  **4.0-4.5**  **4.5-5.0**  **≥5.0** | | **Multiple** | In-hospital mortality (279), short-term mortality (420) | Medical records, follow-up interviews | Age, gender, blood sugar, DBP, HR, hemoglobin, SBP, weight, onset-to-admission time, Killip  classification, electrocardiogram location, medical history and treatment | High |
| Shu et al, 2016, China | RCS | 2013-2015 | 6.0 m | 298  MI aged 59.2 y | 85.9 | Serum | **<3.5**  **3.5-4.0**  **4.0-4.5**  **4.5-5.0**  **≥5.0** | | **NR** | In-hospital mortality (10),  short-term mortality (16) | Medical records, follow-up interviews | Age, gender, BMI, eGFR, glucose, glycated hemoglobin, HDL, hemoglobin, LDL, phosphokinase, WBC, Killip classification, LVEF, DM and HTN | High |
| Shlomai et al, 2016, Israel**^†^** | PCS | 2010-2013 | 1.32 y | 1,277  MI aged 64.0 y | 78.0 | Serum | **3.5-3.9**  **3.91-4.18**  **4.19-4.45**  **4.62-5.2** | | **NR** | Short-term mortality (42), long-term mortality (88) | Death registries, medical records, follow-up interviews | Age, gender, eGFR, DM, HTN and dyslipidemia | High |
| Uluganyan et al, 2016, Turkey | RCS | 2011-2012 | 6.0 m | 611  MI aged 59.0 y | 86.0 | Serum | **<3.5**  **3.5-4.0**  **4.0-4.5**  **4.5-5.0**  **≥5.0** | | **NR** | In-hospital mortality (18),  short-term mortality (32) | Medical records, follow-up interviews | Age, gender, smoking, CK-MB, eGFR at admission, Killip classification, LVEF, anterior STEMI, CAD, DM, HTN, hyperlipidemia and medication before hospitalization | High |
| Choi et al, 2014, Korea | RCS | 2006-2009 | 3.58 y | 1,924  MI aged 64.0 y | 69.0 | Serum | **<3.5**  **3.5-4.0**  **4.0-4.5**  **4.5-5.0**  **≥5.0** | | **Multiple** | In-hospital mortality (83),  long-term mortality (3-y, 266) | Medical records, follow-up interviews | Age, gender, CK-MB, Killip classification, LVEF, co-morbidities, diagnosis, renal function, in-hospital procedure, and medical treatment during hospitalization | High |
| Shiyovich et al, 2014, Israel | PCS | 2002-2004 | 8.1 y  (median) | 2,434  MI aged 68.6 y | 66.5 | Serum | **<3.0**  **3.0-3.5**  **3.5-4.0**  **4.0-4.5**  **4.5-5.0**  **5.0-5.5**  **≥5.5** | | **Multiple** | Short-term mortality (6-m, 221), long-term mortality (10-y, 1,217) | Death registries | Age, alcohol, sodium, glucose, urea, hemoglobin, length of stay, intervention for AMI, left ventricular dysfunction, left ventricular hypertrophy, chronic renal failure, COPD, gastro-intestinal hemorrhage, malignant neoplasm, mitral regurgitation, obesity, pulmonary HTN, drug addiction, schizophrenia, psychosis and neurological disorders | High |
| Goyal et al, 2012, USA | RCS | 2000-2008 | NA | 38,689  MI aged 71.1 y | 63.7 | Serum | **<3.0**  **3.0-3.5**  **3.5-4.0**  **4.0-4.5**  **4.5-5.0**  **5.0-5.5**  **≥5.5** | | **Multiple** | In-hospital mortality  (2,265) | Death registries | Age, gender, race, comorbidities, first measurement during hospitalization of the following laboratory values, peak troponin level, AKD during hospitalization, CABG, PCI, presence of cardiogenic shock and acute respiratory failure on admission, procedures during hospitalization including cardiac catheterization, medications during hospitalization, number of serum potassium checks during hospitalization, length of hospital stay, and clustering by hospital site | High |
| Ahmed et al, 2010, USA &  Canada | RCS | 1991-1993 | 3.17 y | 2,177  HF aged 65.0 y | 79.0 | Serum | **4.0-4.9**  **5.0-5.5** | | **NR** | Long-term mortality (798),  CVD mortality  (625) | Medical records, follow-up interviews | Age, gender, race, BMI, chest radiograph findings, creatinine, eGFR, DBP, HR, SBP, EF, NYHA class, symptoms and signs of HF, duration of HF, primary cause of HF, current angina pectoris, CKD, DM, HTN, prior MI and medication | High |
| Ahmed et al, 2007, USA &  Canada | RCS | 1991-1993 | 2.79 y | 2,374  HF aged 63.0 y | 69.0 | Serum | **<4.0**  **4.0-4.9** | | **NR** | Long-term mortality (820),  CVD mortality  (653) | Medical records, follow-up interviews | Age, gender, race, BMI, chest radiograph findings, creatinine, eGFR, DBP, HR, SBP, EF, NYHA class, symptoms and signs of HF, duration of HF, primary cause of HF, current angina pectoris, CKD, DM, HTN, prior MI and medication | High |

ACEI, angiotensin-converting enzyme inhibitors; AF, atrial fibrillation; AKI, acute kidney injury; ARA, angiotensin II receptor antagonists; ARB, angiotensin receptor blocker; BMI, body mass index; BP, blood pressure; CABG, coronary artery bypass grafting; CAD, coronary artery disease; CCBs, calcium channel blockers; CKD, chronic kidney disease; CK-MB, creatinine kinase-myocardial band; COPD, chronic obstructive pulmonary disease; CVD, cardiovascular disease; DBP, diastolic blood pressure; DM, diabetes mellitus; eGFR , estimated glomerular filtration rate; EF, ejection fraction; HDL, high-density lipoprotein; HF, heart failure; HR, heart rate; ICD, implantable cardioverter defibrillator; hypertension, HTN; IHD, ischemic heart disease; LDL, low-density lipoprotein; LVEF, left ventricular ejection fraction; MI, myocardial infarction; MRA, mineralocorticoid receptors antagonists; NA, not available; NIS, National Inpatient Sample; NR, not reported; NSAIDs, nonsteroidal anti-inflammatory drug; NT-ProBNP, N-terminal pro-B-type natriuretic peptide; NYHA, New York Heart Association; PAD, peripheral arterial disease; PCI, percutaneous coronary intervention; PCS, prospective cohort study; PVD, peripheral vascular disease; RAASi, renin–angiotensin–aldosterone system inhibitor; RCS, retrospective cohort study; RCT, randomized clinical trial; Ref, reference; SBP, systolic blood pressure; TIA, transient ischaemic attack; WBC, white blood cell.

* Study quality was assessed with the Newcastle-Ottawa Scale.

**^†^** The study was only included in the dose-response analysis.

^¶^ Short-term mortality:<6 months; long-term mortality:≥1 year.

Additional file 5: Table 4. Methodological quality of the included studies in the meta-analysis (n=31)

| **First author, year** | **Selection** | | | | Comparability | Assessment of exposure | | | **Total score** |
| --- | --- | --- | --- | --- | --- | --- | --- | --- | --- |
|  | Representativ  eness | Selection of the non-exposed cohort | Exposure  assessment | Demonstration of outcome not  present at start | Comparability of  cohorts on the basis of  the design or analysis^1^ | Outcome ascertainment | Adequate follow-up time^2^ | Adequacy  of follow up^3^ |  |
| Huang, 2023 | - | * | * | * | ** | * | * | * | 8 |
| Lombardi, 2022 | - | * | * | * | ** | * | * | * | 8 |
| Miura, 2022 | * | * | * | * | ** | * | * | * | 9 |
| Perez, 2022 | - | * | * | * | * | * | * | * | 7 |
| Zhang, 2022 | - | * | * | * | ** | * | - | * | 7 |
| Ferreira, 2021 | - | * | * | * | ** | * | * | * | 8 |
| Maggioni, 2021 | * | * | * | * | ** | * | * | * | 9 |
| Shiyovich, 2021 | - | * | * | * | * | * | - | * | 6 |
| Cooper, 2020 | - | * | * | * | ** | * | * | * | 8 |
| Ferreira, 2020 | - | * | * | * | ** | * | * | * | 8 |
| Valentova, 2020 | - | * | * | * | ** | * | * | * | 8 |
| Formiga,2019 | - | * | * | * | ** | * | * | * | 8 |
| Linde,2019 | * | * | * | * | ** | * | * | * | 9 |
| Legrand, 2018 | - | * | * | * | * | * | - | * | 6 |
| Núñez, 2018 | - | * | * | * | ** | * | * | * | 8 |
| Xu, 2018 | * | * | * | * | ** | * | * | * | 9 |
| Aldahl, 2017 | * | * | * | * | ** | * | - | * | 8 |
| Cheungpasitpon, 2017 | - | * | * | * | ** | * | - | * | 7 |
| Colombo, 2017 | * | * | * | * | ** | * | * | * | 9 |
| Patel, 2017 | - | * | * | * | * | * | * | * | 7 |
| Hoss, 2016 | * | * | * | * | ** | * | * | * | 9 |
| Keskin, 2016 | - | * | * | * | ** | * | * | * | 8 |
| Ma, 2016 | - | * | * | * | ** | * | - | * | 7 |
| Shu, 2016 | - | * | * | * | * | * | - | * | 7 |
| Shlomai, 2016 | - | * | * | * | * | * | * | * | 7 |
| Uluganyan, 2016 | - | * | * | * | ** | * | - | * | 7 |
| Choi, 2014 | - | * | * | * | ** | * | * | * | 8 |
| Shiyovich, 2014 | - | * | * | * | * | * | * | * | 7 |
| Goyal, 2012 | * | * | * | * | ** | * | - | * | 8 |
| Ahmed, 2010 | - | * | * | * | ** | * | * | * | 8 |
| Ahmed, 2007 | - | * | * | * | ** | * | * | * | 8 |

^1^A maximum of two stars could be awarded for this item. Studies that controlled for medical treatment received one star, whereas studies that controlled for other important confounders such as comorbidity (including hypertension or stroke) received an additional star.

^2^ A cohort study with a median follow-up time >6 months was assigned one star.

^3^ A cohort study with a follow-up rate >75% was assigned one star.

**Additional file 6: Table 5.** Subgroup analyses of the association between hypokalemia and all-cause mortality in patients with CVD

|  | In-hospital mortality | | | | | Short-term mortality | | | | | Long-term mortality | | | | |
| --- | --- | --- | --- | --- | --- | --- | --- | --- | --- | --- | --- | --- | --- | --- | --- |
| **Subgroup analysis** | **n** | **RR (95% CI)** | ***I^2^*** | ***Ph**** | ***Ph^†^*** | **n** | **RR (95% CI)** | ***I^2^*** | ***Ph**** | ***Ph^†^*** | **n** | **RR (95% CI)** | ***I^2^*** | ***Ph**** | ***Ph^†^*** |
| **Overall analysis** | **9** | **1.65 (1.22,2.25)** | **81.6** | **<0.001** | **NA** | 8 | 1.30(0.98,1.71) | 84.2 | **<0.001** | **NA** | 18 | 1.35(1.17,1.55) | 83.2 | **<0.001** | **NA** |
| **Age** |  |  |  |  | 0.50 |  |  |  |  | 0.55 |  |  |  |  | 0.94 |
| <65 | 4 | 1.85(0.85,3.99) | 75.9 | 0.006 |  | 3 | 1.03(0.65,1.66) | 0.0 | 1.00 |  | 6 | 1.36(1.06,1.73) | 76.8 | 0.001 |  |
| ≥65 | 5 | 1.50(1.12,2.01) | 79.0 | 0.006 |  | 5 | 1.36(0.99,1.87) | 90.7 | <0.001 |  | 12 | 1.35(1.12,1.62) | 86.2 | <0.001 |  |
| **Global region** |  |  |  |  | 0.52 |  |  |  |  | 0.64 |  |  |  |  | 0.83 |
| Asia | 4 | 1.29(1.00,1.66) | 10.9 | 0.34 |  | 4 | 1.00(0.79,1.26) | 0.0 | 1.00 |  | 6 | 1.47(1.13,1.93) | 81.7 | 0.001 |  |
| North America | 4 | 1.85(1.12,3.05) | 90.9 | <0.001 |  | 1 | 1.04(0.89,1.21) | NA | NA |  | 2 | 1.09(0.84,1.41) | 86.1 | 0.007 |  |
| Europe | 1 | 2.06(0.93,4.55) | NA | NA |  | 2 | 2.00(1.00,4.01) | 86.6 | 0.006 |  | 8 | 1.36(1.10,1.67) | 81.3 | <0.001 |  |
| Multi-continents | - | - | - | - |  | 1 | 1.22(1.06,1.40) | NA | NA |  | 2 | 1.40(0.70,2.79) | 93.1 | <0.001 |  |
| **Design** |  |  |  |  | 0.59 |  |  |  |  | 0.19 |  |  |  |  | 0.84 |
| Prospective | 2 | 1.34(0.77,2.36) | 52.6 | 0.15 |  | 5 | 1.53(0.88,2.65) | 75.6 | 0.003 |  | 10 | 1.34(1.10,1.62) | 84.3 | <0.001 |  |
| Retrospective | 7 | 1.74(1.20,2.53) | 83.5 | <0.001 |  | 3 | 1.10(0.97,1.25) | 36.5 | 0.21 |  | 8 | 1.38(1.12,1.70) | 80.7 | <0.001 |  |
| **Type of blood sample** |  |  |  |  | NC |  |  |  |  | 0.90 |  |  |  |  | 0.51 |
| Serum | 9 | 1.65 (1.22,2.25) | 81.6 | <0.001 |  | 7 | 1.29 (0.94,1.75) | 86.2 | <0.001 |  | 15 | 1.38 (1.16,1.65) | 85.3 | <0.001 |  |
| Plasma | - | - | - | - |  | - | - | - | - |  | 1 | 1.50(1.19,1.90) | NA | NA |  |
| Serum+ Plasma | - | - | - | - |  | 1 | 1.37(0.89,2.11) | NA | NA |  | 2 | 1.16(1.00,1.34) | 38.1 | 0.20 |  |
| **Times of potassium measurement** |  |  |  |  | 0.95 |  |  |  |  | 0.34 |  |  |  |  | 0.78 |
| One time | - | - | - | - |  | - | - | - | - |  | 2 | 1.15(0.67,1.96) | 18.9 | 0.27 |  |
| Multiple times | 4 | 1.63(1.02,2.62) | 77.7 | 0.004 |  | 2 | 1.00(0.79,1.27) | 0.0 | 0.89 |  | 7 | 1.62(1.23,2.14) | 85.4 | <0.001 |  |
| Not reported | 5 | 1.67(1.05,2.67) | 85.7 | <0.001 |  | 6 | 1.44(1.00,2.05) | 87.6 | <0.001 |  | 9 | 1.23(1.06,1.42) | 77.7 | <0.001 |  |
| **Adjustment for gender** |  |  |  |  | 0.79 |  |  |  |  | 0.49 |  |  |  |  | 0.45 |
| Yes | 7 | 1.69(1.12,2.55) | 84.0 | <0.001 |  | 7 | 1.37(0.99,1.89) | 85.3 | <0.001 |  | 15 | 1.39(1.18,1.64) | 85.9 | <0.001 |  |
| No | 2 | 1.38(0.98,1.94) | 24.1 | 0.25 |  | 1 | 0.99(0.76,1.30) | NA | NA |  | 3 | 1.17(1.02,1.35) | 0.0 | 0.75 |  |
| **Adjustment for renal function** |  |  |  |  | 0.10 |  |  |  |  | 0.87 |  |  |  |  | 0.35 |
| Yes | 3 | 2.96(2.10,4.18) | 3.5 | 0.36 |  | 4 | 1.23(1.08,1.41) | 0.0 | 0.96 |  | 12 | 1.43(1.18,1.73) | 82.2 | <0.001 |  |
| No | 6 | 1.44(1.09,1.91) | 76.7 | 0.001 |  | 4 | 1.32(0.80,2.18) | 93.1 | <0.001 |  | 6 | 1.20(1.00,1.44) | 77.3 | 0.001 |  |
| **Adjustment for HTN** |  |  |  |  | 0.65 |  |  |  |  | 0.57 |  |  |  |  | 0.69 |
| Yes | 1 | 0.87(0.07-11.1) | NA | NA |  | 4 | 1.08 (0.87-1.36) | 0.0 | 0.67 |  | 9 | 1.31(1.12,1.52) | 78.0 | <0.001 |  |
| No | 8 | 1.67(1.22-2.28) | 83.8 | <0.001 |  | 4 | 1.39(0.94-2.06) | 92.6 | <0.001 |  | 9 | 1.42(1.07,1.89) | 87.6 | <0.001 |  |
| **Adjustment for medication** |  |  |  |  | 0.70 |  |  |  |  | 0.44 |  |  |  |  | 0.64 |
| Yes | 6 | 1.72(1.13,2.62) | 86.6 | <0.001 |  | 4 | 1.07(0.93,1.23) | 0.0 | 0.71 |  | 17 | 1.37(1.17,1.59) | 84.1 | <0.001 |  |
| No | 3 | 1.31(1.10,1.56) | 0.0 | 0.49 |  | 4 | 1.46(0.88,2.43) | 91.5 | <0.001 |  | 1 | 1.18(1.00,1.40) | NA | NA |  |

CVD, cardiovascular disease; NA, not applicable; NC, not calculated; HTN, hypertension.

********Ph* values were for heterogeneity within a subgroup.

***^†^****Ph* values were for heterogeneity between subgroups by meta-regression.

**Additional file 7: Table 6.** Subgroup analyses of the association between hypokalemia and cardiovascular mortality in patients with CVD

| **Subgroup analysis** | **n** | **RR (95%CI)** | ***I^2^*** | ***Ph^*^*** | ***Ph^†^*** |
| --- | --- | --- | --- | --- | --- |
| **Overall analysis** | **6** | **1.55(1.18,2.03)** | **74.4** | **0.002** | **NA** |
| **Age** |  |  |  |  | 0.40 |
| <65 | 3 | 1.78(1.13,2.81) | 84.1 | 0.002 |  |
| ≥65 | 3 | 1.34(0.94,1.90) | 58.2 | 0.09 |  |
| **Global region** |  |  |  |  | 0.83 |
| Asia | 1 | 1.93(1.09,3.41) | NA | NA |  |
| North America | 1 | 1.27(1.06,4.51) | NA | NA |  |
| Europe | 1 | 1.45(1.01,2.09) | NA | NA |  |
| Multi-continents | 3 | 1.68(0.93,3.03) | 87.1 | <0.001 |  |
| **Design** |  |  |  |  | 0.83 |
| Prospective | 4 | 1.61(1.06,2.44) | 80.9 | 0.001 |  |
| Retrospective | 2 | 1.43(0.99,2.07) | 47.0 | 0.17 |  |
| **Type of blood sample** |  |  |  |  | 0.84 |
| Serum | 5 | 1.58(1.14,2.20) | 79.5 | 0.001 |  |
| plasma | 1 | 1.45(1.01,2.09) | NA | NA |  |
| **Times of potassium measurement** |  |  |  |  | 0.54 |
| One time | 1 | 1.93(1.09,3.41) | NA | NA |  |
| Multiple times | 3 | 1.68(0.93,3.03) | 87.1 | <0.001 |  |
| Not reported | 2 | 1.30(1.11,1.53) | 0.0 | 0.52 |  |
| **Adjustment for gender** |  |  |  |  | 0.33 |
| Yes | 3 | 1.43(1.04,1.96) | 81.6 | 0.001 |  |
| No | 2 | 2.05(1.35,3.11) | 0.0 | 0.76 |  |
| **Adjustment for renal function** |  |  |  |  | 0.71 |
| Yes | 4 | 1.50 (1.05,2.15) | 82.9 | 0.001 |  |
| No | 2 | 1.66(1.13,2.42) | 23.4 | 0.25 | 0.61 |
| **Adjustment for HTN** |  |  |  |  |  |
| Yes | 5 | 1.51(1.12,2.03) | 78.3 | 0.001 |  |
| No | 1 | 1.93(1.09,3.41) | NA | NA |  |
| **Adjustment for medication** |  |  |  |  | 0.43 |
| Yes | 5 | 1.48(1.11,1.98) | 77.2 | 0.002 |  |
| No | 1 | 2.20(1.19,4.07) | NA | NA |  |

CVD, cardiovascular disease; NA, not applicable; HTN, hypertension.

********Ph* values were for heterogeneity within a subgroup.

***^†^****Ph* values were for heterogeneity between subgroups by meta-regression.

**Additional file 8: Table 7.** Subgroup analyses of the association between hyperkalemia and all-cause mortality in patients with CVD

|  | In-hospital mortality | | | | | Short-term mortality | | | | | Long-term mortality | | | | |
| --- | --- | --- | --- | --- | --- | --- | --- | --- | --- | --- | --- | --- | --- | --- | --- |
| **Subgroup analysis** |  | **RR (95% CI)** | **I^2^(%)** | ***Ph**** | ***Ph^†^*** | **n** | **RR (95% CI)** | **I^2^(%)** | ***Ph**** | ***Ph^†^*** | **n** | **RR (95% CI)** | **I^2^(%)** | ***Ph**** | ***Ph^†^*** |
| Overall analysis | 8 | 2.78(1.92,4.03) | 87.3 | **<0.001** | NA | 7 | 1.80(1.44,2.27) | 78.9 | **<0.001** | NA | **17** | **1.33 (1.19,1.48)** | **68.8** | **<0.001** | NA |
| **Age** |  |  |  |  | 0.27 |  |  |  |  | 0.90 |  |  |  |  | 0.004 |
| <65 | 4 | 2.02(1.62,2.51) | 0.0 | 0.51 |  | 3 | 1.84(1.31,2.59) | 0.0 | 0.90 |  | 5 | 2.02(1.48,2.77) | 63.0 | 0.03 |  |
| ≥65 | 4 | 3.73(1.96,7.10) | 93.8 | <0.001 |  | 4 | 1.79(1.35,2.39) | 89.2 | <0.001 |  | 12 | 1.19(1.09,1.31) | 51.2 | 0.02 |  |
| **Global region** |  |  |  |  | 0.90 |  |  |  |  | 0.22 |  |  |  |  | 0.84 |
| Asia | 4 | 1.98(1.61,2.34) | 0.0 | 0.50 |  | 4 | 1.76(1.38,2.25) | 0.0 | 0.95 |  | 6 | 1.57(1.08,2.82) | 85.4 | <0.001 |  |
| North America | 3 | 4.32(1.87,10.0) | 94.5 | <0.001 |  | - | - | - | - |  | 1 | 1.07(0.90,1.27) | NA | NA |  |
| Europe | 1 | 2.32(1.04,5.21 | NA | NA |  | 2 | 2.20(1.94,2.51) | 0.0 | 0.64 |  | 8 | 1.30(1.18,1.44) | 31.8 | 0.17 |  |
| Multi-continents | - | - | - | - |  | 1 | 1.46(1.35,1.59) | NA | NA |  | 2 | 1.30(0.97,1.74) | 52.2 | 0.15 |  |
| **Design** |  |  |  |  | 0.81 |  |  |  |  | 0.004 |  |  |  |  | 0.39 |
| Prospective | 1 | 2.32(1.04,5.19) | NA | NA |  | 2 | 1.47(1.36,1.59) | 0.0 | 0.42 |  | 9 | 1.25(1.12,1.40) | 30.7 | 0.17 |  |
| Retrospective | 7 | 2.84(1.90,4.23) | 89.1 | <0.001 |  | 5 | 2.15(1.91,2.43) | 0.0 | 0.85 |  | 8 | 1.47(1.19,1.82) | 82.1 | <0.001 |  |
| **Times of potassium measurement** |  |  |  |  | 0.38 |  |  |  |  | 0.84 |  |  |  |  | 0.56 |
| One time | - | - | - | - |  | - | - | - | - |  | 2 | 1.31(0.68,2.53) | 68.7 | 0.08 |  |
| Multiple times | 4 | 3.52(1.77,7.02) | 92.3 | <0.001 |  | 2 | 1.74(1.34,2.25) | 0.0 | 0.81 |  | 7 | 1.44(1.21,1.72) | 69.4 | 0.003 |  |
| Not reported | 4 | 1.97(1.69,2.31) | 0.0 | 0.87 |  | 5 | 1.84(1.35,2.51) | 85.8 | <0.001 |  | 8 | 1.26(1.07,1.48) | 69.0 | 0.002 |  |
| **Type of blood sample** |  |  |  |  | NC |  |  |  |  | 0.80 |  |  |  |  | 1.00 |
| Serum | 8 | 2.78(1.92,4.03) | 89.6 | <0.001 |  | 6 | 1.79(1.39,2.29) | 82.1 | <0.001 |  | 14 | 1.30(1.15,1.45) | 64.5 | <0.001 |  |
| Plasma | - | - | - | - |  | - | - | - | - |  | 1 | 1.48(0.99,2.22) | NA | NA |  |
| Serum+plasma | - | - | - | - |  | 1 | 1.95(1.15,3.30) | NA | NA |  | 2 | 1.54(0.73,3.28) | 92.8 | <0.001 |  |
| **Adjustment for gender** |  |  |  |  | 0.54 |  |  |  |  | 0.80 |  |  |  |  | 0.39 |
| Yes | 6 | 3.09(1.87,5.12) | 88.9 | <0.001 |  | 7 | 1.83(1.40,2.39) | 82.4 | <0.001 |  | 14 | 1.38(1.21,1.57) | 72.6 | <0.001 |  |
| No | 2 | 1.96(1.63,2.35) | 0.0 | 0.67 |  | 1 | 1.69(1.20,2.38) | NA | NA |  | 3 | 1.16(1.01,1.34) | 0.0 | 0.38 |  |
| **Adjustment for renal function** |  |  |  |  | 0.62 |  |  |  |  | 0.12 |  |  |  |  | 0.96 |
| Yes | 3 | 2.06(1.54,2.77) | 0.0 | 0.74 |  | 4 | 1.48(1.36,1.60) | 0.0 | 0.58 |  | 11 | 1.33(1.16,1.53) | 71.3 | <0.001 |  |
| No | 5 | 3.07(1.85,5.07) | 92.5 | <0.001 |  | 3 | 2.03(1.70,2.42) | 29.2 | 0.24 |  | 3 | 1.33(1.08,1.64) | 65.5 | 0.02 |  |
| **Adjustment for HTN** |  |  |  |  | 0.94 |  |  |  |  | 0.95 |  |  |  |  | 0.28 |
| Yes | 1 | 3.02(0.96-9.55) | NA | NA |  | 4 | 1.79(1.37-2.34) | 0.0 | 0.93 |  | 9 | 1.24(1.07-1.44) | 67.2 | 0.002 |  |
| No | 7 | 2.77(1.87-4.08) | 89.1 | <0.001 |  | 3 | 1.80(1.29-2.50) | 92.7 | <0.001 |  | 8 | 1.46(1.22-1.74) | 65.5 | 0.005 |  |
| **Adjustment for medication** |  |  |  |  | 0.60 |  |  |  |  | 0.97 |  |  |  |  | 0.58 |
| Yes | 5 | 3.10(1.80,5.35) | 91.1 | <0.001 |  | 3 | 1.84(1.35,2.49) | 0.0 | 0.94 |  | 16 | 1.35(1.19,1.52) | 70.0 | <0.001 |  |
| No | 3 | 1.98(1.65,2.37) | 0.0 | 0.70 |  | 4 | 1.80(1.44,2.27) | 89.2 | <0.001 |  | 1 | 1.16(0.98,1.37) | NA | NA |  |

CVD, cardiovascular disease; NA, not applicable; NC, not calculated; HTN, hypertension.

********Ph* values were for heterogeneity within a subgroup.

***^†^****Ph* values were for heterogeneity between subgroups by meta-regression

**Additional file 9: Table 8.** Subgroup analyses of the association between hyperkalemia and cardiovascular mortality in patients with CVD

| **Subgroup analysis** | **n** | **RR (95%CI)** | ***I^2^*** | ***Ph^*^*** | ***Ph^†^*** |
| --- | --- | --- | --- | --- | --- |
| **Overall analysis** | 6 | 1.19(1.04,1.36) | 0.0 | 0.47 | NA |
| **Age** |  |  |  |  | 0.11 |
| <65 | 2 | 1.52(1.16,1.99) | 0.0 | 0.79 |  |
| ≥65 | 4 | 1.10(0.94,1.28) | 0.0 | 0.97 |  |
| **Global region** |  |  |  |  | 0.44 |
| Asia | 1 | 1.06(0.66,1.70) | NA | NA |  |
| North America | 1 | 1.08(0.89,1.30) | NA | NA |  |
| Europe | 1 | 1.26(0.63,2.52) | NA | NA |  |
| Multi-continents | 3 | 1.37(1.10,1.69) | 0.0 | 0.42 |  |
| **Design** |  |  |  |  | 0.17 |
| Prospective | 4 | 1.36(1.10,1.67) | 0.0 | 0.62 |  |
| Retrospective | 2 | 1.08(0.90,1.28) | 0.0 | 0.94 |  |
| **Type of blood sample** |  |  |  |  | 0.91 |
| Serum | 5 | 1.20(1.03,1.39) | 11.8 | 0.34 |  |
| plasma | 1 | 1.26(0.63,2.52) | NA | NA |  |
| **Times of potassium measurement** |  |  |  |  | 0.92 |
| One time | 1 | 1.06(0.66,1.70) | NA | NA |  |
| Multiple times | 3 | 1.37(1.10,1.69) | 0.0 | **0**.42 |  |
| Not reported | 2 | 1.09(0.91,1.31) | 0.0 | **0**.67 |  |
| **Adjustment for gender** |  |  |  |  | **0.**64 |
| Yes | 4 | 1.17(1.01,1.35) | 0.0 | 0.44 |  |
| No | 2 | 1.31(0.87,1.96) | **32.7** | **0.22** |  |
| **Adjustment for renal function** |  |  |  |  | 0.29 |
| Yes | 4 | 1.15(1.00,1.33) | 0.0 | 0.43 |  |
| No | 2 | 1.49(1.01,2.18) | 0.0 | 0.57 |  |
| **Adjustment for HTN** |  |  |  |  | 0.61 |
| Yes | 5 | 1.21(104,1.41) | 7.6 | 0.36 |  |
| No | 1 | 1.06(0.66,1.70) | NA | NA |  |
| **Adjustment for medication** |  |  |  |  | 0.26 |
| Yes | 5 | 1.16(1.00,1.33) | 0.0 | 0.44 |  |
| No | 1 | 1.60(1.01,2.54) | NA | NA |  |

CVD, cardiovascular disease; NA, not applicable; HTN, hypertension.

********Ph* values were for heterogeneity within a subgroup.

***^†^****Ph* values were for heterogeneity between subgroups by meta-regression.

Supplementary **Figures**

**Additional file 10: Figure 1.** Forest plot for association between blood potassium levels and risk of in-hospital mortality in patients with total cardiovascular diseases, expressed as comparison between hypokalemia and normokalemia.

**
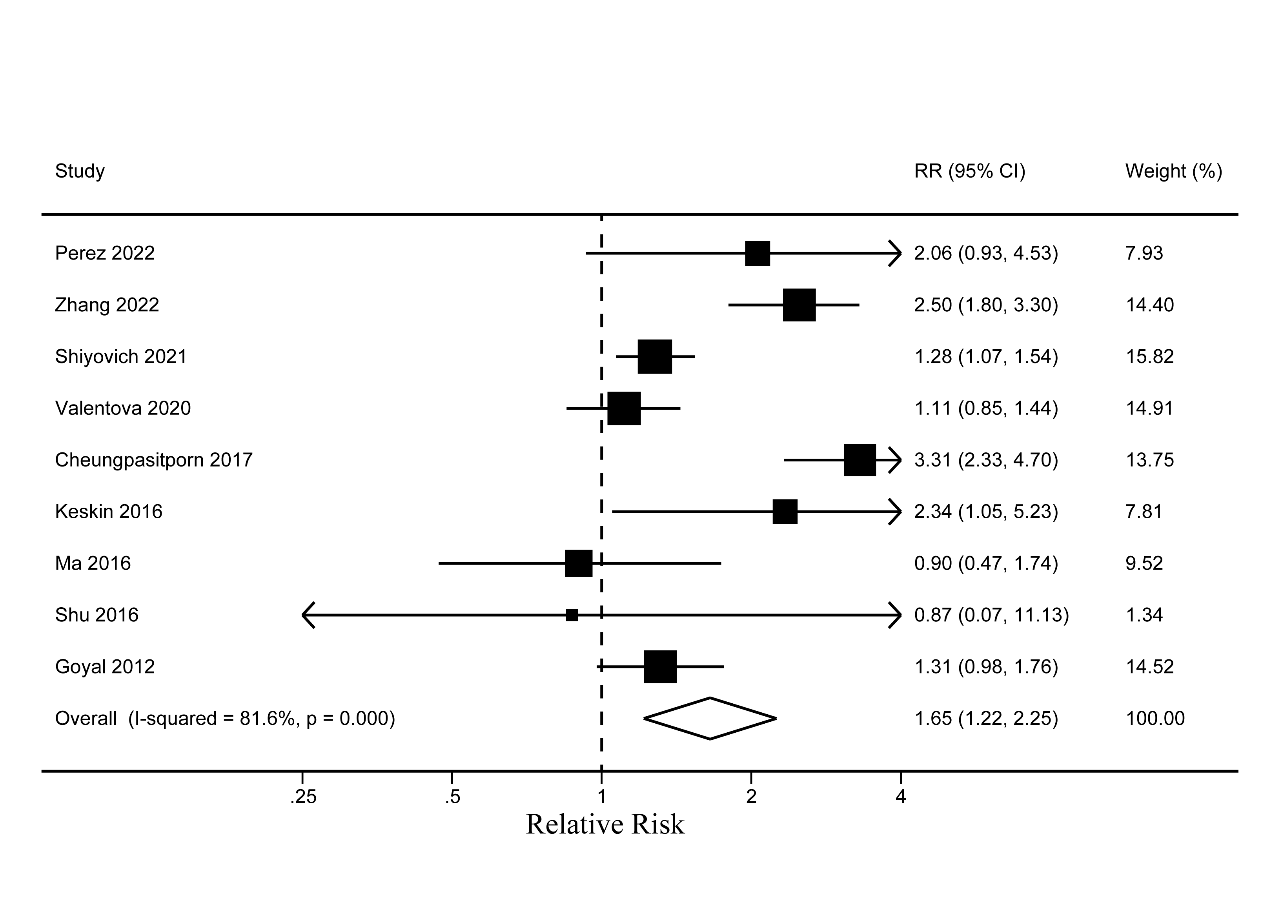
**

The size of the black squares reflects the relative statistical weight of study-specific estimate, horizontal lines indicate 95% CIs. The diamond indicates the pooled RR estimates with 95% CI. CI, confidence interval; RR, relative risk.

**Additional file 11: Figure 2.** Forest plot for association between blood potassium levels and risk of in-hospital mortality in patients with myocardial infarction, expressed as comparison between hypokalemia and normokalemia.


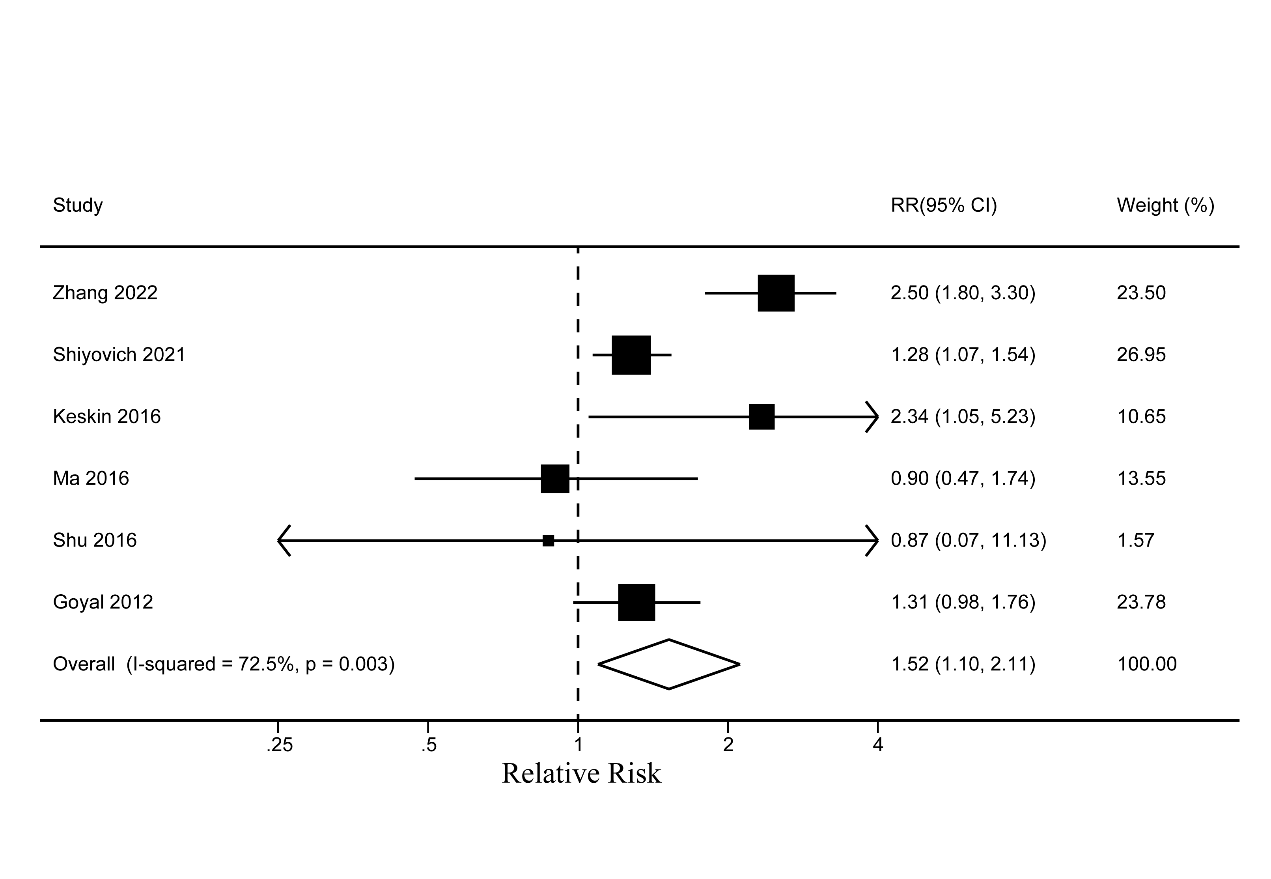
The size of the black squares reflects the relative statistical weight of study-specific estimate, horizontal lines indicate 95% CIs. The diamond indicates the pooled RR estimates with 95% CI. CI, confidence interval; RR, relative risk.

**Additional file 12: Figure 3.** Forest plot for association between blood potassium levels and risk of in-hospital mortality in patients with heart failure, expressed as comparison between hypokalemia and normokalemia.


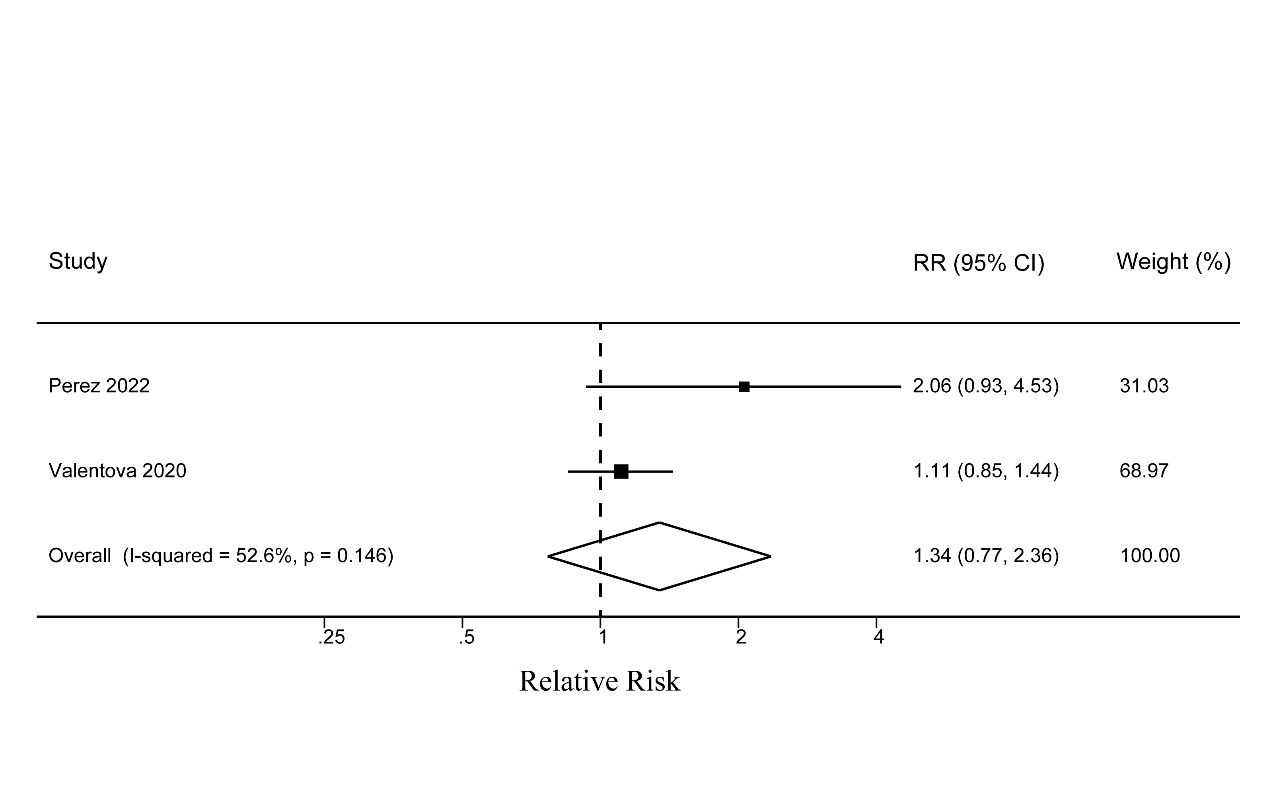


The size of the black squares reflects the relative statistical weight of study-specific estimate, horizontal lines indicate 95% CIs. The diamond indicates the pooled RR estimates with 95% CI. CI, confidence interval; RR, relative risk.

**Additional file 13: Figure 4.** Forest plot for association between blood potassium levels and risk of short-term mortality in patients with total cardiovascular diseases, expressed as comparison between hypokalemia and normokalemia.

**
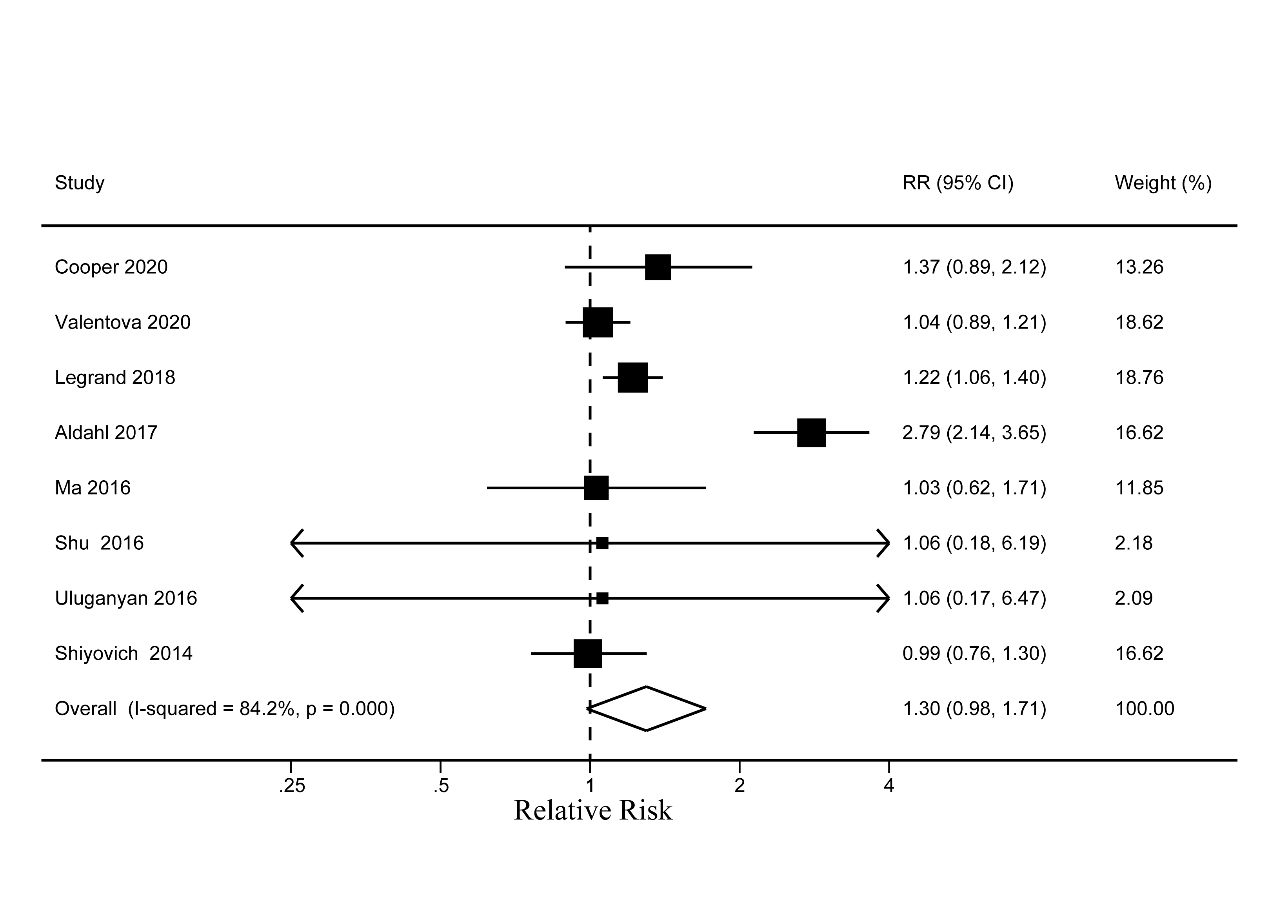
**

The size of the black squares reflects the relative statistical weight of study-specific estimate, horizontal lines indicate 95% CIs. The diamond indicates the pooled RR estimates with 95% CI. CI, confidence interval; RR, relative risk.

**Additional file 14: Figure 5.** Forest plot for association between blood potassium levels and risk of short-term mortality in patients with myocardial infarction, expressed as comparison between hypokalemia and normokalemia.


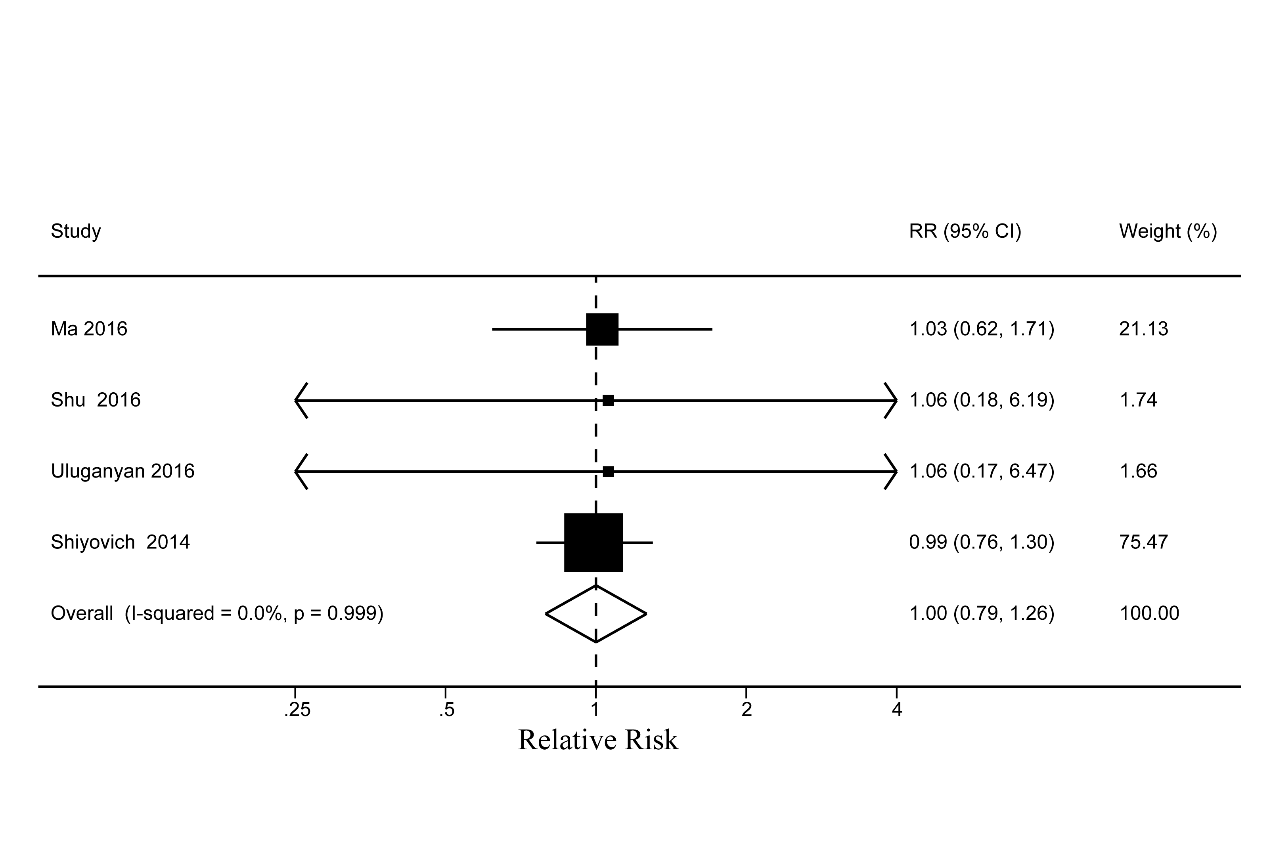


The size of the black squares reflects the relative statistical weight of study-specific estimate, horizontal lines indicate 95% CIs. The diamond indicates the pooled RR estimates with 95% CI. CI, confidence interval; RR, relative risk.

**Additional file 15: Figure 6.** Forest plot for association between blood potassium levels and risk of short-term mortality in patients with heart failure, expressed as comparison between hypokalemia and normokalemia.

**
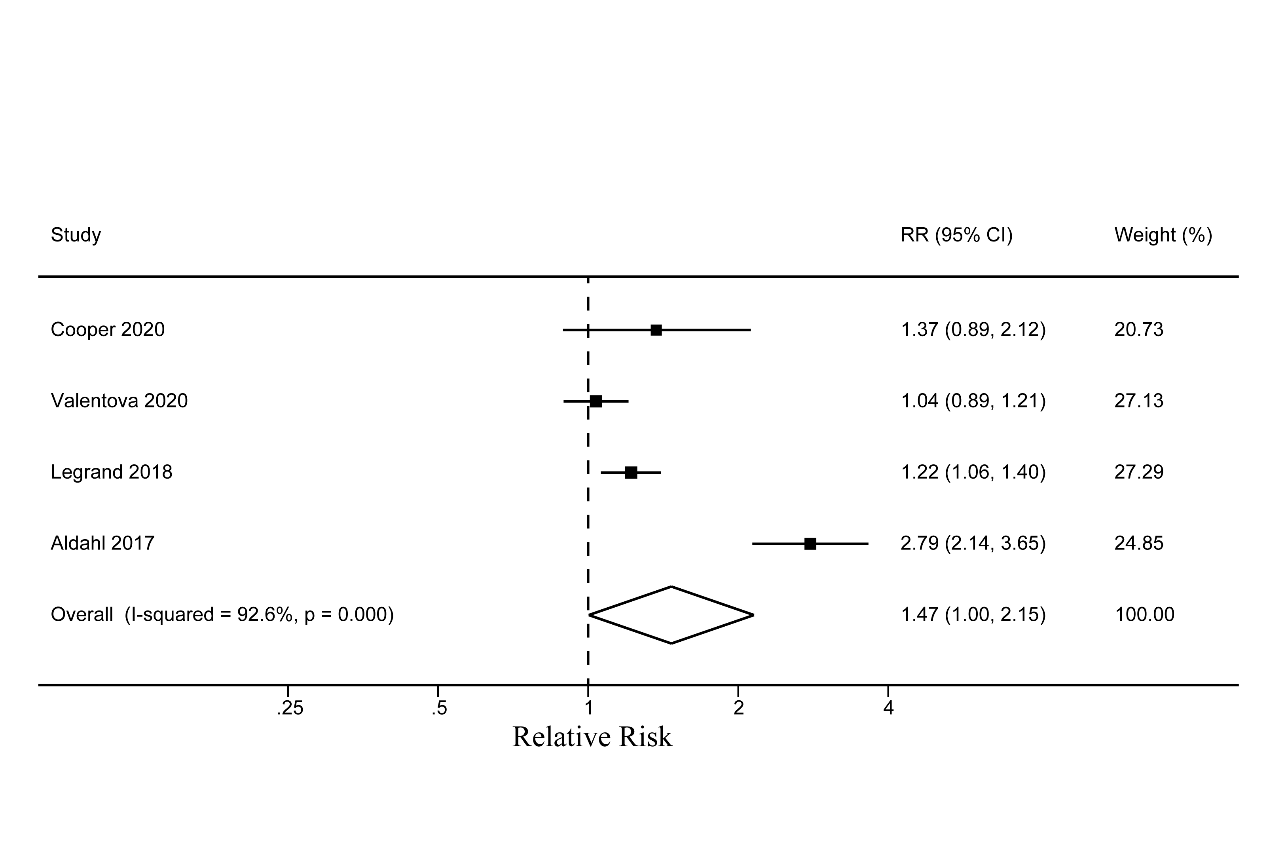
**

The size of the black squares reflects the relative statistical weight of study-specific estimate, horizontal lines indicate 95% CIs. The diamond indicates the pooled RR estimates with 95% CI. CI, confidence interval; RR, relative risk.

**Additional file 16: Figure 7.** Forest plot for association between blood potassium levels and risk of long-term mortality in patients with total cardiovascular diseases, expressed as comparison between hypokalemia and normokalemia.


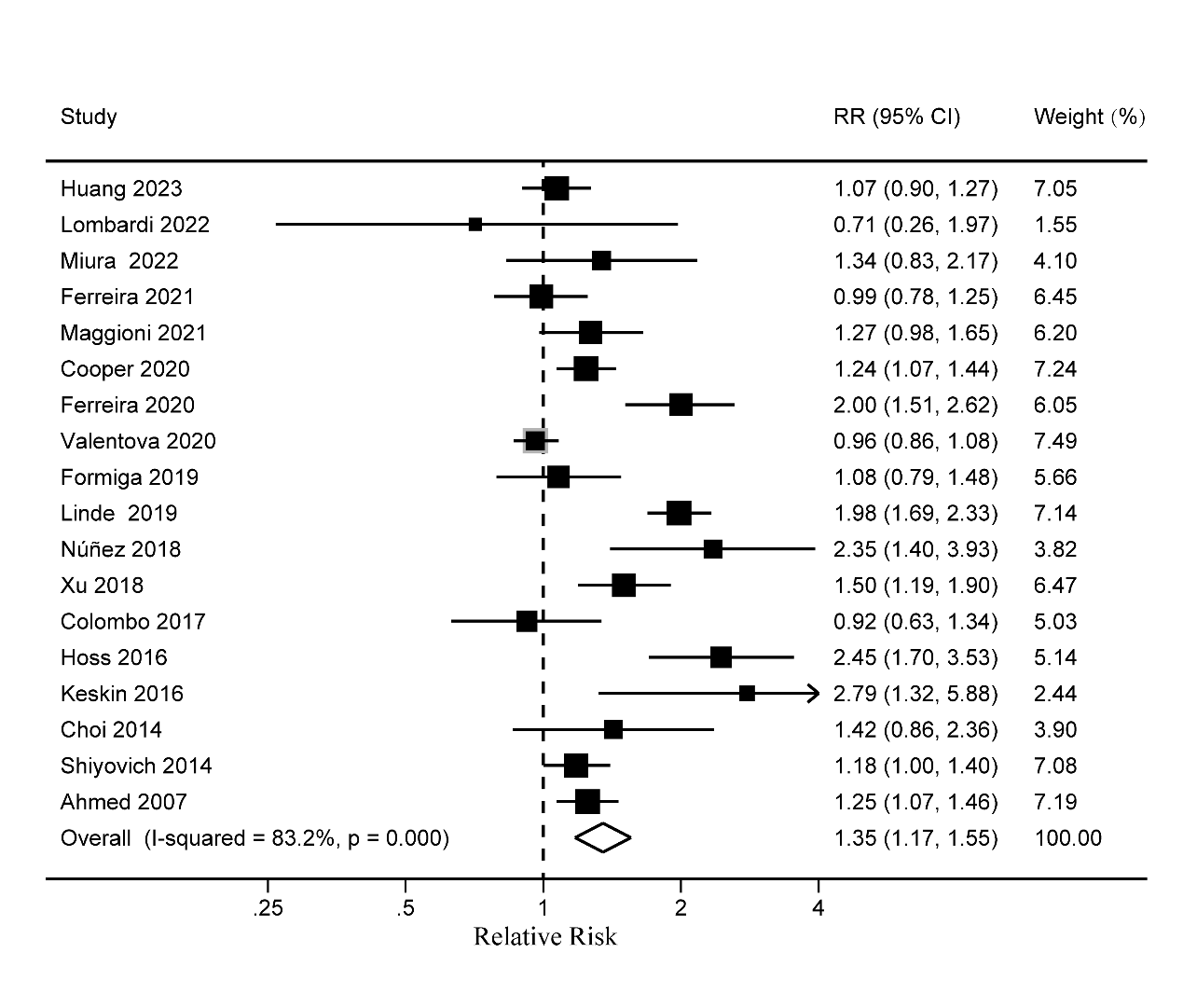
The size of the black squares reflects the relative statistical weight of study-specific estimate, horizontal lines indicate 95% CIs. The diamond indicates the pooled RR estimates with 95% CI. CI, confidence interval; RR, relative risk.

**Additional file 17: Figure 8.** Forest plot for association between blood potassium levels and risk of long-term mortality in patients with myocardial infarction, expressed as comparison between hypokalemia and normokalemia.


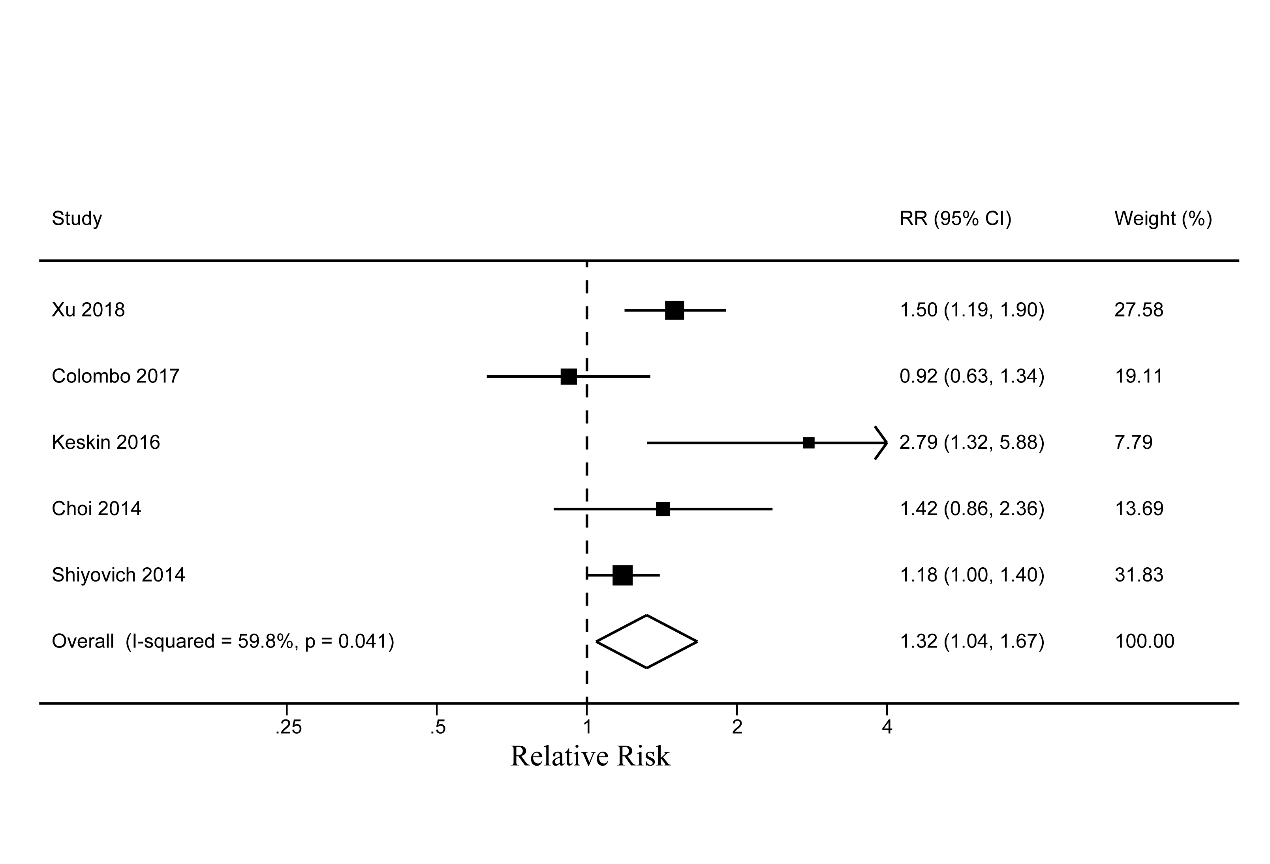


The size of the black squares reflects the relative statistical weight of study-specific estimate, horizontal lines indicate 95% CIs. The diamond indicates the pooled RR estimates with 95% CI. CI, confidence interval; RR, relative risk.

**Additional file 18: Figure 9.** Forest plot for association between blood potassium levels and risk of long-term mortality in patients with heart failure, expressed as comparison between hypokalemia and normokalemia.

**
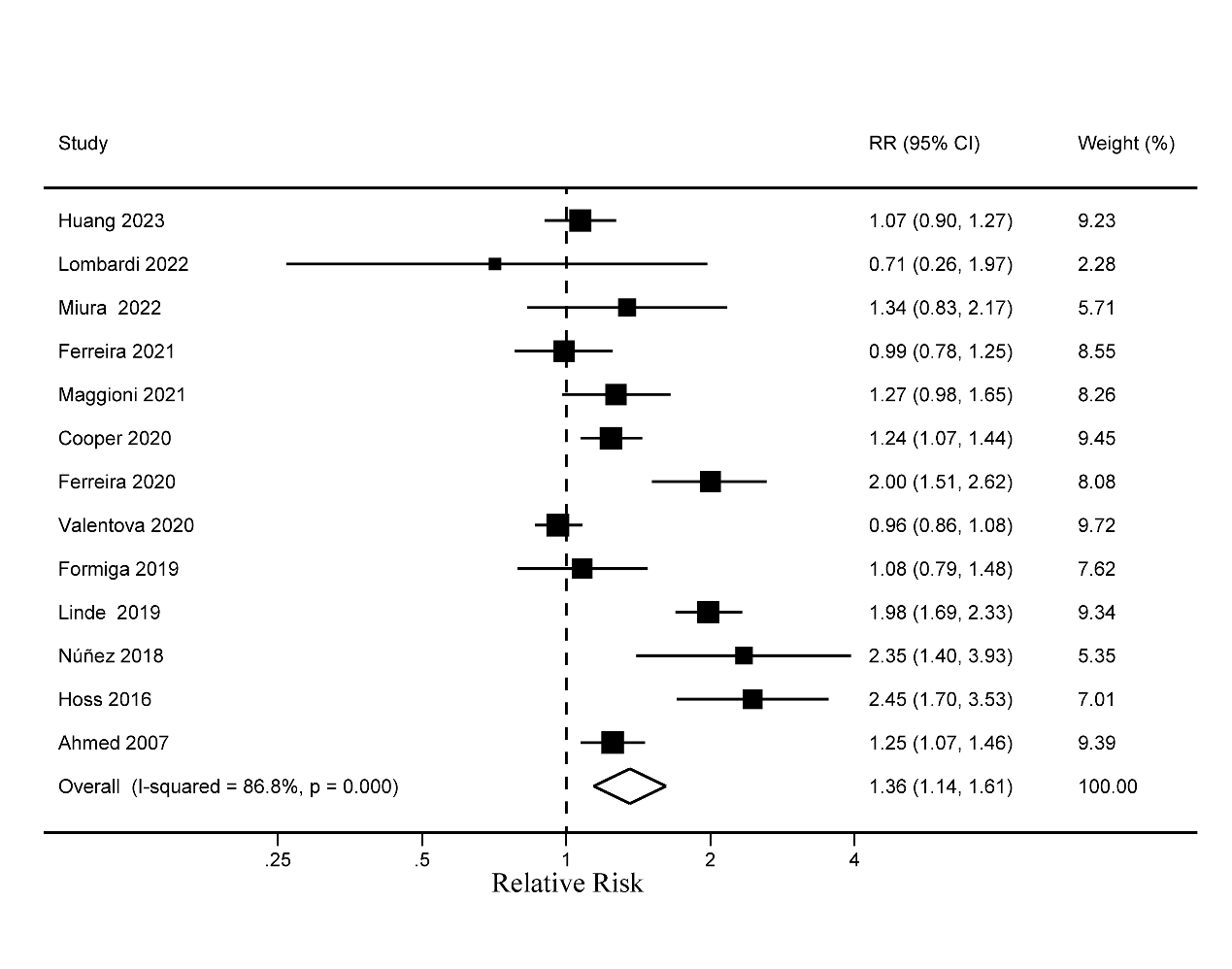
**

The size of the black squares reflects the relative statistical weight of study-specific estimate, horizontal lines indicate 95% CIs. The diamond indicates the pooled RR estimates with 95% CI. CI, confidence interval; RR, relative risk.

**Additional file 19: Figure 10.** Forest plot for association between blood potassium levels and risk of cardiovascular mortality in patients with total cardiovascular diseases, expressed as comparison between hypokalemia and normokalemia.


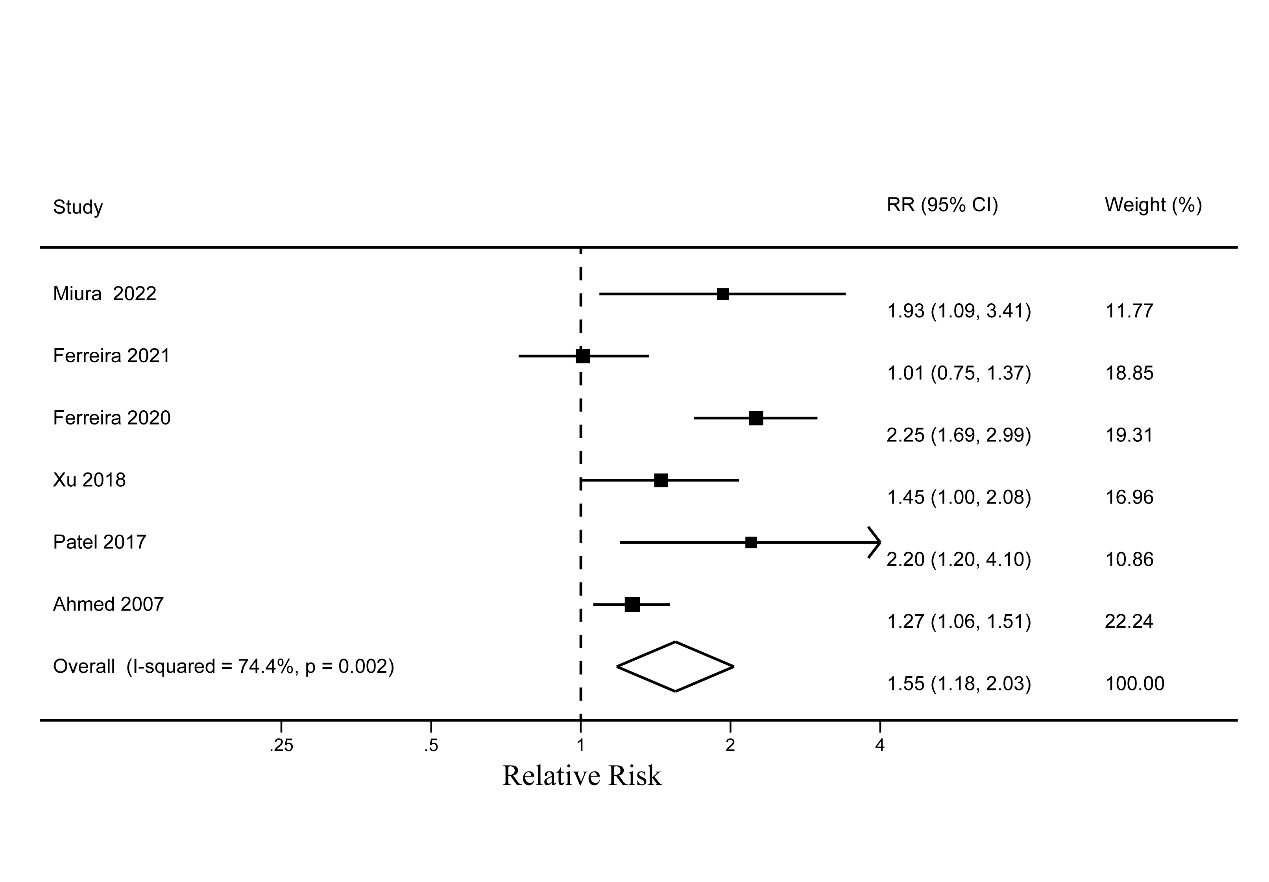


The size of the black squares reflects the relative statistical weight of study-specific estimate, horizontal lines indicate 95% CIs. The diamond indicates the pooled RR estimates with 95% CI. CI, confidence interval; RR, relative risk.

**Additional file 20: Figure 11.** Forest plot for association between blood potassium levels and risk of in-hospital mortality in patients with total cardiovascular diseases, expressed as comparison between hyperkalemia and normokalemia.

**
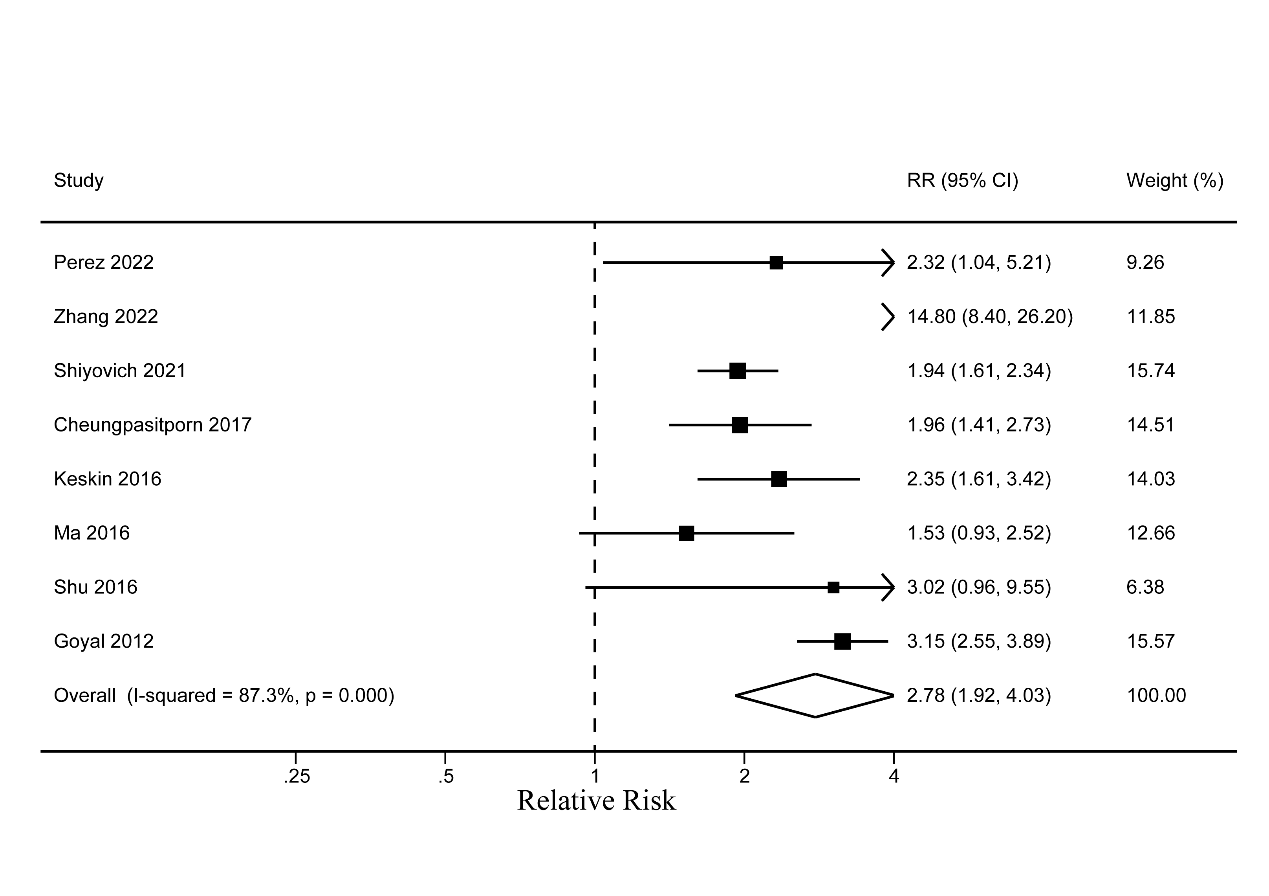
**

The size of the black squares reflects the relative statistical weight of study-specific estimate, horizontal lines indicate 95% CIs. The diamond indicates the pooled RR estimates with 95% CI. CI, confidence interval; RR, relative risk.

**Additional file 21: Figure 12.** Forest plot for association between blood potassium levels and risk of in-hospital mortality in patients with myocardial infarction, expressed as comparison between hyperkalemia and normokalemia.

**
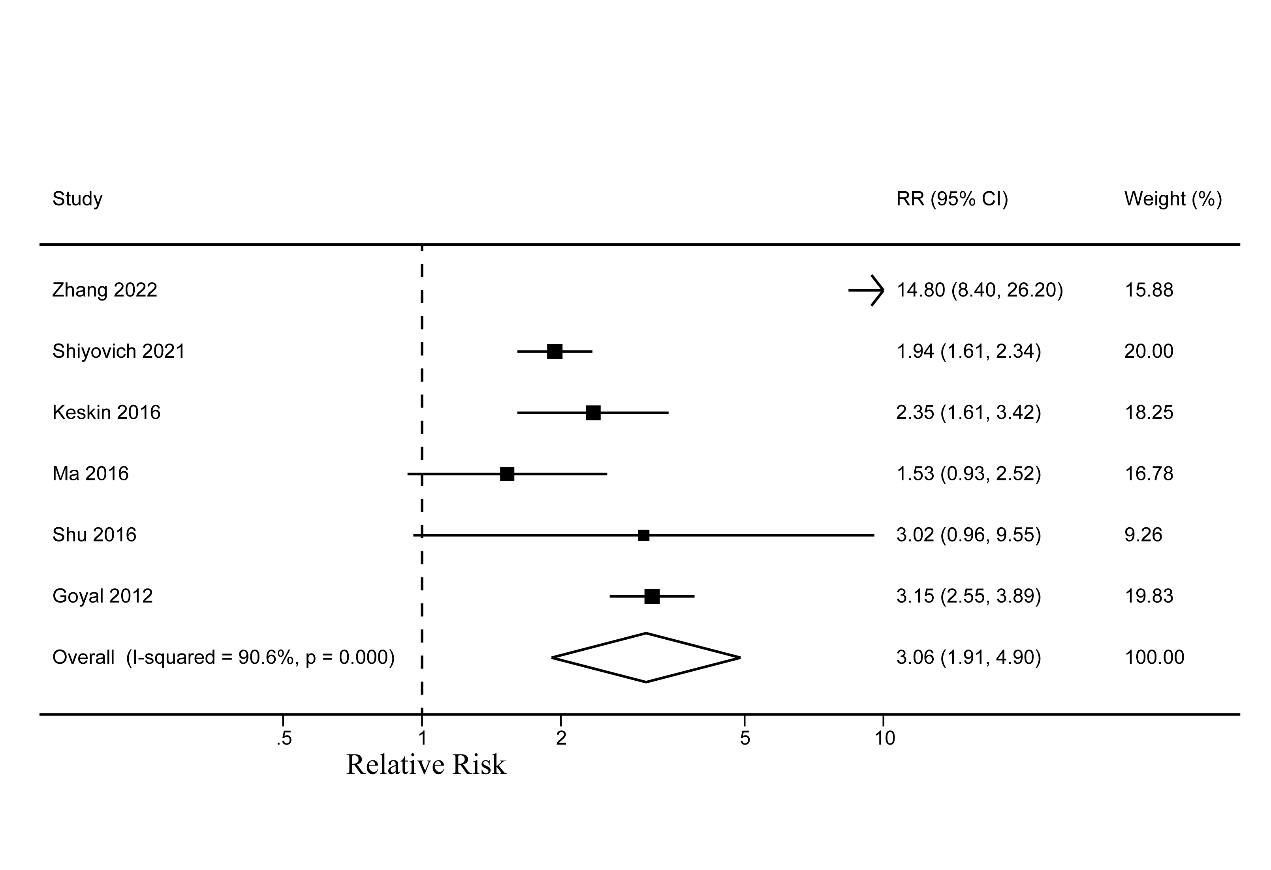
**

The size of the black squares reflects the relative statistical weight of study-specific estimate, horizontal lines indicate 95% CIs. The diamond indicates the pooled RR estimates with 95% CI. CI, confidence interval; RR, relative risk.

**Additional file 22: Figure 13.** Forest plot for association between blood potassium levels and risk of short-term mortality in patients with total cardiovascular diseases, expressed as comparison between hyperkalemia and normokalemia.

**
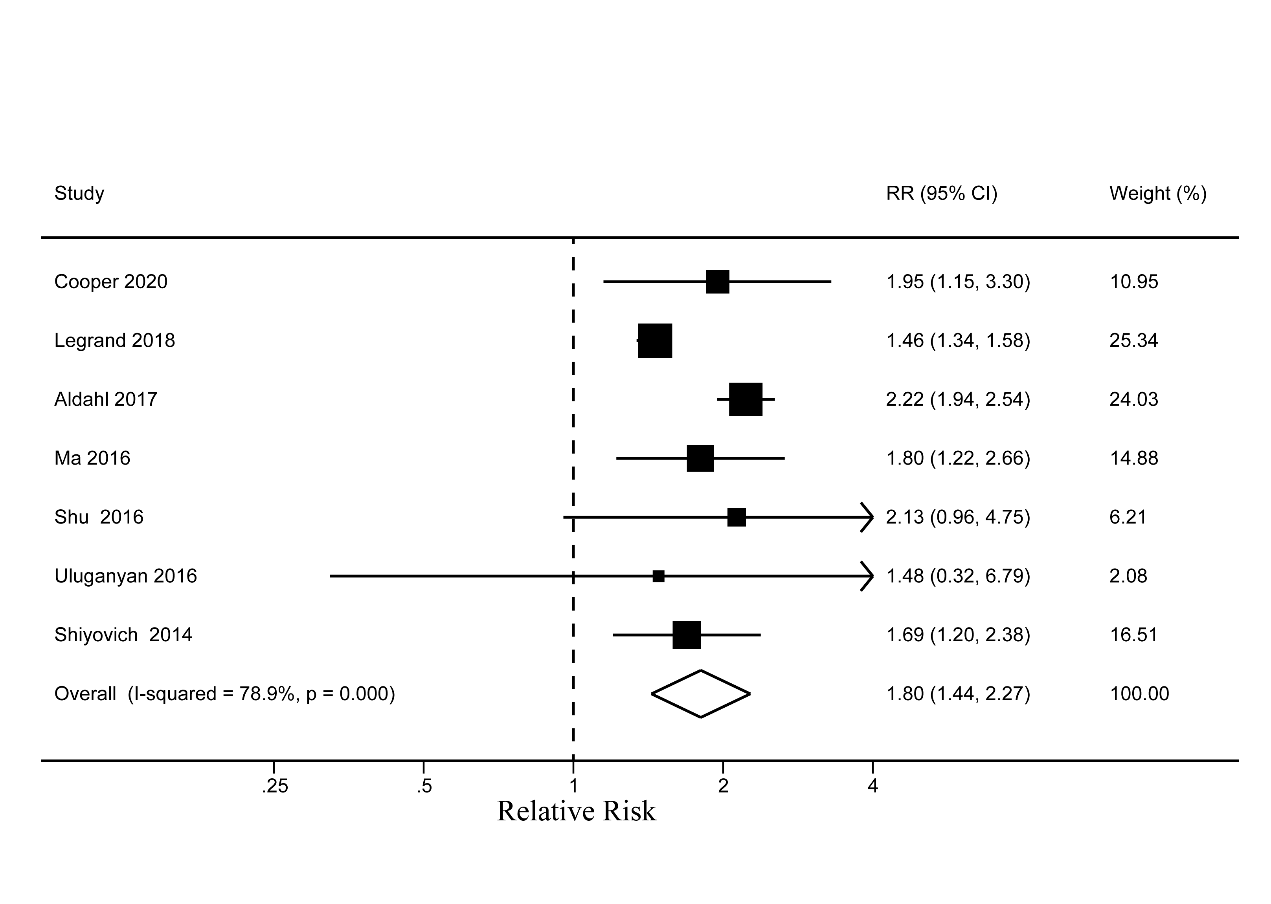
**

The size of the black squares reflects the relative statistical weight of study-specific estimate, horizontal lines indicate 95% CIs. The diamond indicates the pooled RR estimates with 95% CI. CI, confidence interval; RR, relative risk.

**Additional file 23: Figure 14.** Forest plot for association between blood potassium levels and risk of short-term mortality in patients with myocardial infarction, expressed as comparison between hyperkalemia and normokalemia.

**
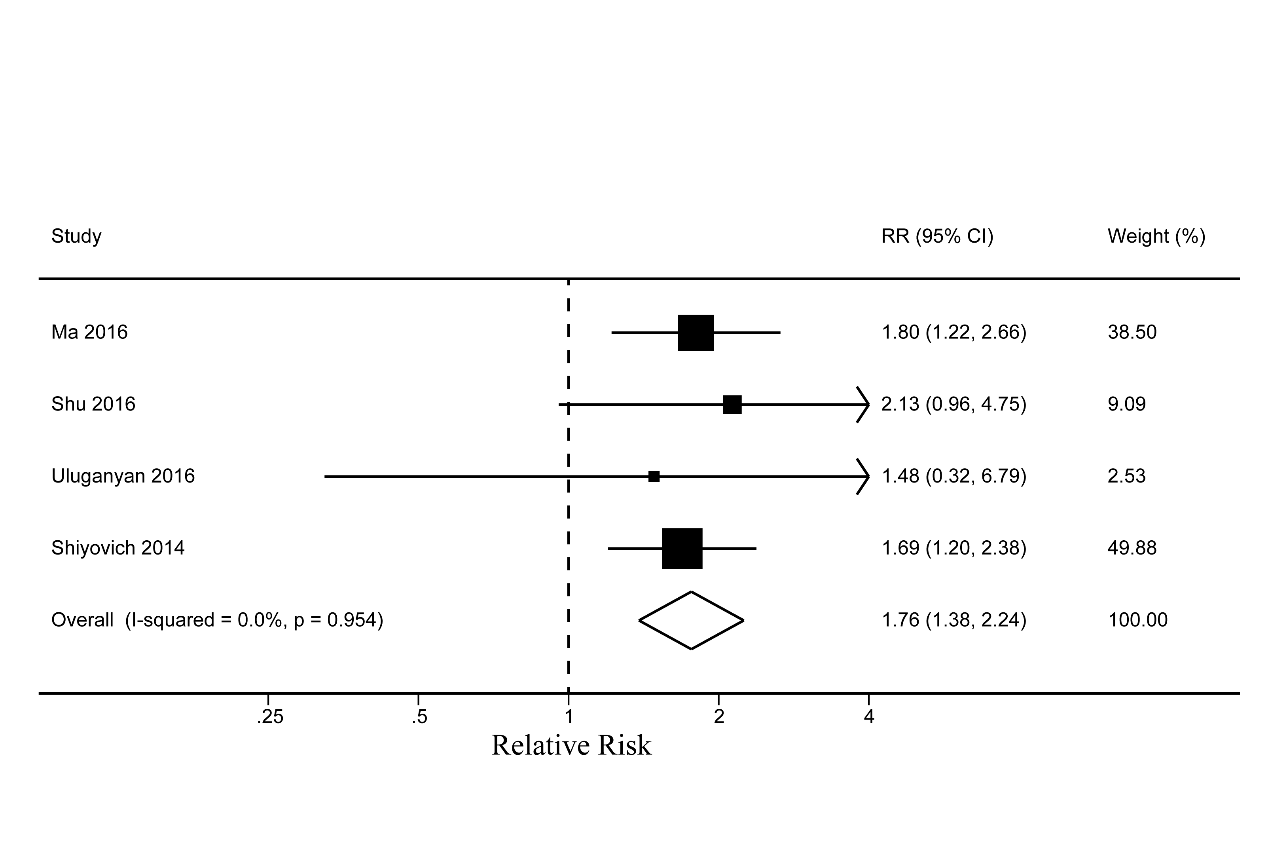
**

The size of the black squares reflects the relative statistical weight of study-specific estimate, horizontal lines indicate 95% CIs. The diamond indicates the pooled RR estimates with 95% CI. CI, confidence interval; RR, relative risk.

**Additional file 24: Figure 15.** Forest plot for association between blood potassium levels and risk of short-term mortality in patients with heart failure, expressed as comparison between hyperkalemia and normokalemia.


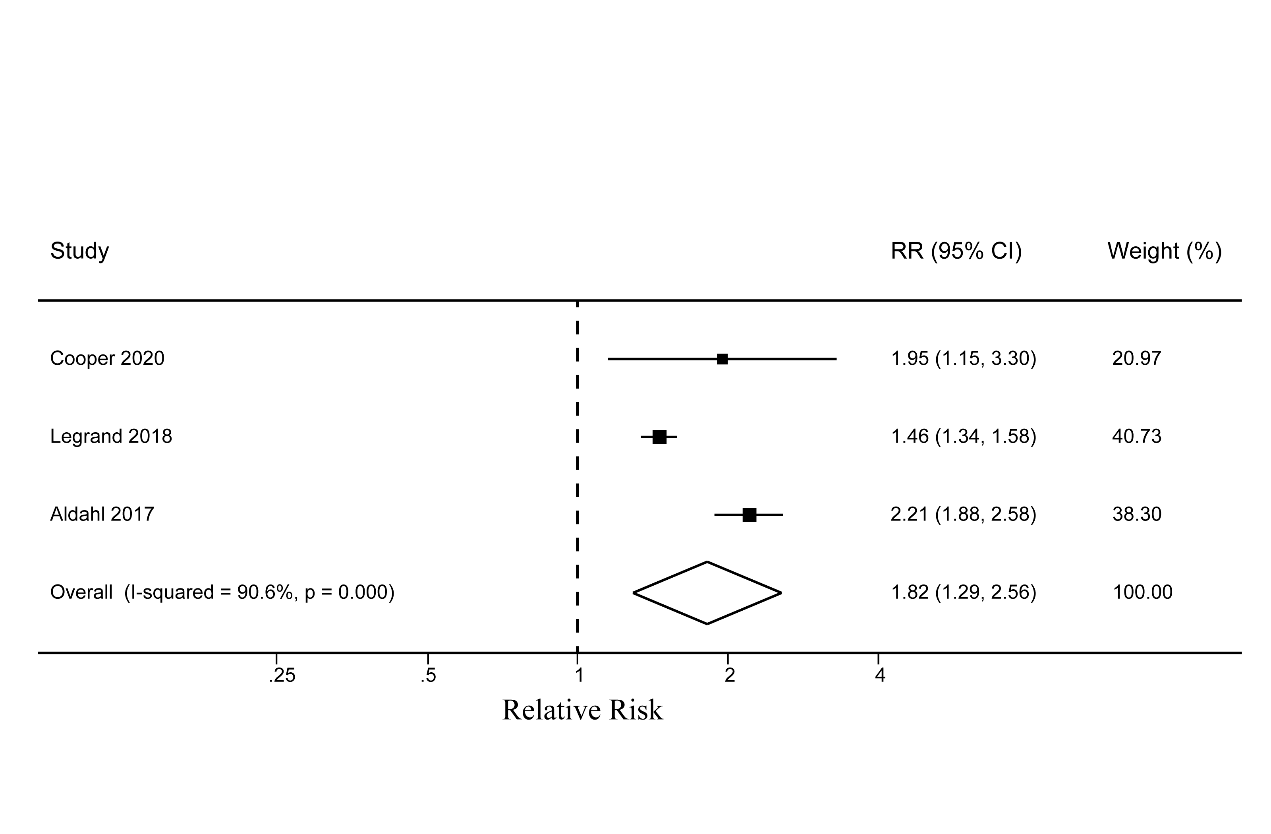


The size of the black squares reflects the relative statistical weight of study-specific estimate, horizontal lines indicate 95% CIs. The diamond indicates the pooled RR estimates with 95% CI. CI, confidence interval; RR, relative risk.

**Additional file 25: Figure 16.** Forest plot for association between blood potassium levels and risk of long-term mortality in patients with total cardiovascular diseases, expressed as comparison between hyperkalemia and normokalemia.

**
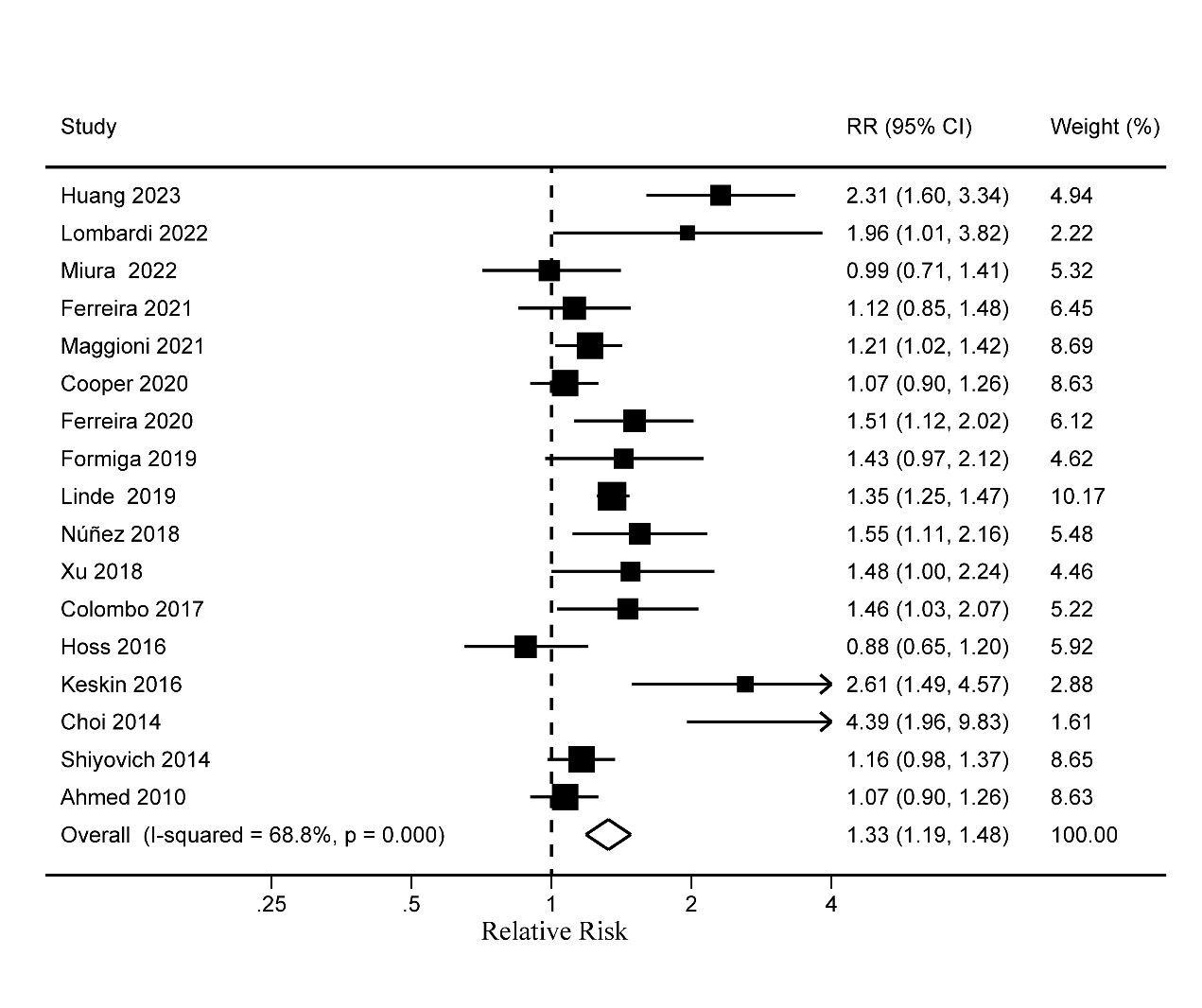
**The size of the black squares reflects the relative statistical weight of study-specific estimate, horizontal lines indicate 95% CIs. The diamond indicates the pooled RR estimates with 95% CI. CI, confidence interval; RR, relative risk.

**Additional file 26: Figure 17.** Forest plot for association between blood potassium levels and risk of long-term mortality in patients with myocardial infarction, expressed as comparison between hyperkalemia and normokalemia.

**
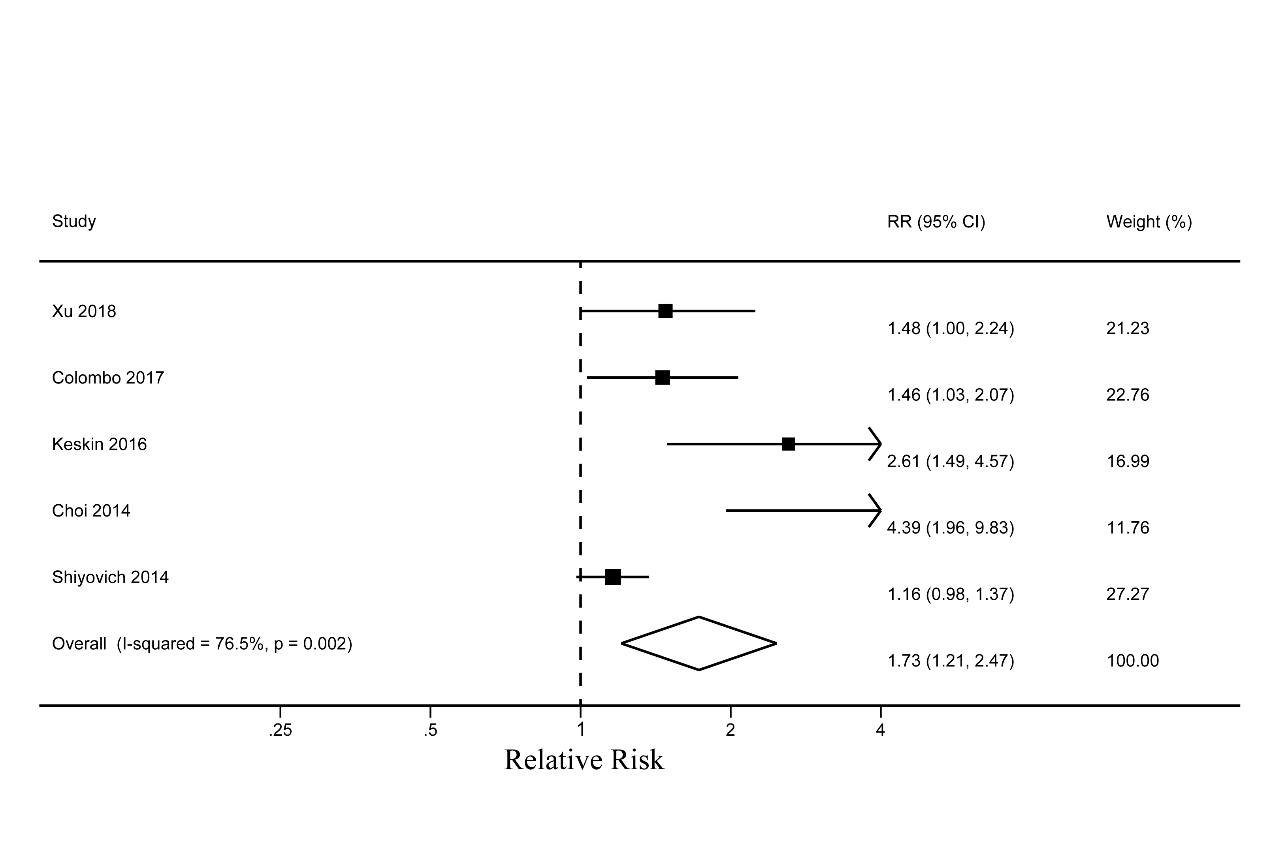
**

The size of the black squares reflects the relative statistical weight of study-specific estimate, horizontal lines indicate 95% CIs. The diamond indicates the pooled RR estimates with 95% CI. CI, confidence interval; RR, relative risk.

**Additional file 27: Figure 18.** Forest plot for association between blood potassium levels and risk of long-term mortality in patients with heart failure, expressed as comparison between hyperkalemia and normokalemia.

**
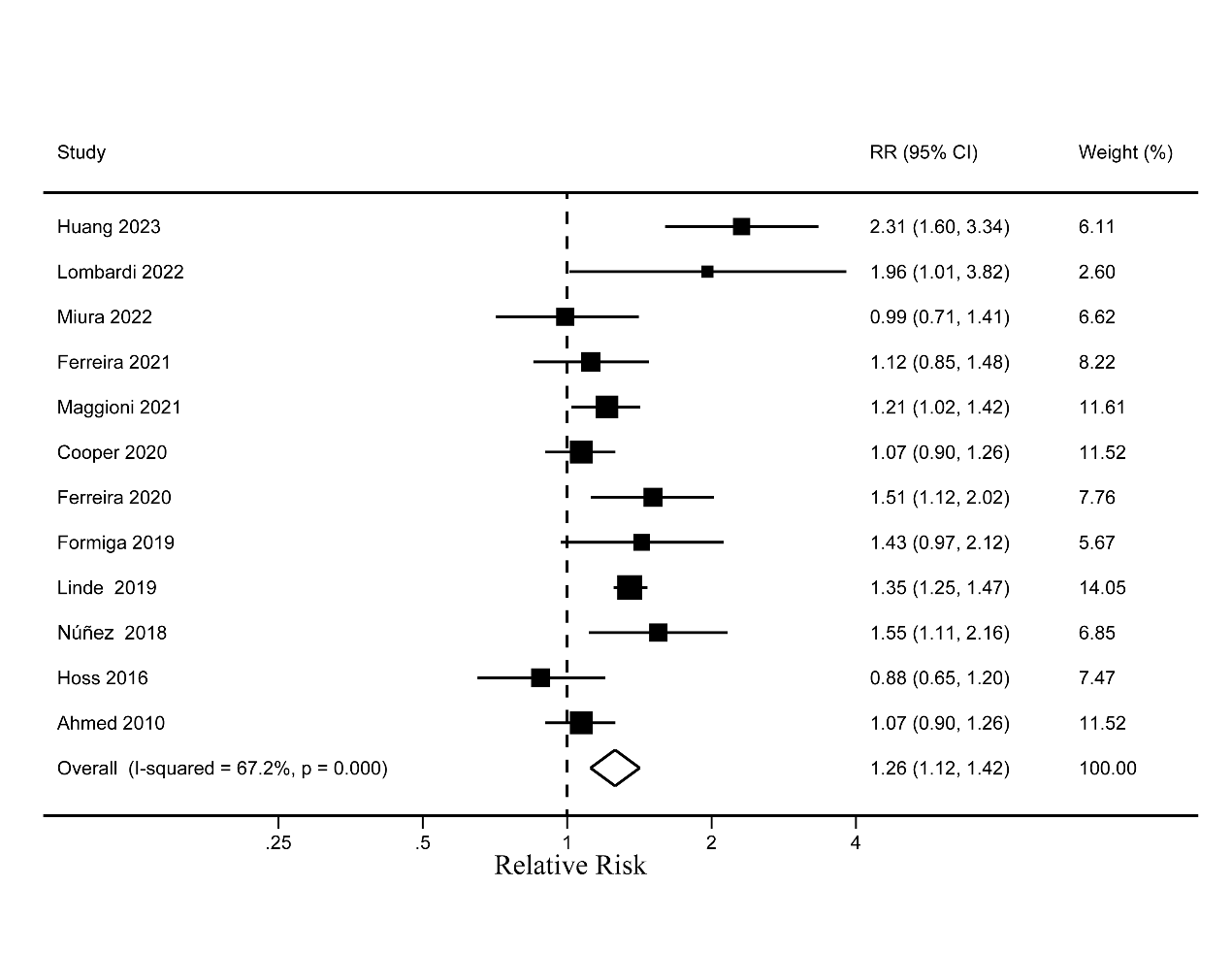
**

The size of the black squares reflects the relative statistical weight of study-specific estimate, horizontal lines indicate 95% CIs. The diamond indicates the pooled RR estimates with 95% CI. CI, confidence interval; RR, relative risk.

**Additional file 28: Figure 19.** Forest plot for association between blood potassium levels and risk of cardiovascular mortality in patients with total cardiovascular diseases, expressed as comparison between hyperkalemia and normokalemia.


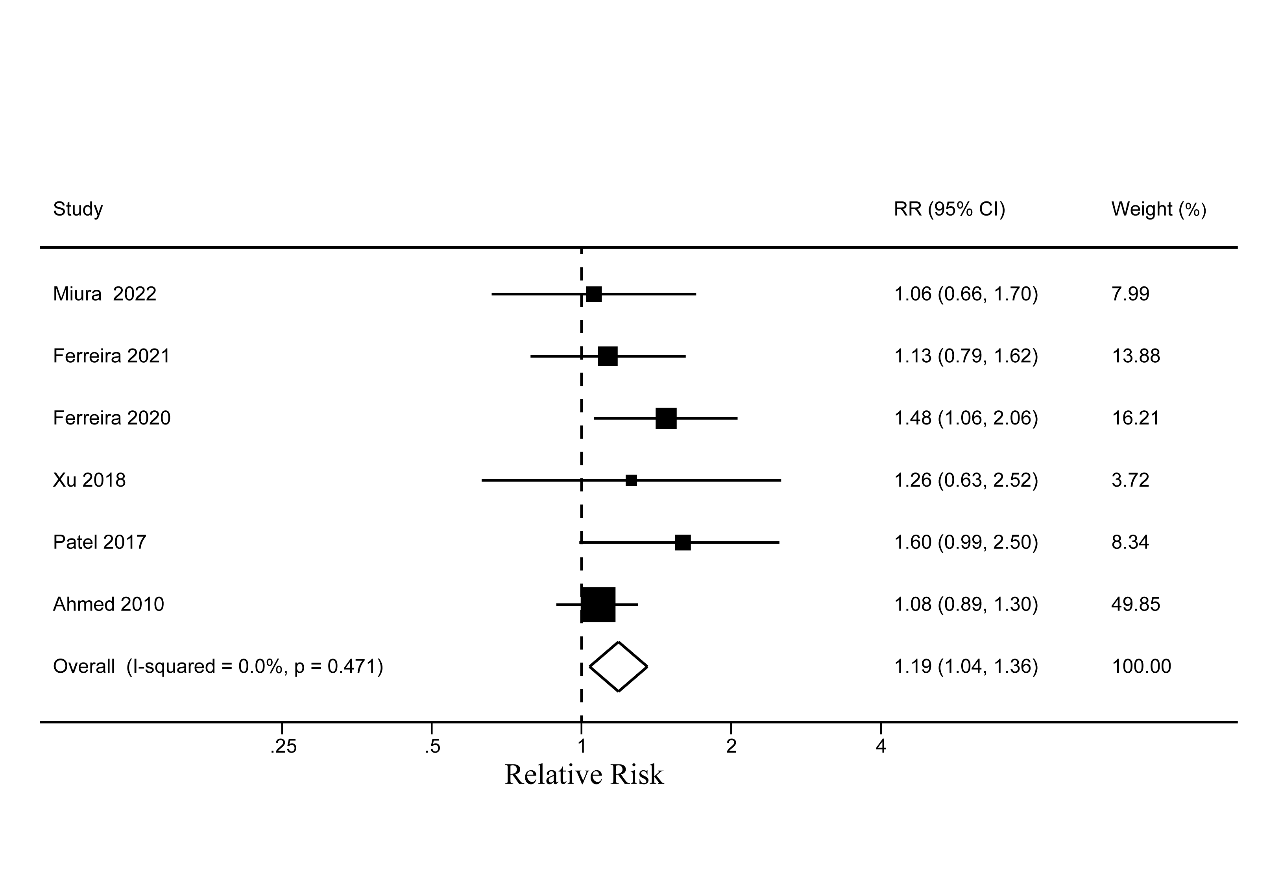


The size of the black squares reflects the relative statistical weight of study-specific estimate, horizontal lines indicate 95% CIs. The diamond indicates the pooled RR estimates with 95% CI. CI, confidence interval; RR, relative risk.

**Additional file 29: Figure 20.** Forest plot for association between blood potassium levels and risk of in-hospital mortality in patients with total cardiovascular diseases, expressed as comparison between hyperkalemia and normokalemia together with hypokalemia.


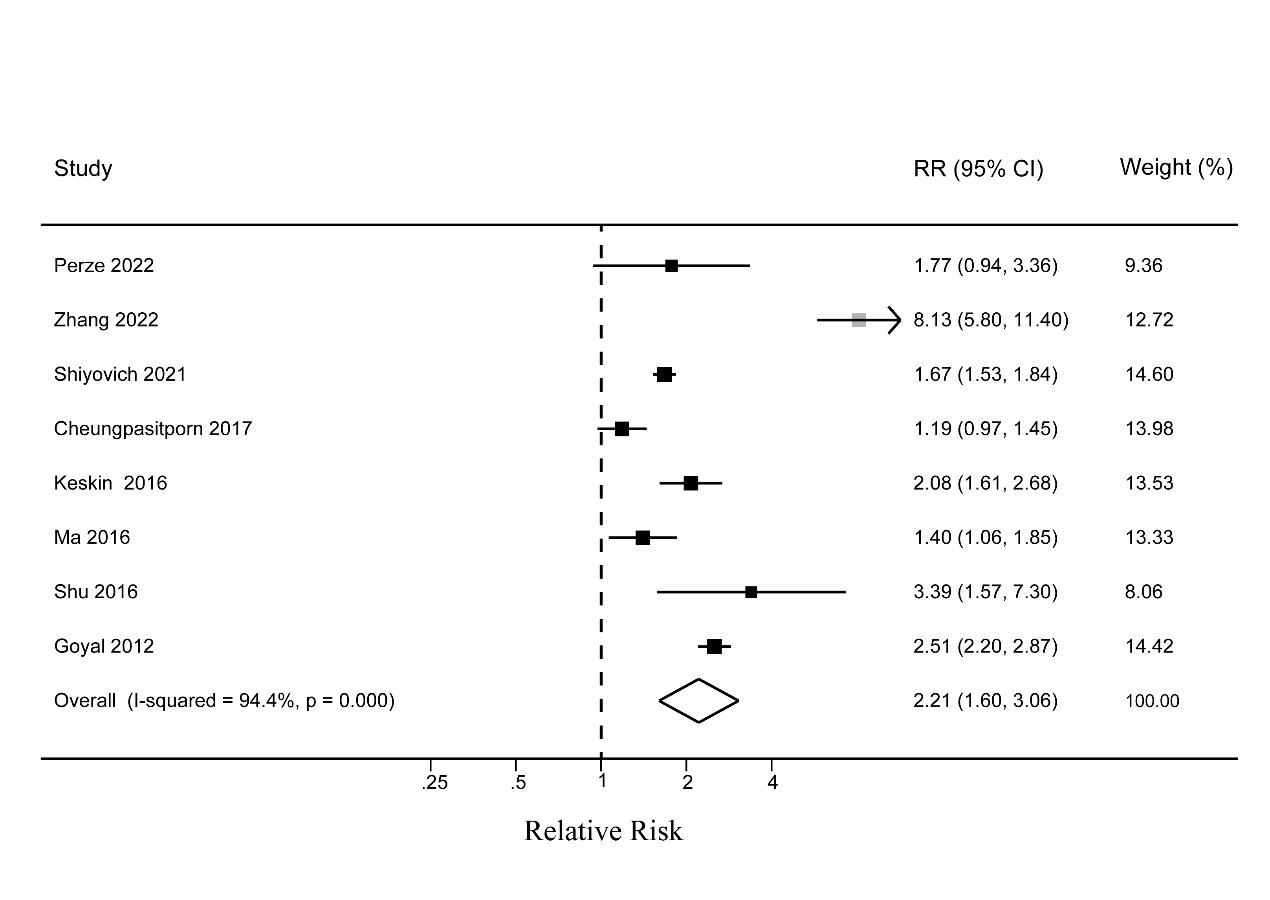


The size of the black squares reflects the relative statistical weight of study-specific estimate, horizontal lines indicate 95% CIs. The diamond indicates the pooled RR estimates with 95% CI. CI, confidence interval; RR, relative risk.

**Additional file 30: Figure 21.** Forest plot for association between blood potassium levels and risk of short-term mortality in patients with total cardiovascular diseases, expressed as comparison between hyperkalemia and normokalemia together with hypokalemia.

**
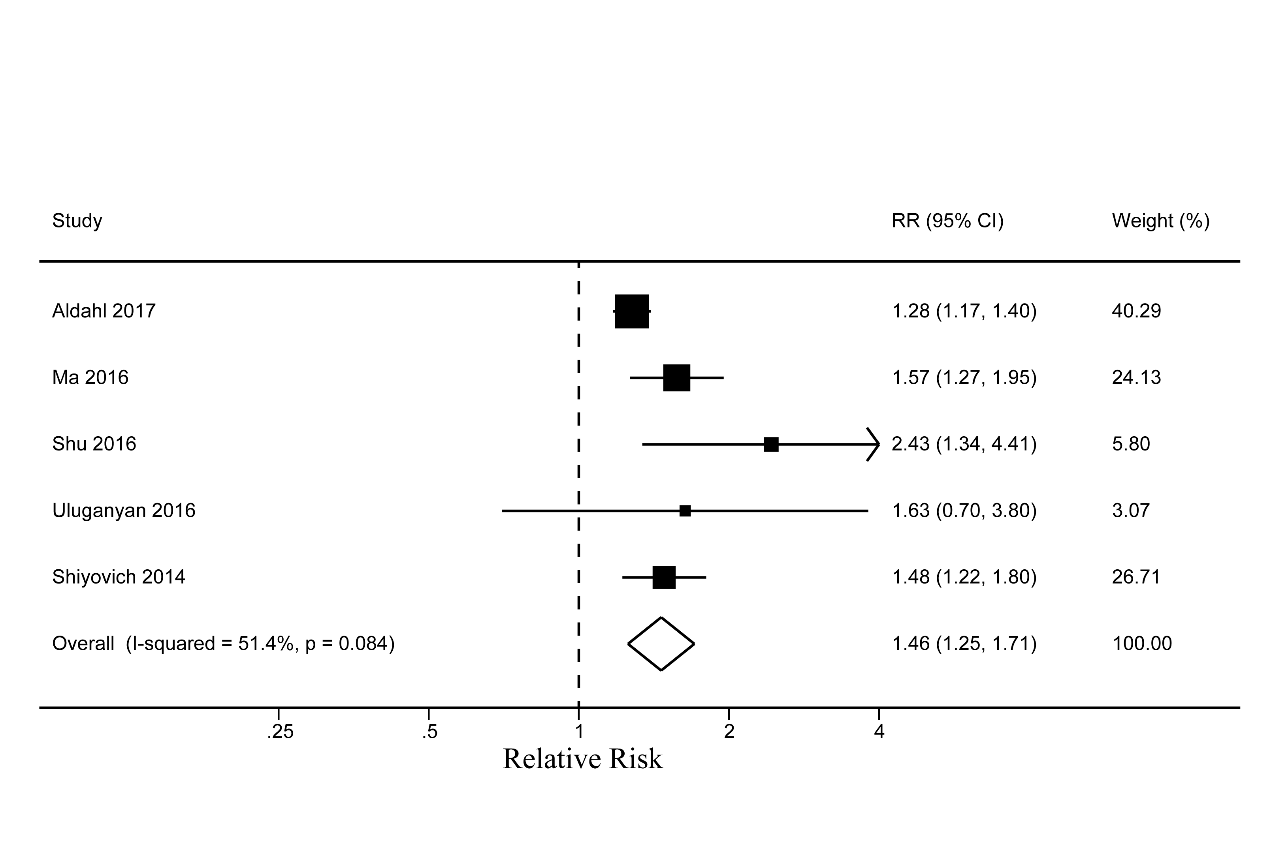
**

The size of the black squares reflects the relative statistical weight of study-specific estimate, horizontal lines indicate 95% CIs. The diamond indicates the pooled RR estimates with 95% CI. CI, confidence interval; RR, relative risk.

**Additional file 31: Figure 22.** Forest plot for association between blood potassium levels and risk of long-term mortality in patients with total cardiovascular diseases, expressed as comparison between hyperkalemia and normokalemia together with hypokalemia.

**
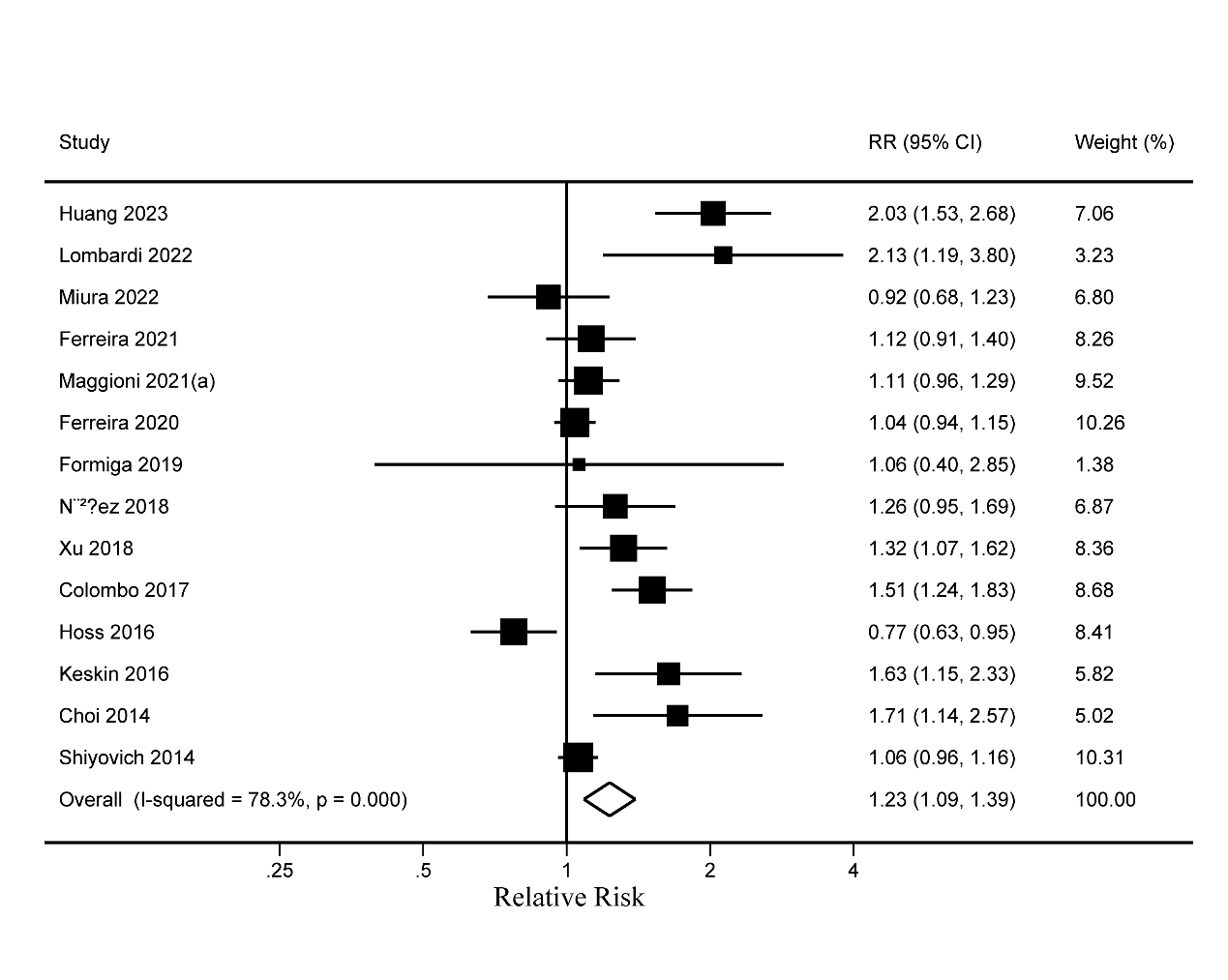
**

The size of the black squares reflects the relative statistical weight of study-specific estimate, horizontal lines indicate 95% CIs. The diamond indicates the pooled RR estimates with 95% CI. CI, confidence interval; RR, relative risk.

**Additional file 32: Figure 23**. Forest plot for association between blood potassium levels and risk of cardiovascular mortality in patients with total cardiovascular diseases, expressed as comparison between hyperkalemia and normokalemia together with hypokalemia.

**
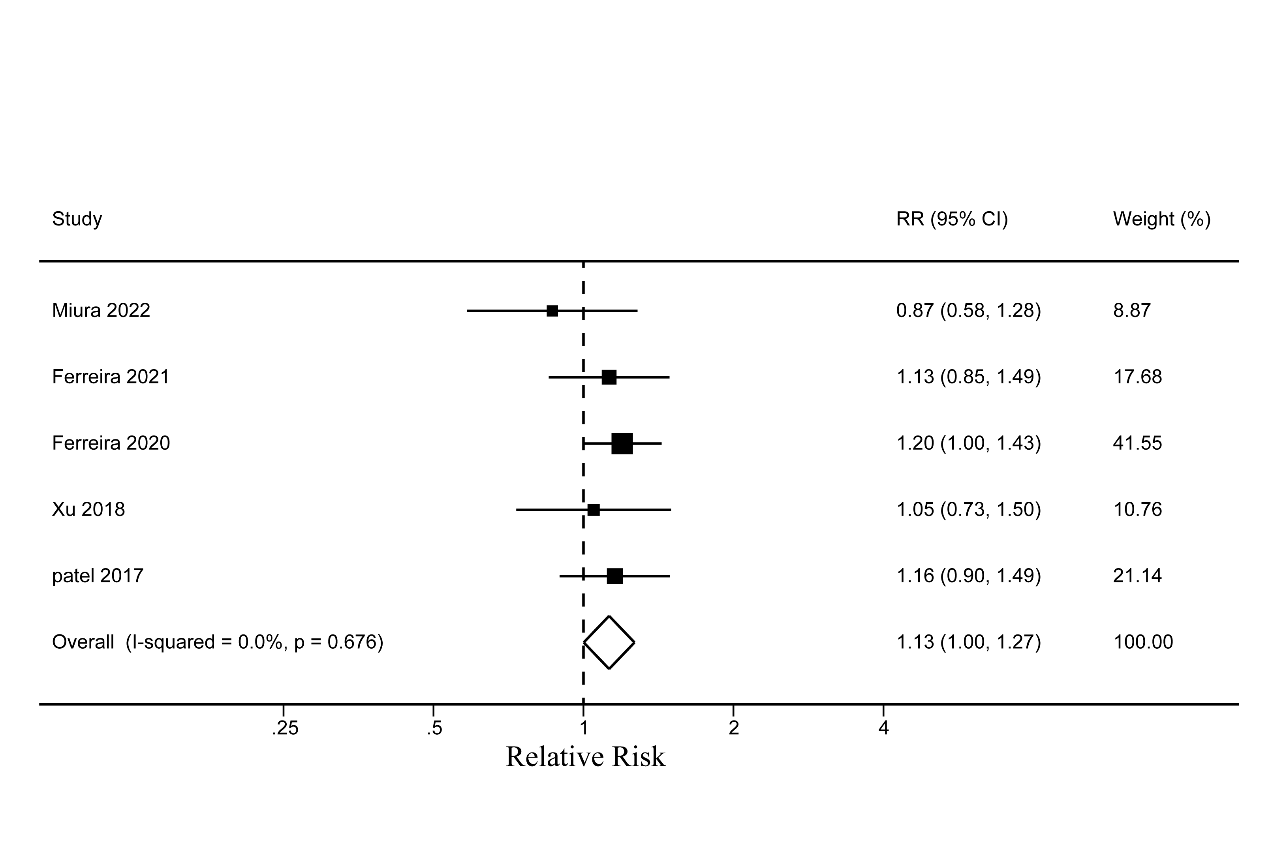
**The size of the black squares reflects the relative statistical weight of study-specific estimate, horizontal lines indicate 95% CIs. The diamond indicates the pooled RR estimates with 95% CI. CI, confidence interval; RR, relative risk.

**Additional file 33: Figure 24.** Sensitivity analyses for association between blood potassium levels and risk of all-cause and cardiovascular mortality in patients with total cardiovascular diseases, expressed as comparison between hypokalemia and hyperkalemia with normokalemia.


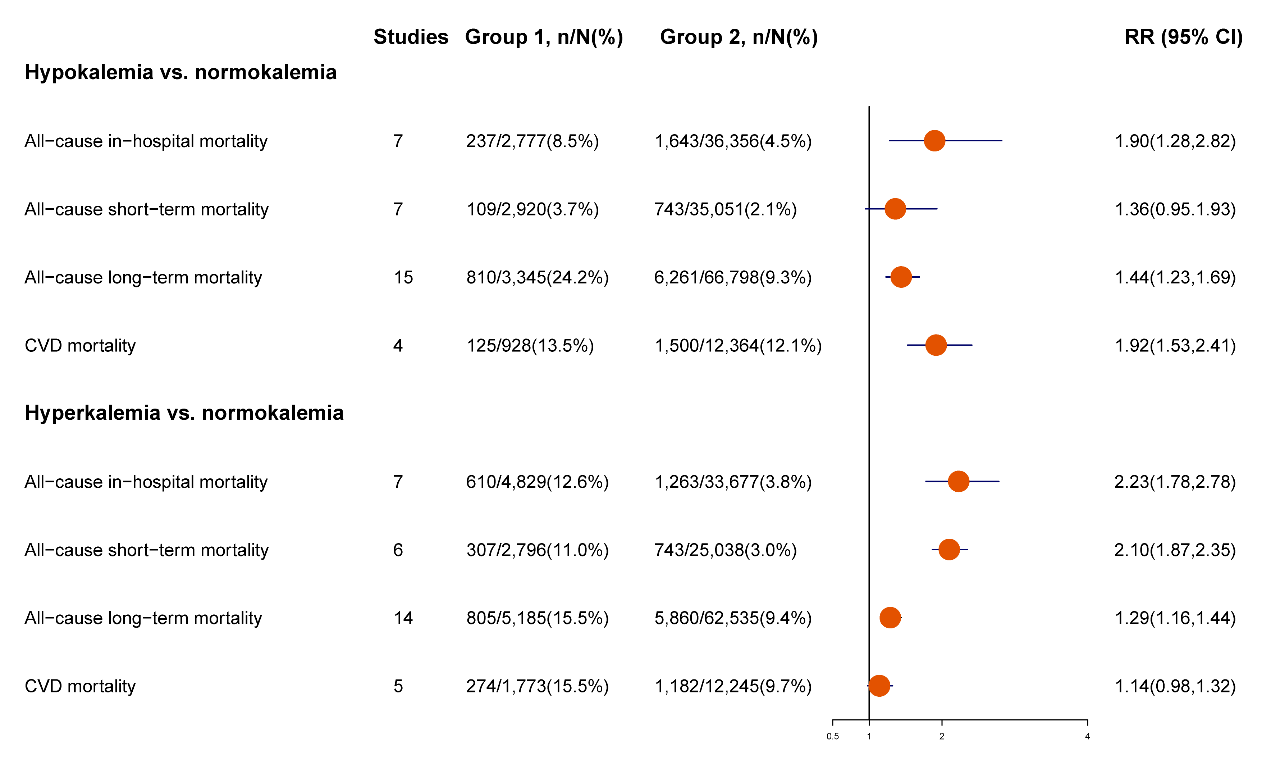


The diamond indicates the pooled RR estimates from random effects analysis, and horizontal lines indicate corresponding 95% CIs. CI, confidence interval; CVD, cardiovascular disease; RR, relative risk.

**Additional file 34: Figure 25.** Dose-response analysis for association of blood potassium levels with in-hospital mortality risk in patients with MI (A); short-term mortality risk in patients with MI (B); short-term mortality risk in patients with HF (C); long-term mortality risk in patients with MI (D); long-term mortality risk in patients with HF (E).

**
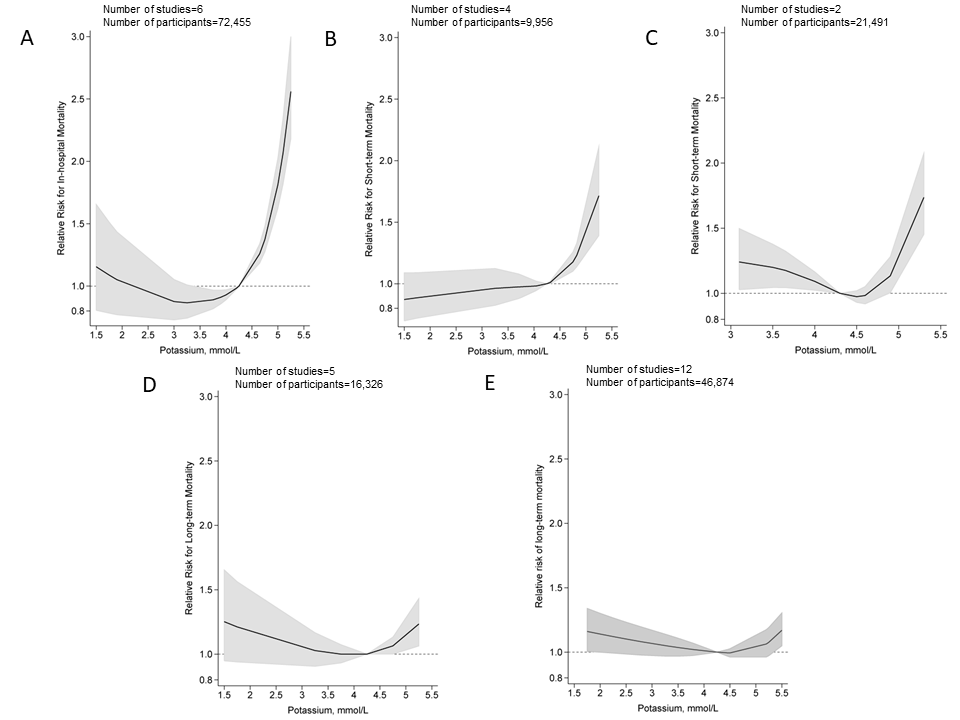
**

Solid lines represent summary relative risks; shaded areas are the corresponding 95% confidence intervals. CI, confidence interval; HF, heart failure; MI, myocardial infarction; RR, relative risk.

**Additional file 35:** **Figure 26.** Sensitivity analyses for association between hypokalemia and risk of all-cause mortality and cardiovascular mortality in patients with total cardiovascular diseases.


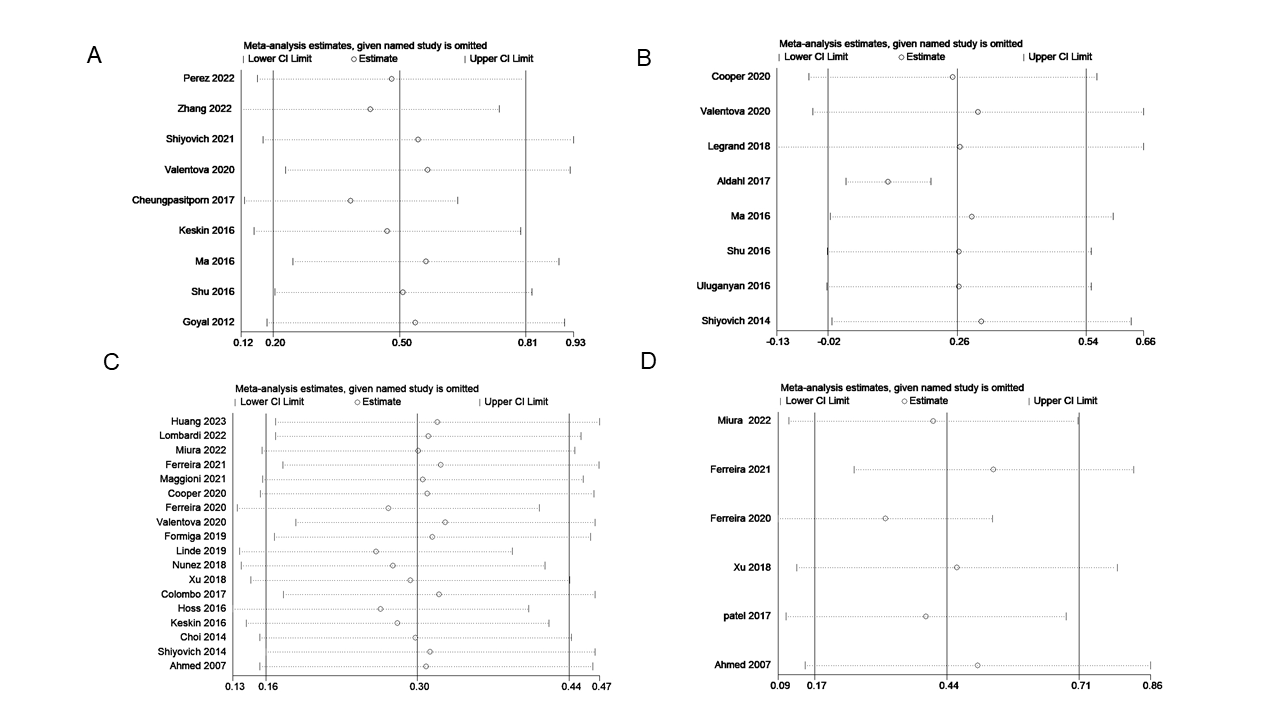


A, all-cause in-hospital mortality; B, all-cause short-term mortality; C, all-cause long-term mortality; D, cardiovascular mortality.

**Additional file 36: Figure 27.** Sensitivity analyses for association between hyperkalemia and risk of all-cause mortality and cardiovascular mortality in total cardiovascular diseases patients.


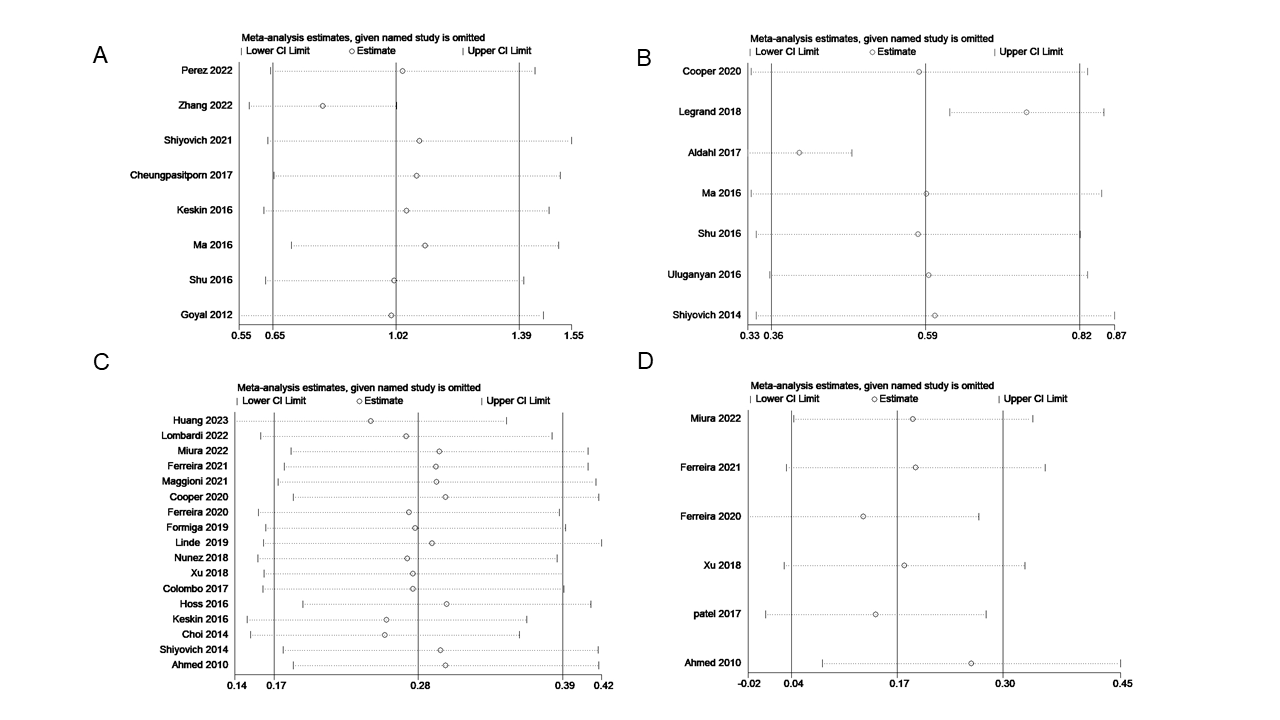


A, all-cause in-hospital mortality; B, all-cause short-term mortality; C, all-cause long-term mortality; D, cardiovascular mortality.

**Additional file 37: Figure 28.** Funnel plot of association between hypokalemia and hyperkalemia and risk of all-cause long-term mortality in patients with total cardiovascular diseases.

**
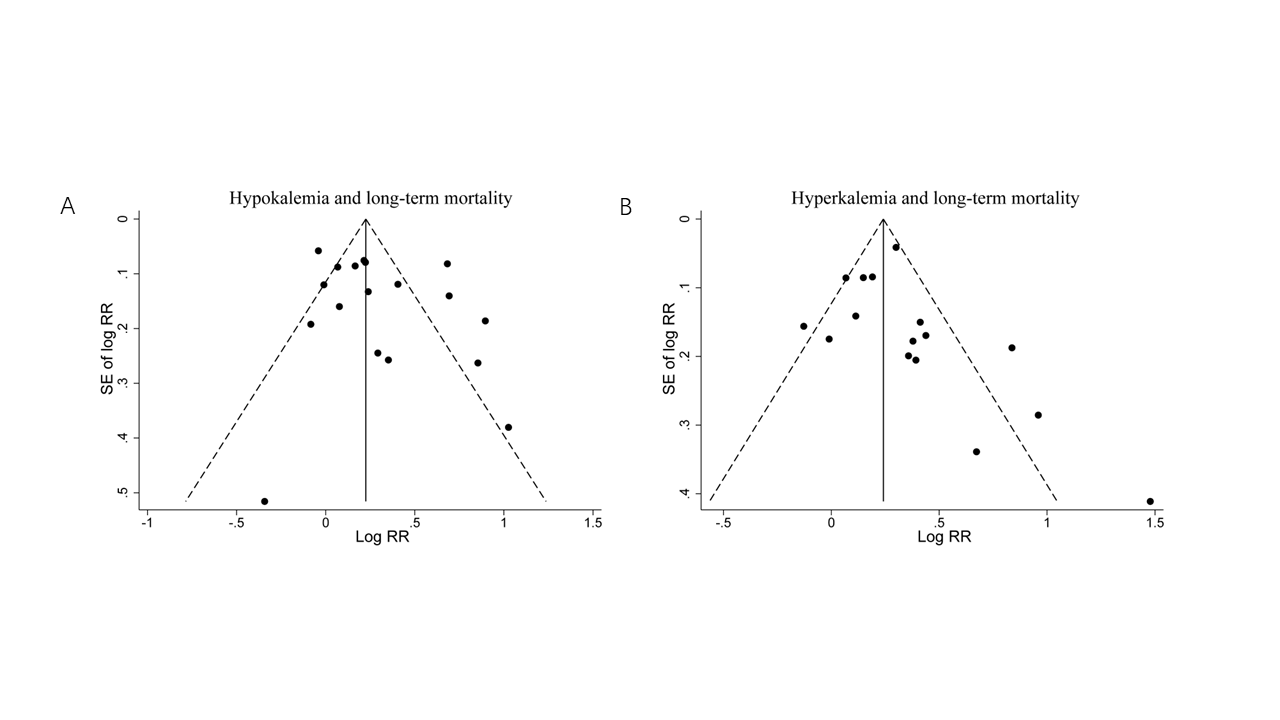
**

The vertical line represents the pooled RR. The dashed lines represent the pseudo-95% confidence interval of the RR. The circles represent risk estimates for each cohort. RR, relative risk. SE, standard error.
